# Mortality and heart failure hospitalizations in heart failure with preserved ejection fraction compared to heart failure with reduced ejection fraction: a systematic review and meta-analysis

**DOI:** 10.1093/eschf/xvag026

**Published:** 2026-01-16

**Authors:** Krzysztof Irlik, Julia Piaśnik, Mirela Hendel, Urszula Faron, Gregory Y H Lip, Katarzyna Nabrdalik, Konstantinos Prokopidis

**Affiliations:** Liverpool Centre for Cardiovascular Science at University of Liverpool, Liverpool John Moores University and Liverpool Heart & Chest Hospital, 6 W Derby St, Liverpool L7 8TX, UK; Doctoral School, Department of Internal Medicine, Diabetology and Nephrology, Faculty of Medical Sciences in Zabrze, Medical University of Silesia, Katowice, Poland; Students’ Scientific Association by the Department of Internal Medicine, Diabetology and Nephrology in Zabrze, Faculty of Medical Sciences in Zabrze, Medical University of Silesia, Katowice, Poland; Students’ Scientific Association by the Department of Internal Medicine, Diabetology and Nephrology in Zabrze, Faculty of Medical Sciences in Zabrze, Medical University of Silesia, Katowice, Poland; Students’ Scientific Association by the Department of Internal Medicine, Diabetology and Nephrology in Zabrze, Faculty of Medical Sciences in Zabrze, Medical University of Silesia, Katowice, Poland; Liverpool Centre for Cardiovascular Science at University of Liverpool, Liverpool John Moores University and Liverpool Heart & Chest Hospital, 6 W Derby St, Liverpool L7 8TX, UK; Department of Clinical Medicine, Aalborg University, Aalborg, Denmark; Liverpool Centre for Cardiovascular Science at University of Liverpool, Liverpool John Moores University and Liverpool Heart & Chest Hospital, 6 W Derby St, Liverpool L7 8TX, UK; Department of Internal Medicine, Diabetology and Nephrology, Faculty of Medical Sciences in Zabrze, Medical University of Silesia, Katowice, Poland; Liverpool Centre for Cardiovascular Science at University of Liverpool, Liverpool John Moores University and Liverpool Heart & Chest Hospital, 6 W Derby St, Liverpool L7 8TX, UK; Department of Musculoskeletal and Ageing Science, Institute of Life Course and Medical Sciences, University of Liverpool, 6 W Derby St, Liverpool L7 8TX, UK

**Keywords:** Heart failure, HFpEF, HFrEF, Mortality, Hospitalization, Meta-analysis

## Abstract

**Introduction:**

The global burden of heart failure (HF) is rising, with a shift towards more cases of heart failure with preserved ejection fraction (HFpEF). Given evolving epidemiology, an updated assessment of outcome differences between HFpEF and heart failure with reduced ejection fraction (HFrEF) is needed. This systematic review and meta-analysis aimed to provide a contemporary, large-scale comparison of clinical outcomes between HFpEF and HFrEF.

**Methods:**

A systematic review and meta-analysis were conducted to compare all-cause mortality, cardiovascular (CV) mortality, and HF hospitalizations in HFpEF (EF: >50%) and HFrEF (EF: <40%). Risk ratios (RR) and maximally adjusted hazard ratios (HR) with 95% confidence intervals (CI) were pooled using random-effects models. Additional analyses included prior HF hospital admissions, in-hospital mortality, and length of hospital stay.

**Results:**

A total of 101 studies were included. HFpEF patients had lower all-cause mortality [RR: 0.78; 95% CI: 0.69–0.88; *P* < .001; adjusted HR: 0.71; 95% CI: 0.62–0.80; *P* < .001; 112 vs 148 per 1000 patient-years (PY)], CV mortality [RR: 0.64; 95% CI: 0.53–0.79; *P* < .001; adjusted HR: 0.65; 95% CI: 0.56–0.75; *P* < .001; 73 vs 110 per 1000 PY], and HF hospitalizations [RR: 0.75; 95% CI: 0.63–0.91; *P* = .003; adjusted HR: 0.87; 95% CI: 0.78–0.98; *P* = .02; 171 vs 225 per 1000 PY] compared to HFrEF.

**Conclusion:**

HFpEF patients experience lower mortality and hospitalization risks than HFrEF patients, even after adjustment for confounders. However, high absolute event rates in HFpEF highlight the need for effective treatment strategies to improve outcomes.

PROSPERO registration ID: CRD42024619499.

## Graphical Abstract

**Graphical Abstract:**
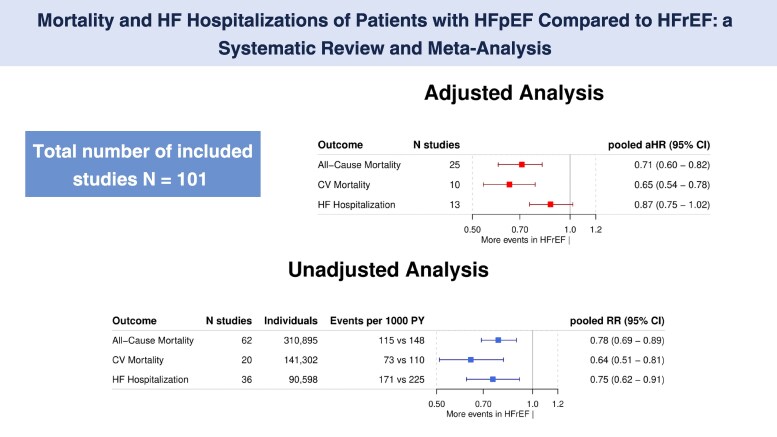
This illustration summarizes the meta-analysis findings, showing unadjusted risk ratios (RR) and adjusted hazard ratios (HR) with 95% confidence intervals for all-cause mortality, cardiovascular mortality, and heart failure hospitalizations. HFpEF patients had lower risks across all outcomes compared to HFrEF, even after adjustment for confounders, though absolute event rates remained high.

## Introduction

Heart failure (HF) is a significant contributor to global morbidity and mortality, presenting with two primary phenotypes: HF with preserved ejection fraction (HFpEF) and HF with reduced ejection fraction (HFrEF). These phenotypes differ in clinical characteristics and patient demographics. HFpEF predominantly impacts older adults, particularly women, who commonly present with comorbidities such as hypertension, diabetes, and obesity. In contrast, HFrEF is more prevalent among relatively younger people, particularly men, often associated with ischaemic heart disease.^1^

HFpEF and HFrEF are typically described as distinct pathophysiological entities with differing therapeutic responsiveness. Nevertheless, they present through the same clinical pathways, are managed by the same services, and together define the modern spectrum of heart failure encountered in routine care. Clarifying how their outcomes differ is relevant for prognostication, patient counselling, and forecasting healthcare burden and resource needs. While numerous studies have sought to delineate differences in outcomes between HFpEF and HFrEF,^2–5^ many of these investigations have been limited by their focus on specific geographic populations, restricting the generalizability of their findings. Moreover, the epidemiology of HF is evolving, with a notable shift towards an increasing prevalence of HFpEF, which now accounts for nearly half of all HF cases.^6,7^ This trend underscores the need for updated, comprehensive analyses to capture the current state of clinical outcomes in both HFpEF and HFrEF.

The primary aim of this meta-analysis is to provide a contemporary, large-scale, comprehensive comparison of outcomes between HFpEF and HFrEF across diverse international settings. The investigated outcomes are all-cause mortality, cardiovascular (CV) mortality, and HF hospitalizations. We also considered healthcare utilization and hospital-based outcomes, such as in-hospital mortality, prior HF hospital admissions, and length of hospital stay (LoS). By pooling a substantial number of studies with extensive patient data, we aim to generate robust estimates that enhance our understanding of HF phenotypes, inform clinical trial design, and improve public health strategies for HF globally.

## Methods

This systematic review and meta-analysis was conducted according to the Preferred Reporting Items for Systematic Reviews and Meta-Analyses (PRISMA) guidelines, to ensure a comprehensive and consistent analysis of all available evidence.^8^ The protocol was registered in the International Prospective Register of Systematic Reviews (PROSPERO) (CRD42024619499).

### Search strategy and study selection

Two independent reviewers searched PubMed, Scopus, Web of Science, and Cochrane Library from the beginning until November 2024. The full search strategies employed are shown in Supplementary Table S1. Discrepancies in the literature search process were resolved by a third investigator.

### Eligibility criteria

Studies were included based on the following criteria: (i) interventional, cohort, or cross-sectional studies (depending on the type of outcome) that either reported number of events for both HFrEF and HFpEF or reported adjusted hazard ratio (HR) for comparison of the HF phenotypes for any of the investigated outcomes; (ii) HFpEF defined as EF > 50% and HFrEF defined as EF < 40%; and (iii) patients aged 18 years and above. Published articles were excluded if they (i) were reviews, letters, *in vivo* or *in vitro* experiments, or commentaries; (ii) were not published as a full text and in English; and (iii) did not provide estimates or sufficient data to calculate estimates. For studies with multiple manuscripts, the manuscript with the most comprehensive dataset was included, and the others were excluded (duplicate data).

### Data collection process and variables abstracted

A standardized database was used to abstract data from included studies. Data extraction was performed by three independent reviewers. Discrepancies were resolved by consulting a fourth reviewer. The primary variables of interest included all-cause mortality, CV mortality, and HF hospitalizations. These longitudinal outcomes were abstracted along with follow-up duration. Secondary outcomes included in-hospital mortality, prior HF hospital admissions, and LoS. We also collected bibliographic information and details of study design, including observational vs post-hoc randomized study types. Additional data related to moderators, such as age, gender, left ventricular ejection fraction (LVEF), comorbid conditions, and HF treatment details, were also abstracted to facilitate meta-regression analyses. A full list of variables is available in the data dictionary (Supplementary Table S2).

### Risk of bias in individual studies

Risk of bias was assessed using the Newcastle-Ottawa Scale (NOS) for cohort studies and the modified NOS for cross-sectional studies.^9^ The NOS for cohort studies assigns a maximum of 9 points based on three quality parameters: selection, comparability, and outcome, and risk of bias was categorized as high (≤6 points), moderate (7–8), or low (9). NOS for cross-sectional studies assigns a maximum of 7 points based on similar quality parameters, and risk of bias was categorized as high (≤4 points), moderate (5–6), or low (7).

### Data synthesis

Pooled effect estimates were calculated using a random-effects model to account for expected heterogeneity between studies, with the inverse variance method used for pooling, tau² estimated by the restricted maximum-likelihood (REML) method, and Hartung–Knapp adjustment applied to derive confidence intervals for pooled effects. Risk ratios (RR) were calculated for data reported as proportions (e.g. mortality and readmissions), while mean differences (MD) were calculated for data reported as means (LoS). Estimates were reported as medians, and interquartile ranges (IQR) were converted to means and standard deviations (SD) using the Wan method^10^ and were subsequently pooled. Studies that did not provide SD or IQR were excluded. To investigate more reliable and homogenous results, we performed separate analyses based on the included studies utilizing adjusted models. Pooled HR were based on maximally adjusted HRs reported in individual studies. The inverse variance method was used to pool effect estimates, and the restricted maximum-likelihood estimator was used for tau². Confidence intervals for tau² and tau were calculated using the Q-profile method. For the main random-effects models, 95% prediction intervals were also calculated to describe the expected range of true effects in future comparable studies. Heterogeneity was assessed using the *I*² statistic and Cochran's *Q* test, while the potential for publication bias was assessed through visual inspection of a funnel plot and statistically using Egger’s test.^11^ Where funnel-plot asymmetry or Egger’s test suggested small-study effects, Duval and Tweedie trim-and-fill analyses were performed as a sensitivity assessment. Univariate meta-regression analyses were also conducted to explore the impact of potential moderators on outcome variability, reporting coefficients, standard errors, z-values, *P*-values, confidence intervals, Cochran's Q, R², and tau² to provide a detailed summary of each moderator's influence. We conducted prespecified subgroup analyses to explore potential sources of heterogeneity. Studies were stratified by follow-up duration (≤30 days; 31–365 days; 1–3 years; >3 years), by clinical setting at enrolment (inpatient vs outpatient), and by HF presentation at enrolment (acute decompensated vs chronic). To capture evolving treatment patterns, we also assigned each study to one of five therapeutic eras—pre-2000 (ACEi only), 2000–2004 [ACEi + BB + Mineralocorticoid Receptor Antagonists (MRA)], 2005–2014 [addition of ICD/Cardiac Resynchronization Therapy (CRT)], 2015–2019 [addition of Angiotensin Receptor-Neprilysin Inhibitors (ARNI)], and ≥2020 (addition of SGLT2i). For sensitivity analyses, we: (i) Excluded all studies rated as high risk of bias; (ii) Confined the therapeutic-era analyses to studies with less than 10 years’ duration to ensure accurate era classification; (iii) Redid the meta-analysis with subgroup analyses within general HF cohorts [excluding disease-, device- or procedure-specific studies like TAVI registry, coronary artery bypass graft vs percutaneous coronary intervention (PCI) study or chronic kidney disease as inclusion criteria] to minimize heterogeneity of outcomes. All analyses were performed in R software (version 4.4.2, R Foundation for Statistical Computing, 2020, Vienna, Austria). Statistical tests were two-sided, and significance was set at *P* < .05.

## Results

**Figure 1:**
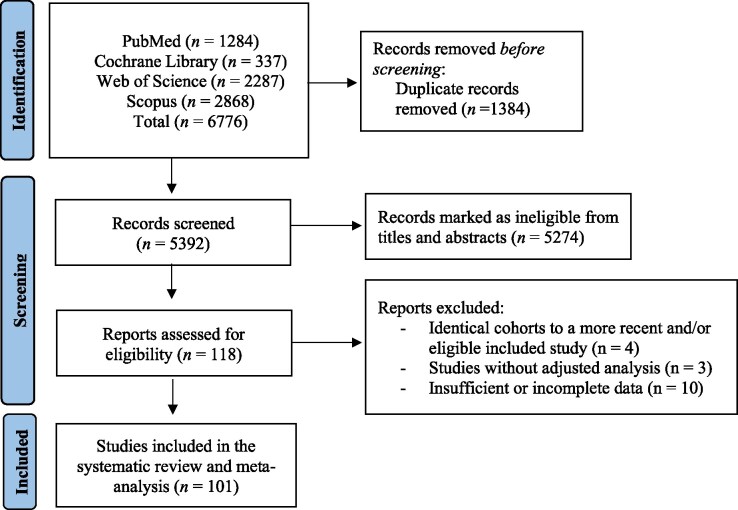
Study flowchart

Based on the employed search strategy, we found 6776 full texts in total. Out of those, 1384 records were removed as duplicates, and 5274 were removed due to ineligibility following title and abstract screening. From the remaining 118 articles, 10 had insufficient or incomplete data for analysis, four had identical cohorts with studies that were more recent and included in our study, and three studies did not utilize an adjusted analysis. Finally, 101 studies were included in this systematic review and meta-analysis (*Figure 1*). A complete list of included studies, grouped by outcome, is provided in Supplementary Table S3.

Patients with HFpEF were generally older, with a mean age of 74.3 years (SD: 12.1), and had a lower percentage of male patients (43.1%) compared to HFrEF patients, who had a mean age of 68.7 years (13.6) and were 58.6% male. Additionally, HFpEF patients had LVEF of 59.7% (8.0), whereas HFrEF patients had LVEF of 28.8% (8.1). Comorbidities were more frequently observed in HFpEF patients compared to those with HFrEF. Hypertension was more prevalent in HFpEF (70.3%) compared to HFrEF (54.3%). Similarly, atrial fibrillation (AF) was more common in HFpEF (42.4%) than in HFrEF (36.0%), and diabetes was reported in 35.2% of HFpEF patients vs 30.4% in HFrEF. In terms of HF severity, fewer HFpEF patients were classified as New York Heart Association class III or IV (24.6%) compared to HFrEF patients (30.2%). Included studies can be found in *Table 1*, and studies utilizing adjusted models along with covariates used in the model can be found in Supplementary Table S4.

**Table 1:** Characteristics of included studies

Study, year	Total *n* (M/F)	HFrEF			HFpEF			Duration of measured outcome
		*n* (M/F)	Age	LVEF (%)	*n* (M/F)	Age	LVEF (%)	
Abdul-Rahim, 2018	6593 (5012/1581)	5874 (4634/1240)	63.4 ± 10.9	26.4 ± 7.5	719 (378/341)	67.3 ± 10.4	58.5 ± 7.6	3.1 years
Aelst, 2018	79 (45/34)	41 (32/9)	65.3 ± 16.1	27.3 ± 10	38 (13/25)	78.7 ± 13.1	58.7 ± 9.2	1 year
Al-Jarallah, 2020	1743 (1076/667)	1268 (896/372)	61 ± 11		475 (180/295)	66 ± 11		1 year
Anastasio, 2022	154 (92/62)	85 (61/24)	69.6 ± 18.3	28 ± 6	69 (31/38)	77.9 ± 9.9	55 ± 5	1.2 years
Beale, 2019	430 (241/189)	160 (112/48)	75 ± 12	27 ± 6	270 (129/141)	80 ± 10	60 ± 6	
Bhatia, 2006	2450 (1285/1165)	1570 (983/587)	71.8 ± 12	25.9	880 (302/578)	75.4 ± 11.51	62.4	30 days, 1 year
Bhatt, 2024	31465 (14208/17257)	11660 (6917/4743)	76 ± 8.9	29 ± 8.9	19805 (7291/12514)	77.33 ± 9.64	59.33 ± 5.93	
Bonapace, 2019	3496 (2269/1227)	2213 (1690/523)	65.8 ± 12.8		1283 (579/704)	71.9 ± 13.1		
Borovac, 2019	209 (106/103)	123 (66/57)	72.6 ± 12	32.25 ± 4.41	86 (40/46)	72.1 ± 7.9	54.08 ± 5.15	1 year
Bouwmeester, 2022	108 (78/30)	75 (60/15)	68 ± 12.1	32.33 ± 9.07	37 (18/19)	74.7 ± 7.7	57.67 ± 3.08	1 year
Cenkerova, 2016	107 (69/38)	46 (35/11)	66.8 ± 12.1	26.6 ± 8.2	63 (34/29)	74.1 ± 9.8	59 ± 8.7	2 years
Chandra, 2019	11622 (7692/3930)	6887 (5398/1489)	66.5 ± 8.9	29.8 ± 6.2	4735 (2294/2441)	72.7 ± 8.4	57.5 ± 7.9	2.9 years
Chairat, 2020	414 (186/228)	127 (70/57)	59.1 ± 14.3	29 ± 7.7	287 (116/171)	64.1 ± 14.7	63.6 ± 11.7	1 year
Chung, 2019	2572 (1293/1279)	1808 (1022/786)	65.5 ± 14.7	27.9 ± 7.6	764 (271/493)	70 ± 13.3	60.7 ± 7.8	3.1 years
Cristobal, 2023	268 (140/128)	134 (93/41)	70 ± 12.7	32 ± 1.5	134 (47/87)	77 ± 8.2	62 ± 2.3	3.6 years
Cui, 2020	16626 (9990/6636)	11064 (7639/3425)	73.6 ± 12.3		5562 (2351/3211)	78.9 ± 9.9		
Dunlay, 2021	802 (442/360)	396 (286/110)	73.9 ± 15.9		406 (156/250)	78.6 ± 13.8		2 years
Eitel, 2019	521 (366/155)	188 (146/42)	65 ± 10.4		333 (220/113)	65.4 ± 9.6		
Farmakis, 2023	14072 (8693/5379)	8904 (6418/2486)	64 ± 14.1		5168 (2275/2893)	70 ± 13.3		1 year
Fischer-Rasokat, 2019	757 (346/411)	166 (113/53)	80 ± 6	29.7 ± 6.7	591 (233/358)	82.7 ± 5.2	63.3 ± 3.7	30 days, 1 year
Frohlich, 2019	8226 (6098/2128)	7080 (5406/1674)	66.6 ± 12.6		1146 (692/454)	65.9 ± 16.8		5.5 years
Fudim, 2020	441 (257/184)	225 (145/80)	62.67 ± 11.19	26.34 ± 10.45	216 (112/104)	69.34 ± 11.19	60.34 ± 6.72	2 years
Fujimoto, 2022	549 (387/162)	98 (82/16)	64.6 ± 10.5	31 ± 7.1	451 (305/146)	67 ± 10.1	62.9 ± 7.4	2.9 years
Ganapathi, 2020	1271 (764/507)	404 (316/88)	53.9 ± 12.8		867 (448/419)	48.8 ± 14.7		4.3 years
Gargani, 2021	296 (200/96)	199 (159/40)	69.33 ± 10.45	29.33 ± 8.96	97 (41/56)	73 ± 10.53	55 ± 7.52	1.2 years
Gierula, 2024	778 (344/434)	311 (181/130)	82 ± 10	36.7 ± 10.1	467 (163/304)	83.7 ± 8.6	56.2 ± 4	5.6 years
Gok, 2020	868	680			188			
Gomez-Otero, 2017	1193 (732/461)	583 (447/136)	68.2 ± 12.8		610 (285/325)	75 ± 10.7		30 days, 1 year
Gong, 2022	1183 (717/466)	598 (430/168)	58.3 ± 13.1	25 ± 7	585 (287/298)	58.1 ± 16	59 ± 6	2 years
Guo, 2022	121 (71/50)	51 (41/10)	68.4 ± 11.2	30.6 ± 10.45	70 (30/40)	70.3 ± 11.1	59.6 ± 6.4	1 year
Hage, 2020	121 (59/62)	75 (41/34)	65.67 ± 12.09	25 ± 7.56	46 (18/28)	76.33 ± 9.18	56.67 ± 3.83	30 days
Hamada, 2022	645 (317/328)	250 (163/87)	75 ± 11.18	29.33 ± 7.46	395 (154/241)	83.33 ± 8.93	62 ± 7.44	
Hamatani, 2018	1474 (903/571)	860 (612/248)	70 ± 14	27 ± 7	614 (291/323)	77 ± 11	59 ± 6	1.8 years
Hamazaki, 2020	1023 (656/367)	445 (322/123)	63.4 ± 15.2	28.6 ± 7	578 (334/244)	71.4 ± 10.8	61.5 ± 8.1	1.8 years
Huang, 2020	5391 (3924/1467)	4470 (3453/1017)	60.5 ± 12.6	27.9 ± 8.75	921 (471/450)	69 ± 13.1	57.2 ± 1.2	1 year
Huang, 2022	2246 (1480/766)	673 (518/155)	74.3 ± 14.4	29.3 ± 7.7	1573 (962/611)	81.1 ± 11.1	67.4 ± 10.3	2.3 years
Imamura, 2024	256 (127/129)	83 (50/33)	84 ± 8.3	39.7 ± 8.3	173 (77/96)	86.3 ± 9	61.7 ± 5.2	4.6 months
Ito, 2019	419 (282/137)	282 (216/66)	67.4 ± 14	27.7 ± 7.4	137 (66/71)	75.8 ± 9.0	61.6 ± 7.6	8.9 months
Iwatsu, 2022	448 (248/200)	187 (126/61)	71.3 ± 12	28.7 ± 8.2	261 (122/139)	77.7 ± 9.7	65.5 ± 9.3	2 years
Jarkovsky, 2020	1815 (1068/747)	1223 (805/418)	70 ± 13.4	28.7 ± 9.6	592 (263/329)	72.3 ± 11.9	55 ± 7.4	1 year
Jimenez-Marrero, 2020	626 (347/279)	348 (238/110)	70 ± 12	28 ± 7	278 (109/169)	75 ± 9	63 ± 8	1.25 years
Kamiya, 2021	1682 (948/734)	668 (460/208)	76.06 ± 7.25		1014 (488/526)	79.79 ± 7.2		2 years
Kaplon-Cieslicka, 2016	661 (440/221)	474 (364/110)	67 ± 13.38	29.33 ± 13.4	187 (76/111)	76.67 ± 11.21	55.33 ± 7.52	
Kaplon-Cieslicka, 2022	4869 (3116/1753)	3140 (2355/785)	66 ± 13.3	27.7 ± 9.6	1729 (761/968)	73 ± 12.6	56 ± 5.9	1 year
Kasahara, 2018	3635 (2421/1214)	742 (570/172)	66.8 ± 12.6	31.8 ± 6.3	2893 (1851/1042)	69.3 ± 12.2	65.2 ± 9.0	6.3 years
Kawahira, 2021	362 (199/163)	164 (116/48)	68 ± 14	31 ± 7	198 (83/115)	78 ± 9	61 ± 8	2.8 years
Kawakami, 2021	535 (319/216)	320 (211/109)	70.14 ± 12.77	28.63 ± 6.64	215 (108/107)	76.23 ± 10.39	63.81 ± 7.83	2.7 years
Kerwagen, 2023	1314 (909/405)	818 (636/182)	68.4 ± 11	30.4 ± 7.3	496 (273/223)	73 ± 9	56.5 ± 5.7	1 year
Kim, 2024	4532	3252			1280			
Kitai, 2020	3014 (1629/1385)	1383 (925/458)	73.8 ± 13.6	29.1 ± 7.1	1631 (704/927)	80.7 ± 9.9	61.9 ± 7.5	1.3 years
Kumar, 2023	1781 (859/922)	609 (387/222)	70.9 ± 15.9		1172 (472/700)	76.9 ± 13		4.6 years
Kusunose, 2023	192 (117/75)	99 (71/28)	68 ± 14	32 ± 7	93 (46/47)	71 ± 14	59 ± 7	4.8 years
Lala, 2018	223 (126/97)	157 (99/58)	68.88 ± 8.42	29.76 ± 7.30	66 (27/39)	69.57 ± 11.41	52.08 ± 4.76	
Lam, 2018	1783 (1309/474)	1209 (1009/200)	62.1 ± 13.2	26 ± 8.9	574 (300/274)	71.5 ± 11.8	60 ± 5.9	2 years
Lin, 2019	266 (227/39)	158 (122/36)	59 ± 7	28.5 ± 17.53	108 (105/3)	64.5 ± 16	64.53 ± 67	1.5 years
Lofman, 2017	31856 (20373/11483)	22981 (16343/6638)	72.27 ± 12.36		8875 (4030/4845)	78.29 ± 10.45		1 year, 5 years
Lopez-Azor, 2023	1141 (463/678)	190 (122/68)	74.3 ± 13.90		951 (341/610)	82.5 ± 9.2		
Lund, 2018	6276 (4272/2004)	4323 (3207/1116)	65 ± 11	29.3 ± 8.9	1953 (1065/888)	67 ± 11	58 ± 7.42	2.9 years
Lyu, 2019	554 (308/246)	211 (144/67)	60.67 ± 14.9		343 (164/179)	69.67 ± 11.9		1 year
Mansur, 2022	9436 (5083/4353)	4310 (2822/1488)	60.2 ± 13.7	28.7 ± 6.2	5146 (2261/2885)	66.9 ± 14.6	61.3 ± 6.3	
Migas, 2024	2101 (1078/1023)	596 (376/220)	74 ± 12.6		1505 (702/803)	77.7 ± 11.1		5 years
Miller, 2016	55 (39/16)	35 (30/5)	69 ± 14	27 ± 9	20 (9/11)	67 ± 12	61 ± 5	
Miro, 2023	7159 (3186/3973)	1674 (1094/580)	78.3 ± 10.39	30 ± 7.42	5485 (2092/3393)	82.3 ± 7.4	60.3 ± 8.16	1 year
Mirzai, 2023	177 (97/80)	79 (56/23)	70 ± 12	27 ± 8	98 (41/57)	71 ± 15	60 ± 7	4 years
Nakamaru, 2023	904 (538/366)	377 (282/95)	71 ± 13	29.9 ± 7.1	527 (256/271)	77.4 ± 12.1	61.8 ± 6.7	1 year
Nichols, 2015	5836 (3044/2792)	2205 (1411/794)	71.4 ± 13.8		3631 (1633/1998)	75.9 ± 12.3		1 year
Niedziela, 2024	7333 (4925/2408)	4947 (4009/938)	63.2 ± 12.5	25.2 ± 8.3	2386 (916/1470)	67.3 ± 13.1	54.6 ± 4.1	3.3 years
Ou, 2023	9187 (2720/6467)	5414 (694/4720)	78.83 ± 13.5	34 ± 4.45	3773 (2026/1747)	80.13 ± 11.64	58.3 ± 5.19	3 years
Pagnesi, 2023	1027 (695/332)	699 (529/170)	73.2 ± 11.7	27 ± 10.4	328 (166/162)	78.5 ± 10.1	55.67 ± 5.96	8.7 months
Pan, 2020	85 (44/41)	25 (18/7)	62.92 ± 16.79	33.23 ± 3.98	60 (26/34)	73.35 ± 11.31	57.19 ± 5.43	2.2 years
Popovic, 2018	239	198			41			2.1 years
Rywik, 2022	2270 (1598/672)	1608 (1307/301)	61.17 ± 11.06	25 ± 7.42	662 (291/371)	70.33 ± 15.08	59.67 ± 8.17	
Santas, 2020	2354 (1149/1205)	908 (635/273)	70 ± 12	31.3 ± 6.3	1446 (514/932)	76 ± 10	61.6 ± 7.4	2.6 years
Scrutinio, 2023	1547 (1127/420)	1168 (951/217)	65.3 ± 12.3	29.0 ± 6.6	379 (176/203)	73.6 ± 11.9	58.2 ± 5.5	
Seckin, 2023	420 (240/180)	244 (175/69)	70.24 ± 12.67	28.05 ± 6.69	176 (65/111)	77.65 ± 10.43	58.29 ± 6.54	1 year
Settergren, 2024	77189 (49319/27870)	53768 (38218/15550)	72.7 ± 12.6		23421 (11101/12320)	78.7 ± 9.3		5 years
Shiga, 2019	986 (512/474)	444 (292/152)	71 ± 15.6	29.3 ± 8.9	538 (220/318)	80 ± 11.15	59.3 ± 7.4	1.6 years
Shukkoor, 2021	359 (241/118)	296 (212/84)	59.1 ± 13.6		63 (29/34)	61.3 ± 13.5		1 year
Smeets, 2020	416	110			306			6 months
Song, 2020	325 (247/78)	215 (184/31)	57.9 ± 15.2	30.1 ± 5.3	110 (63/47)	69.4 ± 13.6	60.8 ± 6.2	1 year
Subki, 2020	1846 (982/864)	400 (281/119)			1446 (701/745)			
Takei, 2019	2921 (1728/1193)	1441 (1026/415)	70.9 ± 14.3	28.5 ± 6.9	1480 (702/778)	77.8 ± 11.4	60.1 ± 6.4	
Tay, 2023	14221 (7909/6312)	7341 (5188/2153)	66 ± 13.2		6880 (2721/4159)	73.4 ± 12.2		1 year, 2 years
Thuijs, 2020	1652 (1251/401)	74 (53/21)	67.0 ± 9.3	31.6 ± 4.2	1578 (1198/380)	65.9 ± 9.6	59.6 ± 6.6	3 years
Tomasoni, 2024	70542	49589			20953			1.9 years
Tromp, 2017	715 (521/194)	607 (470/137)	68 ± 12		108 (51/57)	74.4 ± 10.1		6 months
Tromp, 2024	469 (308/161)	354 (265/89)	65.7 ± 14		115 (43/72)	74.6 ± 8.8		1.5 months
Tsuji, 2017	2884 (1870/1014)	730 (560/170)	66.9 ± 12.7	31.1 ± 6.1	2154 (1310/844)	71.7 ± 10.9	64.8 ± 8.99	1year, 3 year
van Essen, 2022	4620 (2960/1660)	3180 (2270/910)	70.3 ± 11.6		1440 (690/750)	76.1 ± 9.9		6 months
Wang, 2017	1409 (1047/362)	207 (164/43)	64.2 ± 10.75	32.01 ± 5.74	1202 (883/319)	64.9 ± 10.52	63.48 ± 6.94	2.3 years
Wang, 2024	183116 (106565/76551)	82083 (56548/25535)	65.3 ± 13.7		101033 (50017/51016)	72.5 ± 12.1		30 days, 1 year, 3 years
Wernhart, 2023	276 (202/74)	153 (130/23)	52.8 ± 10.3	22.3 ± 7.2	123 (72/51)	60 ± 12.3	59.7 ± 4.7	2 years
Wierda, 2023	218 (136/82)	147 (107/40)	73 ± 11	32 ± 8	71 (29/42)	78 ± 9	57 ± 6	30 days, 1 year
Wu, 2023	145 (65/80)	33 (22/11)	68.36 ± 10.57	33 ± 4.6	75 (43/32)	75.16 ± 10.33	64.7 ± 5.3	3 months
Xu, 2014	185 (92/93)	82 (59/23)	58 ± 11		103 (33/70)	65 ± 10		1 year
Xu, 2020	311 (213/98)	202 (150/52)	63 ± 14	27 ± 6	109 (63/46)	75 ± 10	62 ± 6	1 year
Yaku, 2018	3295 (1785/1510)	1551 (1030/521)	76.33 ± 13.36		1744 (755/989)	82 ± 8.9		
Yoshikawa, 2020	3014 (1629/1385)	1383 (925/458)	75.67 ± 13.36	29.1 ± 7.1	1631 (704/927)	82 ± 8.9	61.9 ± 7.5	1 year
Zafrir, 2019	322 (176/146)	159 (125/34)	74.4 ± 10.8		163 (51/112)	79.7 ± 9.8		1 year, 2 years
Zeller, 2021	225 (182/43)	163 (132/31)	67.8 ± 14.0	30 ± 9	62 (50/12)	64.4 ± 15.6	58 ± 7	3.8 years
Zhirov, 2019	853 (480/373)	466 (346/120)	66.33 ± 12.64	33.33 ± 5.95	387 (134/253)	71 ± 11.16	60 ± 7.44	1 year

Data are expressed as mean ± standard deviation.

HFrEF, heart failure with reduced ejection fraction; HFpEF, heart failure with preserved ejection fraction; LVEF, left ventricular ejection fraction; M, males; and F, females

### Mortality

A total of 62 studies included data on all-cause mortality, reporting on 308 423 patients (HFpEF: 143 611; HFrEF: 164 812) with the mean follow-up duration of 748 days. In total, 67 459 deaths were recorded across these studies.

**Figure 2:**
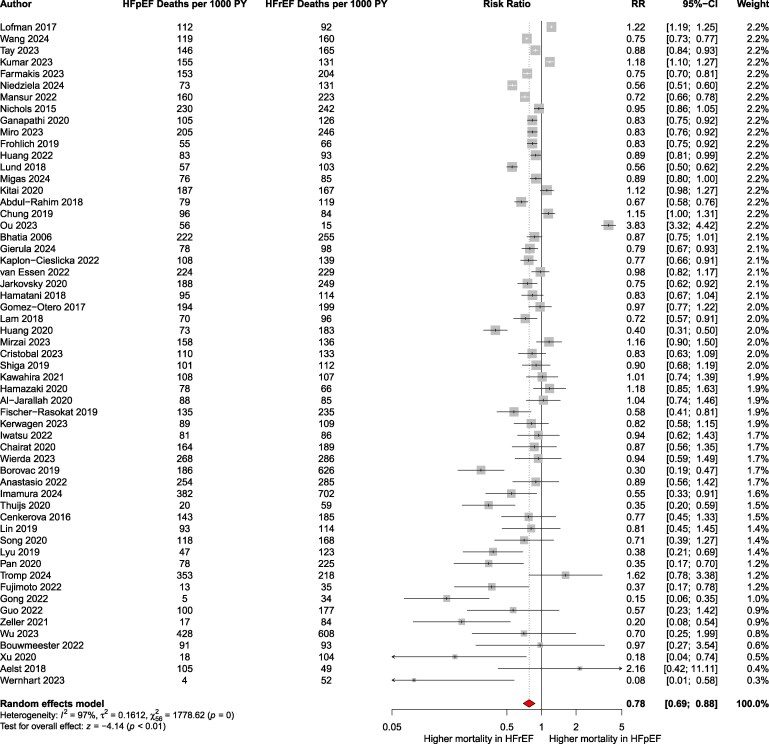
Pooled relative risk of all-cause mortality in HFpEF compared to that in HFrEF

**Figure 3:**
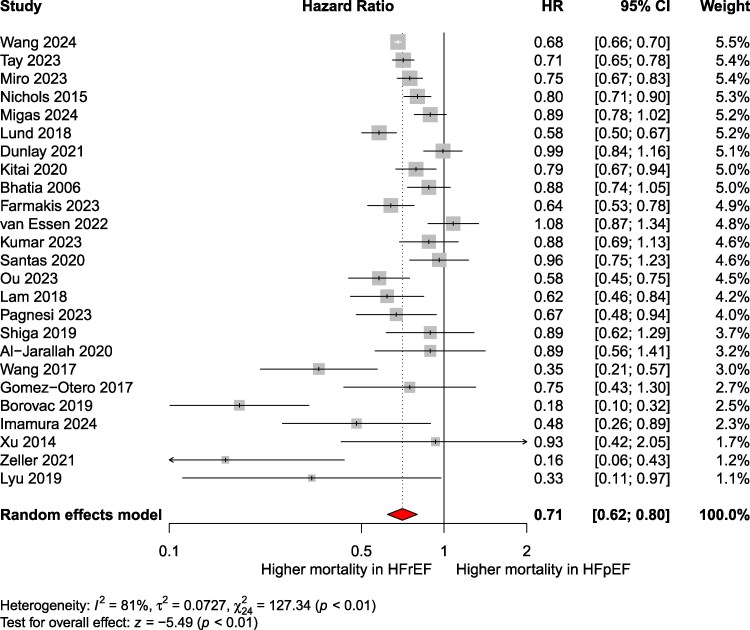
Pooled maximally adjusted hazard ratios for all-cause mortality in HFpEF compared to that in HFrEF

The pooled analysis demonstrated that HFpEF patients had a significantly lower risk of mortality compared to HFrEF patients [pooled RR: 0.78; 95% CI: 0.69–0.89; *P* < .001; *I*² = 97%; 95% PI: 0.35–1.76; *k* = 57 (Figure 2)]. In absolute terms, the death rate among patients with HFpEF was 113 per 1000 patient-years (95% CI: 94–132), compared to 148 (95% CI: 120–169) per 1000 patient-years among those with HFrEF. Egger’s test for funnel-plot asymmetry indicated potential publication bias (*P* = .01), and the funnel plot is presented in Supplementary Fig. S1. Trim-and-fill analysis suggested 18 imputed studies, with an adjusted pooled RR of 0.95 (95% CI: 0.82–1.11). After exclusion of studies with a high risk of bias, the results were similar (Supplementary Fig. S2). Additionally, maximally adjusted HRs were available for 25 studies. The pooled maximally adjusted HR across these studies was 0.71 (95% CI: 0.60–0.82; *P* < .001; *I*² = 81%; 95% PI: 0.40–1.25; *k* = 25) (Figure 3) with Egger’s test not indicating a risk of publication bias (*P* = .69) (Supplementary Fig. S3). Subgroup analyses by follow-up (*P* = .70) (Supplementary Fig. S4) and setting (inpatients vs outpatients; *P* = .83) (Supplementary Fig. S5) were non-significant. Analysis restricted to general HF cohorts (excluding device- or procedure-specific studies) confirmed lower mortality in HFpEF (RR: 0.80; 95% CI: 0.73–0.88; *P* < .0001; *I*² = 96.5%; *k* = 49) (Supplementary Fig. S6). Stratification by HF presentation at enrolment (acute vs chronic) gave RR 0.73 (95% CI: 0.64–0.83; *k* = 30) vs 0.92 (95% CI: 0.81–1.05; *k* = 19), with significant subgroup differences (*P* = .0094) (Supplementary Fig. S7). In therapeutic-era analyses, relative to HFrEF, HFpEF showed lower risk across successive strata defined by background therapy: ACEi alone (RR: 0.68), ACEi + BB + MRA (RR: 0.92), +ICD/CRT (RR: 0.75), and ARNI (RR: 0.76) (Supplementary Fig. S8). Notably, only one SGLT2i-era study was available.

### Cardiovascular mortality

Data on CV mortality were reported by 20 studies encompassing 141 302 patients (HFpEF: 49 454; HFrEF: 91 848).

**Figure 4:**
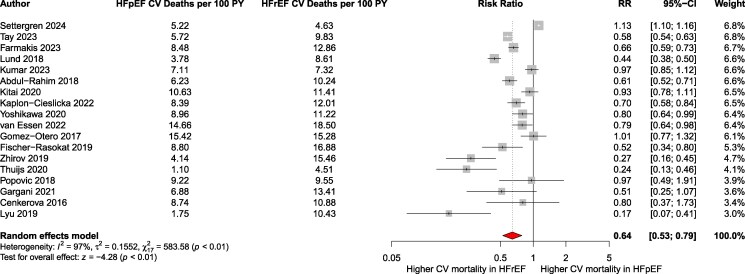
Pooled relative risk of cardiovascular mortality in HFpEF compared to that in HFrEF

**Figure 5:**
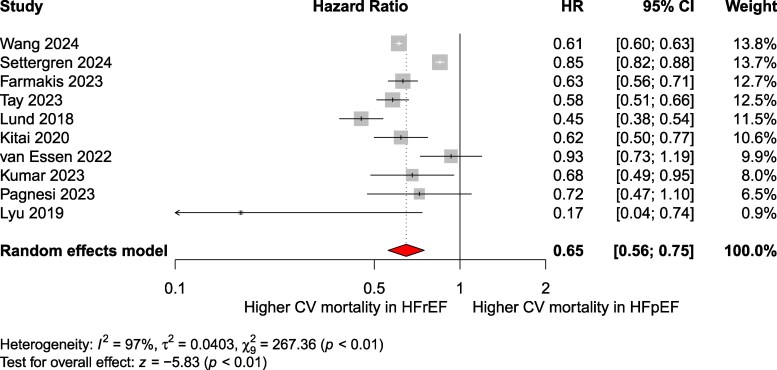
Pooled maximally adjusted hazard ratios for cardiovascular mortality in HFpEF compared to those in HFrEF

The pooled analysis revealed a significantly lower risk of CV mortality in HFpEF compared to HFrEF (pooled RR: 0.64; 95% CI: 0.51–0.81; *P* < .001; *I*² = 97.1%; 95% PI: 0.27–1.52; *k* = 18) (Figure 4). The CV mortality rate in patients with HFpEF was 73 per 1000 patient-years (95% CI: 54–92), while in those with HFrEF, it was 110 per 1000 patient-years (95% CI: 92–129). In total, 28 428 CV deaths were recorded. Egger’s test indicated potential publication bias (*P* < .01) (Supplementary Fig. S9). Trim-and-fill analysis suggested 10 imputed studies, with an adjusted RR of 1.05 (95% CI: 0.74–1.47). Additionally, the pooled maximally adjusted HR across 10 studies was 0.65 (95% CI: 0.54–0.78; *P* < .001; *I*² = 96.6%; 95% PI: 0.39–1.06, *k* = 10) (Figure 5). Subgroup analysis by follow-up duration (*P* = .33) (Supplementary Fig. S10) and exclusion of a high-risk-of-bias study (Supplementary Fig. S11) yielded consistent results. Sensitivity analysis restricted to general HF cohorts confirmed lower CV mortality in HFpEF (RR: 0.72; 95% CI: 0.59–0.89; *P* = .0045; *I*² = 97.4%; *k* = 15) (Supplementary Fig. S12). Acute vs chronic HF stratification yielded RRs of 0.65 (95% CI: 0.47–0.91; *k* = 10) vs 0.83 (95% CI: 0.70–0.99; *k* = 5); between-group *P* = .13 (Supplementary Fig. S13). Therapeutic-era RRs were 0.52 (ACEi-only era; *k* = 2), 0.65 (ACEi + BB + MRA ± ICD/CRT era; *k* = 11), and 0.27 (ARNI/ACEi + BB + MRA ± ICD/CRT era; k = 1); subgroup *P* = .012 (Supplementary Fig. S14).

### Heart failure hospitalization

Data on HF hospitalizations were available from 36 studies, involving a total of 90 598 patients (HFpEF: 34 819; HFrEF: 55 779).

**Figure 6:**
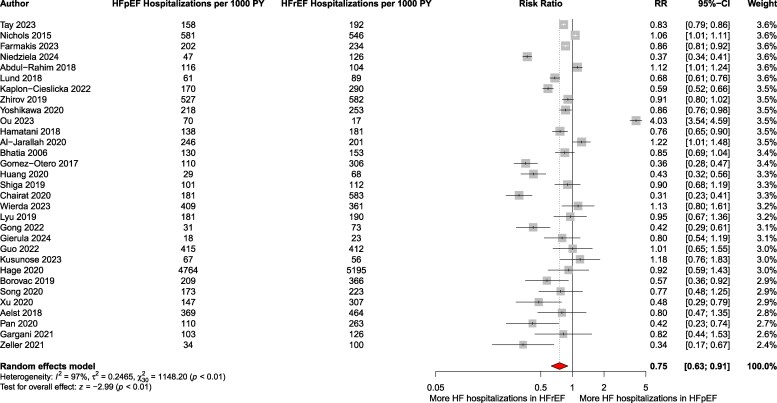
Pooled relative risk of HF hospitalizations in HFpEF compared to that in HFrEF

**Figure 7:**
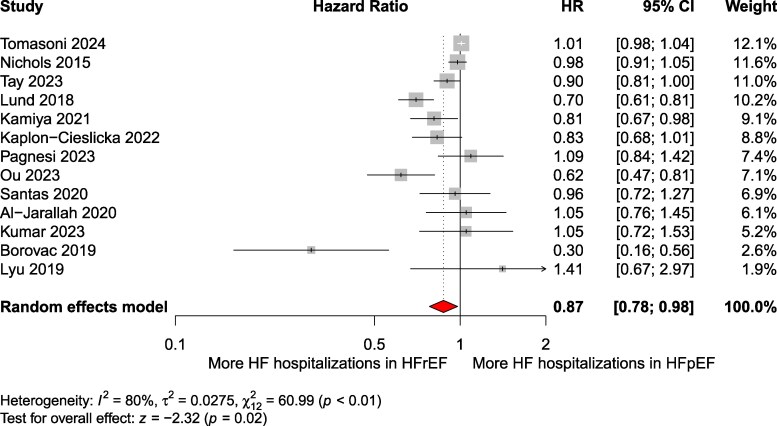
Pooled maximally adjusted hazard ratios for HF hospitalizations in HFpEF compared to those in HFrEF

The pooled analysis showed a significantly lower risk of HF hospitalization in patients with HFpEF compared to HFrEF (pooled RR: 0.75; 95% CI: 0.62–0.91; *P* = .005; *I*² = 97.4%; 95% PI: 0.27–2.12, *k* = 31) (Figure 6). Patients with HFpEF experienced a HF hospitalization rate of 171 per 1000 patient-years (95% CI: 118–225), whereas those with HFrEF had a rate of 225 per 1000 patient-years (95% CI: 166–284). A total of 24 145 HF hospitalization events were recorded across the included studies. Egger’s test did not indicate significant publication bias (*P* = .36), and the funnel plot is presented in Supplementary Fig. S15. Notably, the pooled maximally adjusted HR across 13 studies was 0.87 [95% CI: 0.75–1.02; *P* = .08; *I*² = 80.3%; 95% PI: 0.59–1.29, *k* = 13 (Figure 7)]. Subgroup analysis by follow-up duration (*P* = .95) did not show significant differences (Supplementary Fig. S16), however, clinical settings at recruitment (inpatients vs outpatients) had an impact on the outcomes (*P* = .01) (Supplementary Fig. S17). Exclusion of studies with high risk of bias yielded similar results with even better prognosis in HFpEF (pooled RR: 0.68; 95% CI: 0.57–0.80; *P* < .001; I² = 94.4%) (Supplementary Fig. S18). Sensitivity analysis restricted to general HF cohorts (excluding e.g. TAVI or PCI studies) showed RR 0.70 (95% CI: 0.60–0.82; *P* < .0001; *I*² = 95.6%) (Supplementary Fig. S19), stratification by HF presentation at enrolment (acute decompensated vs chronic HF) showed no difference (*P* = .96) (Supplementary Fig. S20). Across therapeutic eras—ACE inhibitor alone; ACEi + beta-blocker + MRA ± ICD/CRT; and ARNI addition to therapy—HF hospitalization risk remained similar (*P* = .16; Supplementary Fig. S21).

### In-hospital mortality

A total of 13 studies, covering 40 213 patients (HFpEF: 16 956; HFrEF: 23 257), provided data on in-hospital mortality.

The pooled analysis indicated a significantly lower risk of in-hospital mortality for HFpEF compared to HFrEF patients (pooled RR: 0.73; 95% CI: 0.58–0.91; *P* − = .009; *I*² = 61.3%; 95% PI: 0.42–1.26, *k* = 13) (Supplementary Fig. S22). A total of 1633 in-hospital deaths were recorded across the studies. Egger’s test for funnel-plot asymmetry did not reveal significant publication bias (*P* = .51). The funnel plot is presented in Supplementary Fig. S23. Exclusion of studies with high risk of bias provided consistent results (Supplementary Fig. S24).

### Prior heart failure hospital admissions

Data on prior HF hospital admissions were reported in 25 studies, encompassing 231 961 patients (HFpEF: 124 899; HFrEF: 107 062).

The pooled analysis demonstrated a significantly lower risk of prior HF admissions for HFpEF patients compared to HFrEF patients (pooled RR: 0.76; 95% CI: 0.67–0.86; *P* < .001; *I*² = 98.9%; 95% PI: 0.41–1.39) (Supplementary Fig. S25). In total, 56 637 prior HF admission events were recorded. Egger’s test indicated potential publication bias (*P* < .01). The funnel plot is displayed in Supplementary Fig. S26. Trim-and-fill analysis suggested 13 imputed studies, with an adjusted pooled RR of 0.50 (0.41–0.61). Sensitivity analysis excluding studies with a high risk of bias showed similar results (Supplementary Fig. S27).

### Length of hospital stay

LoS was assessed in 21 studies, including a total of 268 852 patients (HFpEF: 144 224; HFrEF: 124 628).

The pooled analysis showed no significant difference in LoS between HFpEF and HFrEF patients (MD: −0.19; 95% CI: −0.64 to 0.25; *P* = .37; *I*² = 92.1%; 95% PI: −2.02 to 1.64) (Supplementary Fig. S28). Egger’s test indicated no significant publication bias (*P* = .43). The funnel plot is shown in Supplementary Fig. S29. After exclusion of studies with high risk of bias, the results remained unchanged (Supplementary Fig. S30).

### Meta-regression analysis

The meta-regression analysis revealed several significant moderators influencing the outcomes across different domains. For all-cause mortality, age (*P* = .018) (Supplementary Fig. S31) and gender (*P* = .002) (Supplementary Fig. S32) were significant contributors. CV mortality was influenced by N-terminal pro b-type natriuretic peptide (NT-proBNP) levels (*P* < .001) (Supplementary Fig. S33), the presence of diabetes (*P* = .04) (Supplementary Fig. S34), and differences in LVEF between HF phenotypes (*P* = .04) (Supplementary Fig. S35). HF hospitalization rates were significantly moderated by age (*P* = .011) (Supplementary Fig. S36), gender (*P* = .015) (Supplementary Fig. S37), and diuretic use (*P* = .009) (Supplementary Fig. S38), with an *R*² of 56%. In the context of prior HF hospital admissions, body mass index (BMI) was found to be a significant moderator (*P* = .034) (Supplementary Fig. S39). Details regarding meta-regression results are provided in Supplementary Table S5.

### Risk of bias

Eighteen studies were evaluated as having a high risk of bias, while 47 studies had a moderate risk of bias. Details are presented in Supplementary Table S6 for cohort studies and Supplementary Table S7 for cross-sectional studies. Studies classified as having a high risk of bias most commonly scored poorly in several domains: first, selection and representativeness, where cohorts were drawn from narrowly selected populations rather than broad, consecutive HF populations; second, comparability, where limited or absent multivariable adjustment increased the likelihood of residual confounding; third, outcome assessment, where key outcomes were not clearly defined or not consistently assessed; and fourth, adequacy of follow-up, where outcome data had a high degree of missingness. These issues indicate that the dominant threats to validity in the included evidence base were selection bias, incomplete control of confounding, unclear outcome definition/ascertainment, and incomplete follow-up.

## Discussion

This comprehensive meta-analysis of over 300 000 patients demonstrates consistently better clinical outcomes in HFpEF compared to HFrEF across all-cause mortality, CV mortality, and HF hospitalizations. The pooled maximally adjusted HRs strengthen the confidence in these findings by accounting for confounders; however, the adjusted HR for HF hospitalization became borderline and non-significant after Hartung–Knapp correction, indicating that this particular difference is statistically fragile and should be interpreted cautiously. These findings are consistent with previous observations, such as the MAGGIC collaboration,^4^ but provide a more contemporary perspective given the changing prevalence of HF phenotypes. Importantly, this meta-analysis represents the largest compilation of data for this specific objective.

The absolute mortality rates observed in our analysis (112 vs 148 per 1000 patient-years for HFpEF and HFrEF, respectively) highlight the persistent high risk in both phenotypes, despite advances in therapy. This observation is particularly striking for HFpEF, where the presented real-world mortality rates substantially exceed those reported in contemporary randomized trials and prospective cohort studies. For instance, the DELIVER trial reported an all-cause mortality rate of 74 per 1000 patient-years,^13^ while EMPEROR-Preserved demonstrated 66 per 1000 patient-years.^14^ Similarly, a prospective cohort study by Lam et al.^5^ reported comparable death rates of 75 per 1000 patient-years, possibly reflecting participation bias with enrolment of younger, more stable patients. In contrast, our meta-analysis captured a broader, potentially more representative population spectrum, including post-hospitalization patients and registry-based cohorts with older age and higher comorbidity burden. This disparity between controlled studies and real-world outcomes underscores the importance of considering age and comorbidity profiles when interpreting HFpEF trial results and their generalizability to clinical practice.

When stratified by successive guideline-directed medical therapy eras—ACE inhibitor alone; ACEi + β-blocker + MRA; addition of ICD/CRT; introduction of ARNI; and incorporation of SGLT2 inhibitors—the relative risks comparing HFpEF to HFrEF for all-cause mortality, CV mortality, and HF hospitalizations showed no clear temporal trend, suggesting that evolving treatment landscapes contributed to heterogeneity but did not fundamentally change the comparative prognosis between these two phenotypes. A systematic network meta-analysis by Tromp et al.^15^ of 75 randomized HFrEF trials (*n* = 95 444) found that the greatest mortality benefit was achieved with the combination of ARNi, β-blocker, MRA, and SGLT2-inhibitor therapy. Although most of these agents have not demonstrated clear benefit in HFpEF, their use in HFrEF produces substantial survival gains—yet the inherent prognostic advantage of HFpEF remains unchanged across treatment eras in the current study. The lack of a clear era-related divergence in HF hospitalization can be interpreted as follows. First, advances in guideline-directed therapy for HFrEF have prolonged survival,^16^ so more recent HFrEF cohorts consist of older, more comorbid survivors with persistent susceptibility to decompensation and readmission despite better pharmacologic and device therapy.^17^ Second, HFpEF is now being recognized and labelled more systematically, particularly in obese, hypertensive, and multimorbid patients who are still underdiagnosed.^18^ As a result, it is likely that a greater share of HFpEF patients in contemporary cohorts are identified at an earlier stage of the disease. Third, although disease-modifying therapy in HFrEF has intensified across eras, management of the comorbid drivers that dominate HFpEF has also improved.^19,20^ These parallel gains could stabilize both phenotypes through different mechanisms, so the relative difference in HF hospitalization between HFpEF and HFrEF remains broadly similar across eras even as absolute survival improves.

Limiting analyses to general HF cohorts (excluding device-, procedure-, or disease-specific populations) yielded similar effect estimates, and in patients acute decompensated at enrolment vs chronic HF, the mortality benefit of HFpEF over HFrEF was attenuated in the acute setting, indicating comparable short-term outcomes across phenotypes. The relative differences in all-cause mortality, CV mortality, and HF hospitalizations between HFpEF and HFrEF remained consistent across widely varying follow-up periods, ranging from 30 days to over 3 years. This temporal stability of the outcome differences suggests that the better prognosis in HFpEF persists both in the vulnerable early post-discharge period and during longer-term follow-up. This finding is strongly supported by recent data from the China Cardiovascular Association (CCA) Database-Heart Failure Centre Registry,^12^ which demonstrated remarkably consistent all-cause mortality risk ratios between HFpEF and HFrEF across 30-day, 1-year, and 3-year follow-up periods, closely aligning with our meta-analysis results. The consistency of this relationship across time frames suggests that the underlying pathophysiological and clinical factors driving the mortality gap between HFpEF and HFrEF may be relatively stable throughout the disease course.

### Meta-regression insights

Meta-regression analyses identified key factors that moderate the mortality difference between HFpEF and HFrEF. NT-proBNP levels emerged as the strongest moderator of the relative CV mortality risk, with higher NT-proBNP levels associated with greater mortality disadvantage in HFrEF compared to HFpEF. This finding adds a new perspective to previous studies^5,21^ were it was found that NT-proBNP conferred similar relative risk information across phenotypes, with one of them^21^ notably demonstrating that comorbidities may contribute more to prognosis in HFpEF patients with lower NT-proBNP levels compared to HFrEF. We also tested study-level background medical therapy, including ACEi/Angiotensin receptor blockers (ARB)/ARNI, beta-blockers, and MRAs. Uptake of these disease-modifying treatments did not significantly alter the relative difference in outcomes between HFpEF and HFrEF.

### Healthcare utilization patterns

Healthcare utilization patterns revealed interesting disparities. While HFpEF patients showed lower rates of prior HF hospital admissions (RR: 0.77) and in-hospital mortality (RR: 0.73), LoS was similar between groups. This finding is similar to the data from the ADHERE registry^22^ and suggests that acute care needs may be comparable once hospitalization occurs, despite different underlying pathophysiology and effectively worse prognosis in HFrEF.

Our findings have several important implications for clinical practice. The consistently better outcomes in HFpEF across multiple endpoints and time frames suggest that risk stratification strategies may need to be phenotype-specific. While both conditions carry substantial mortality risk, as evidenced by the high absolute event rates (112 vs 148 deaths per 1000 patient-years), the higher risk in HFrEF may warrant more intensive monitoring and earlier follow-up after discharge. However, the similar length of stay between phenotypes indicates that acute care needs to remain comparable once hospitalized, emphasizing the importance of maintaining vigilance in both conditions during acute decompensation.

### Future treatment landscape and outcome implications

The observed mortality advantage in HFpEF compared to HFrEF may further widen with recent therapeutic advances. The emergence of effective treatments for HFpEF, particularly SGLT2i, which demonstrated significant mortality and hospitalization benefits,^13,14^ represents a major shift in the historically limited therapeutic options for these patients. Moreover, given the substantial impact of comorbidities in HFpEF, advances in the management of conditions like obesity and diabetes, such as glucagon—like peptide −1 receptor agonists, which have shown CV benefits beyond glycaemic control, may further improve outcomes.^23–25^ This has been recently demonstrated in the SUMMIT trial, where tirzepatide showed a reduction of the combined endpoint of CV mortality and HF hospitalizations in HFpEF with obesity.^26^ As these therapies become more widely adopted in clinical practice, future studies may reveal a widening of the outcome differences between HF phenotypes. However, the persistent challenge of addressing the complex, multifactorial nature of HFpEF suggests that a comprehensive management approach, targeting both the cardiac dysfunction and associated comorbidities, will remain crucial.

The label ‘HFpEF’ is increasingly understood not as a single disease, but as a final common pathway for multiple, distinct pathophysiologies. These diverse aetiologies include metabolic inflammation in obesity, atrial myopathy in atrial fibrillation, and chronic cardiorenal dysfunction. All of these pathways result in the shared haemodynamic profile of elevated filling pressures.^27^ This underlying heterogeneity provides a compelling explanation for the failure of many large-scale trials that approached HFpEF as a uniform entity. Accordingly, a paradigm shift towards phenotype-directed treatment is needed. This is strongly supported by machine learning analyses identifying subgroups with unique therapeutic responses to agents like SGLT2 inhibitors, arguing for future trials that enrol these specific, more homogeneous patient cohorts.^28^

## Strengths and limitations

The strengths of this meta-analysis include its large sample size, diverse international population, and the use of adjusted analyses to account for confounding factors. Most patients in this meta-analysis were derived from registry-based studies, providing real-world data that support the external validity of our findings. However, several limitations must be acknowledged. High heterogeneity was observed in many of the pooled estimates (*I*² > 90%) alongside wide 95% PI that crossed the null, reflecting differences in study designs, patient populations, heterogeneity in endpoint definitions and varying definitions of HFpEF across studies. While we attempted to explain this heterogeneity through subgroup analyses of changing guideline-directed medical therapy, exclusion of specific HF populations, follow-up duration, and clinical settings at recruitment, substantial unexplained variance remains, possibly due to differences in adjusted models utilizing different covariates and temporal changes in HF management practices. Publication bias was evident in some outcomes, indicating that studies with positive findings may be overrepresented. We performed study-level meta-regression, including the use of ACEi/ARB/ARNI, beta-blockers, MRAs, and diuretics, and also stratified by therapeutic era. Neither medication uptake nor era meaningfully explained the difference in outcomes between HFpEF and HFrEF. This could be due to poor reporting of background medical therapy that was inconsistent across studies (e.g. only baseline therapy available) and was often absent before the drug class first emerged as beneficial. Despite numerous recent studies included, we could not assess how the SGLT2-inhibitor use moderates the difference in outcomes between phenotypes, as few studies reported their usage frequency. In addition, outcomes were rarely reported separately by race or ethnicity. We therefore did not attempt a quantitative analysis of ethnic differences to avoid drawing ecological inferences from study-level aggregates. This analysis did not include patients with HFmrEF (LVEF: 40%–49%). We prespecified HFpEF (≥50%) and HFrEF (<40%) to create two clearly separated phenotypes. Although this improves internal validity, it means that our findings apply only to HFpEF and HFrEF and should not be assumed to apply to the intermediate EF range. We also explored small-study and publication bias using Egger’s test and trim-and-fill procedures. Although trim-and-fill suggested that the differences in all-cause and CV mortality between HFpEF and HFrEF might be attenuated or no longer significant, this method assumes that missing studies are related to the magnitude of the treatment effect. In the present context, most included studies were not primarily designed to compare outcomes between HF phenotypes, making it less likely that selective non-publication is systematically driven by the HFpEF–HFrEF contrast; therefore, the trim-and-fill results should be interpreted cautiously and not viewed as definitive.

## Conclusions

This meta-analysis of over 300 000 patients demonstrates that HFpEF patients have significantly better outcomes than HFrEF patients, with lower risks of all-cause mortality, CV mortality, and HF hospitalizations. Adjusted models confirm that these differences are robust for mortality outcomes. Despite better relative outcomes, the high absolute mortality in HFpEF highlights the need for effective treatments to improve prognosis in this growing patient population.

## Supplementary Material

xvag026_Supplementary_Data

## Declarations

### Data Availability

Data are available upon request.

## References

[1] Dunlay SM, Roger VL, Redfield MM. Epidemiology of heart failure with preserved ejection fraction. Nat Rev Cardiol 2017;14:591–602. 10.1038/nrcardio.2017.65 28492288

[2] Owan TE, Hodge DO, Herges RM, Jacobsen SJ, Roger VL, Redfield MM. Trends in prevalence and outcome of heart failure with preserved ejection fraction. N Engl J Med 2006;355:251–9. 10.1056/NEJMoa052256 16855265

[3] Bhatia RS, Tu JV, Lee DS, Austin PC, Fang J, Haouzi A, et al Outcome of heart failure with preserved ejection fraction in a population-based study. N Engl J Med 2006;355:260–9. 10.1056/NEJMoa051530 16855266

[4] Meta-analysis Global Group in Chronic Heart Failure (MAGGIC) . The survival of patients with heart failure with preserved or reduced left ventricular ejection fraction: an individual patient data meta-analysis. Eur Heart J 2012;33:1750–7. 10.1093/eurheartj/ehr254 21821849

[5] Lam CSP, Gamble GD, Ling LH, Sim D, Leong KTG, Yeo PSD, et al Mortality associated with heart failure with preserved vs. reduced ejection fraction in a prospective international multi-ethnic cohort study. Eur Heart J 2018;39:1770–80. 10.1093/eurheartj/ehy005 29390051

[6] Shah KS, Xu H, Matsouaka RA, Bhatt DL, Heidenreich PA, Hernandez AF, et al Heart failure with preserved, borderline, and reduced ejection fraction. J Am Coll Cardiol 2017;70:2476–86. 10.1016/j.jacc.2017.08.074 29141781

[7] Tsao CW, Lyass A, Enserro D, Larson MG, Ho JE, Kizer JR, et al Temporal trends in the incidence of and mortality associated with heart failure with preserved and reduced ejection fraction. JACC Heart Fail 2018;6:678–85. 10.1016/j.jchf.2018.03.006 30007560 PMC6076350

[8] Page MJ, McKenzie JE, Bossuyt PM, Boutron I, Hoffmann TC, Mulrow CD, et al The PRISMA 2020 statement: an updated guideline for reporting systematic reviews. BMJ 2021;372:n71. 10.1136/bmj.n71 33782057 PMC8005924

[9] Luchini C, Stubbs B, Solmi M, Veronese N. Assessing the quality of studies in meta-analyses: advantages and limitations of the Newcastle Ottawa Scale. World J Metaanal 2017;5:80–4. 10.13105/wjma.v5.i4.80

[10] Wan X, Wang W, Liu J, Tong T. Estimating the sample mean and standard deviation from the sample size, median, range and/or interquartile range. BMC Med Res Methodol 2014;14:135. 10.1186/1471-2288-14-135 25524443 PMC4383202

[11] Egger M, Davey Smith G, Schneider M, Minder C. Bias in meta-analysis detected by a simple, graphical test. BMJ 1997;315:629–34. 10.1136/bmj.315.7109.629 9310563 PMC2127453

[12] Wang H, Li Y, Chai K, Long Z, Yang Z, Du M, et al Mortality in patients admitted to hospital with heart failure in China: a nationwide Cardiovascular Association Database-Heart Failure Centre Registry cohort study. Lancet Glob Health 2024;12:e611–22. 10.1016/S2214-109X(23)00605-8 38485428

[13] Solomon SD, McMurray JJV, Claggett B, de Boer RA, DeMets D, Hernandez AF, et al Dapagliflozin in heart failure with mildly reduced or preserved ejection fraction. N Engl J Med 2022;387:1089–98. 10.1056/NEJMoa2206286 36027570

[14] Anker SD, Butler J, Filippatos G, Ferreira JP, Bocchi E, Böhm M, et al Empagliflozin in heart failure with a preserved ejection fraction. N Engl J Med 2021;385:1451–61. 10.1056/NEJMoa2107038 34449189

[15] Tromp J, Ouwerkerk W, van Veldhuisen VD, Hillege HL, Richards AM, van der Meer P, et al A systematic review and network meta-analysis of pharmacological treatment of heart failure with reduced ejection fraction. JACC Heart Fail 2022;10:73–84. 10.1016/j.jchf.2021.09.004 34895860

[16] Schmidt M, Ulrichsen SP, Pedersen L, Bøtker HE, Sørensen HT. Thirty-year trends in heart failure hospitalization and mortality rates and the prognostic impact of co-morbidity: a Danish nationwide cohort study. Eur J Heart Fail 2016;18:490–9. 10.1002/ejhf.486 26868921

[17] Danielsen R, Thorgeirsson G, Einarsson H, Ólafsson Ö, Aspelund T, Harris TB, et al Prevalence of heart failure in the elderly and future projections: the AGES-Reykjavík study. Scand Cardiovasc J 2017;51:183–9. 10.1080/14017431.2017.1311023 28366010 PMC5681737

[18] Kosyakovsky LB, Liu EE, Wang JK, Myers L, Parekh JK, Knauss H, et al Uncovering unrecognized heart failure with preserved ejection fraction among individuals with obesity and dyspnea. Circ Heart Fail 2024;17:e011366. 10.1161/CIRCHEARTFAILURE.123.011366 38742409 PMC11214582

[19] Zinman B, Wanner C, Lachin JM, Fitchett D, Bluhmki E, Hantel S, et al Empagliflozin, cardiovascular outcomes, and mortality in type 2 diabetes. N Engl J Med 2015;373:2117–28. 10.1056/NEJMoa1504720 26378978

[20] Wilding JPH, Batterham RL, Calanna S, Davies M, Van Gaal LF, Lingvay I, et al Once-weekly semaglutide in adults with overweight or obesity. N Engl J Med 2021;384:989–1002. 10.1056/NEJMoa2032183 33567185

[21] Salah K, Stienen S, Pinto YM, Eurlings LW, Metra M, Bayes-Genis A, et al Prognosis and NT-proBNP in heart failure patients with preserved versus reduced ejection fraction. Heart 2019;105:1182–9. 10.1136/heartjnl-2018-314173 30962192 PMC6662953

[22] Yancy CW, Lopatin M, Stevenson LW, De Marco T, Fonarow GC; ADHERE Scientific Advisory Committee and Investigators. Clinical presentation, management, and in-hospital outcomes of patients admitted with acute decompensated heart failure with preserved systolic function: a report from the Acute Decompensated Heart Failure National Registry (ADHERE) Database. J Am Coll Cardiol 2006;47:76–84. 10.1016/j.jacc.2005.09.022 16386668

[23] Marso SP, Bain SC, Consoli A, Eliaschewitz FG, Jódar E, Leiter LA, et al Semaglutide and cardiovascular outcomes in patients with type 2 diabetes. N Engl J Med 2016;375:1834–44. 10.1056/NEJMoa1607141 27633186

[24] Marso SP, Daniels GH, Brown-Frandsen K, Kristensen P, Mann JFE, Nauck MA, et al Liraglutide and cardiovascular outcomes in type 2 diabetes. N Engl J Med 2016;375:311–22. 10.1056/NEJMoa1603827 27295427 PMC4985288

[25] Gerstein HC, Colhoun HM, Dagenais GR, Diaz R, Lakshmanan M, Pais P, et al Dulaglutide and cardiovascular outcomes in type 2 diabetes (REWIND): a double-blind, randomised placebo-controlled trial. Lancet 2019;394:121–30. 10.1016/S0140-6736(19)31149-3 31189511

[26] Packer M, Zile MR, Kramer CM, Baum SJ, Litwin SE, Menon V, et al Tirzepatide for heart failure with preserved ejection fraction and obesity. N Engl J Med 2024;392:427–37. 10.1056/NEJMoa2410027 39555826

[27] Borlaug BA, Sharma K, Shah SJ, Ho JE. Heart failure with preserved ejection fraction. J Am Coll Cardiol 2023;81:1810–34. 10.1016/j.jacc.2023.01.049 37137592

[28] Li R, Liu Y, Zhao Z, Zhang C, Dong W, Qi Y, et al Machine learning-based phenotyping and assessment of treatment responses in heart failure with preserved ejection fraction. EClinicalMedicine 2025;88:103462. 10.1016/j.eclinm.2025.103462 41181830 PMC12572816

