# Supplementary material for: Mortality and heart failure hospitalizations in heart failure with preserved ejection fraction compared to heart failure with reduced ejection fraction: a systematic review and meta-analysis
Source: ESC Heart Fail. 2026 Jan 16;13(1):xvag026. doi: 10.1093/eschf/xvag026 (PMC13108283; doi:10.1093/eschf/xvag026)

**Supplementary material**

**Mortality and HF Hospitalizations in HFpEF Compared to HFrEF: A Systematic Review and Meta-Analysis**

**Figure S1.** Funnel plot for all-cause mortality
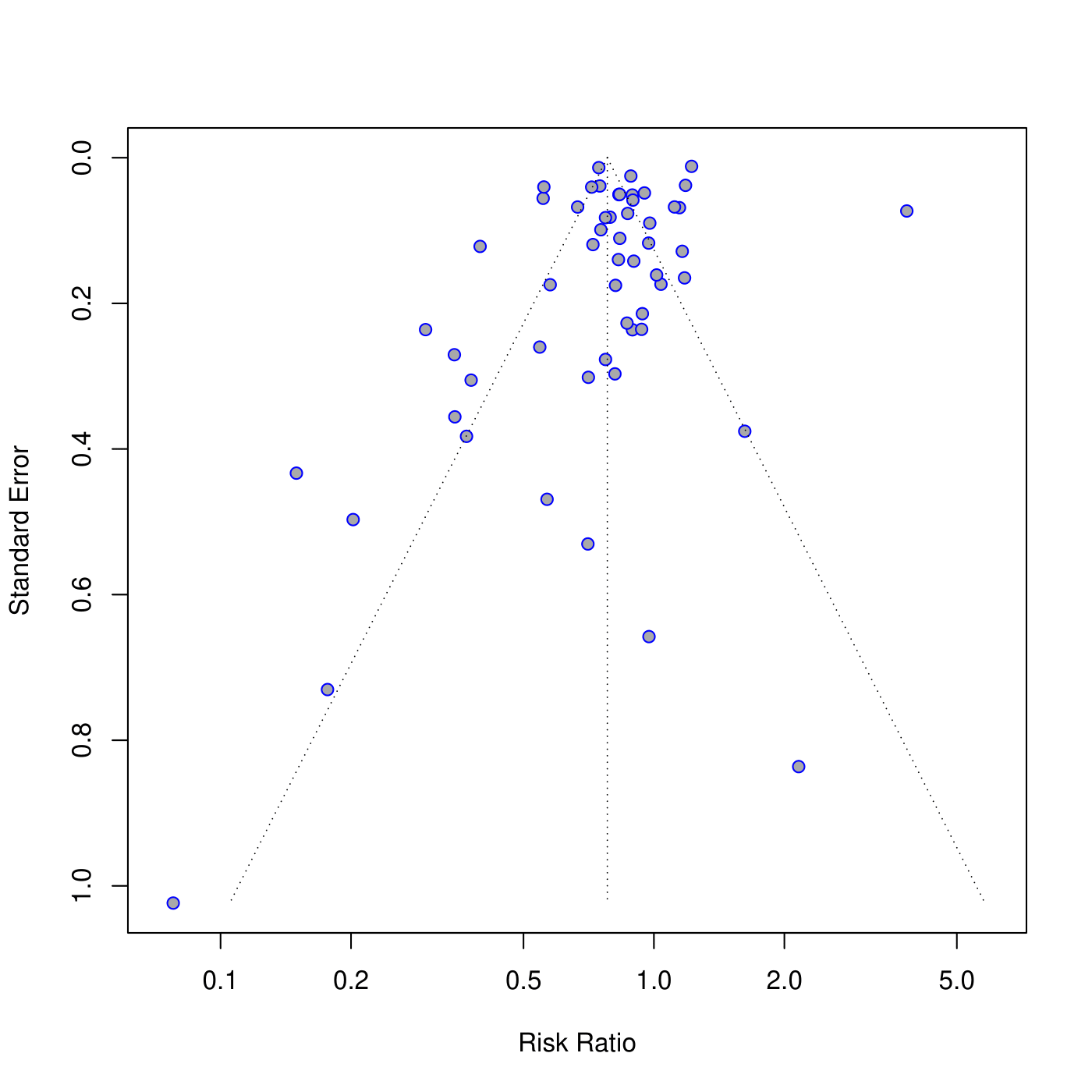


**Figure S2.** All-cause mortality results after excluding studies with high risk of bias **
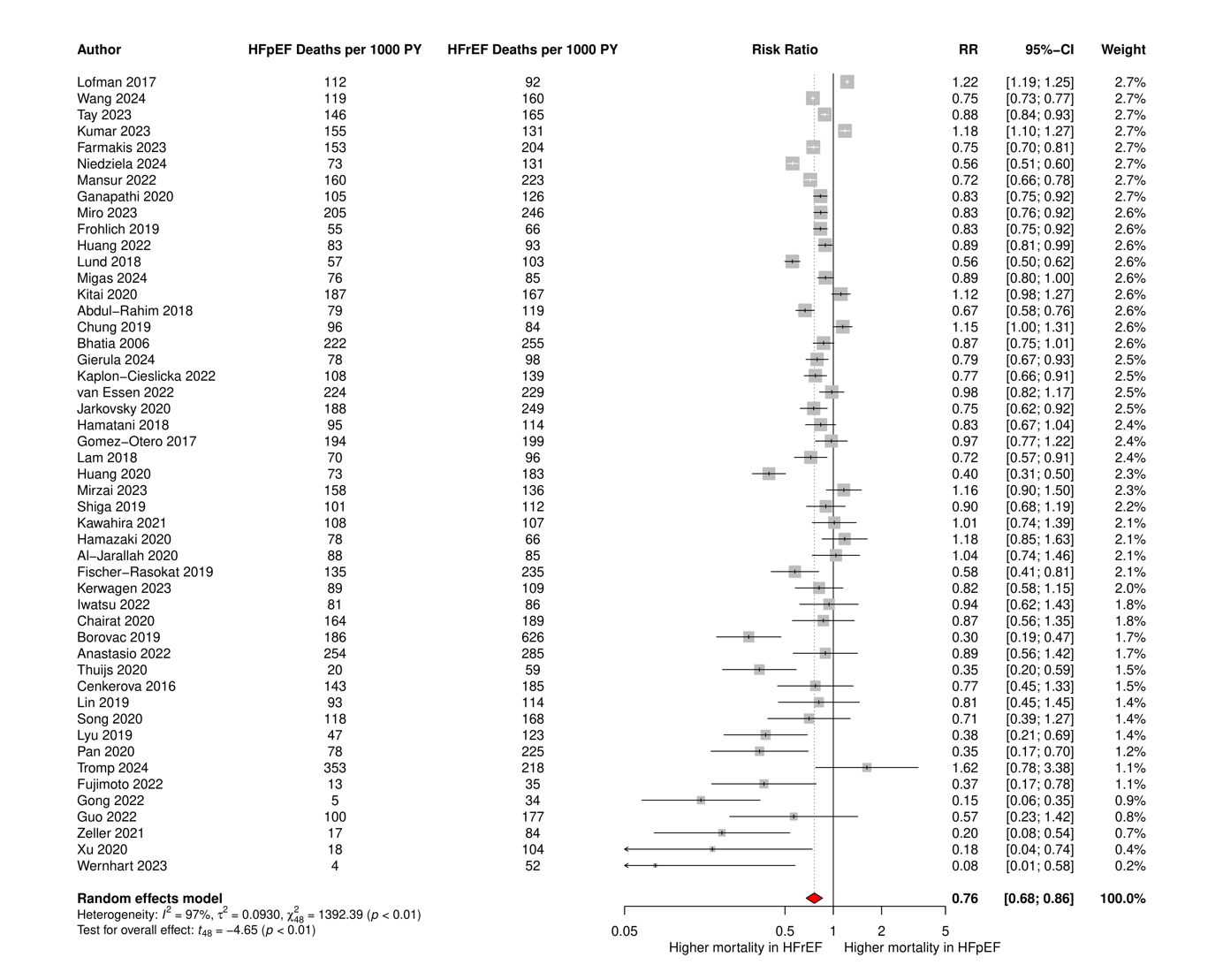
**

**Figure S3.** Funnel plot for maximally adjusted hazard ratios for all-cause mortality
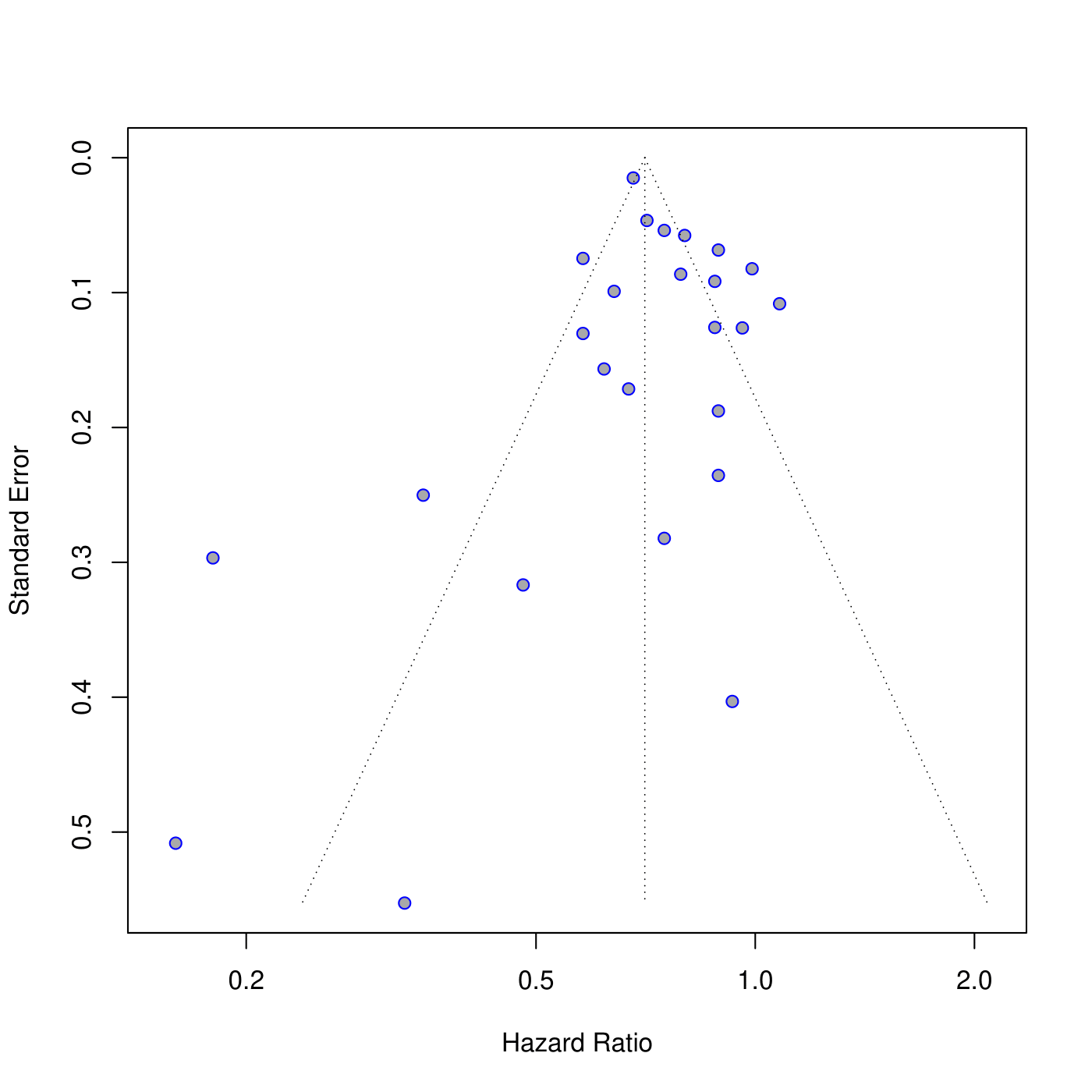


**Figure S4.** Subgroup analysis of all-cause mortality by follow-up duration
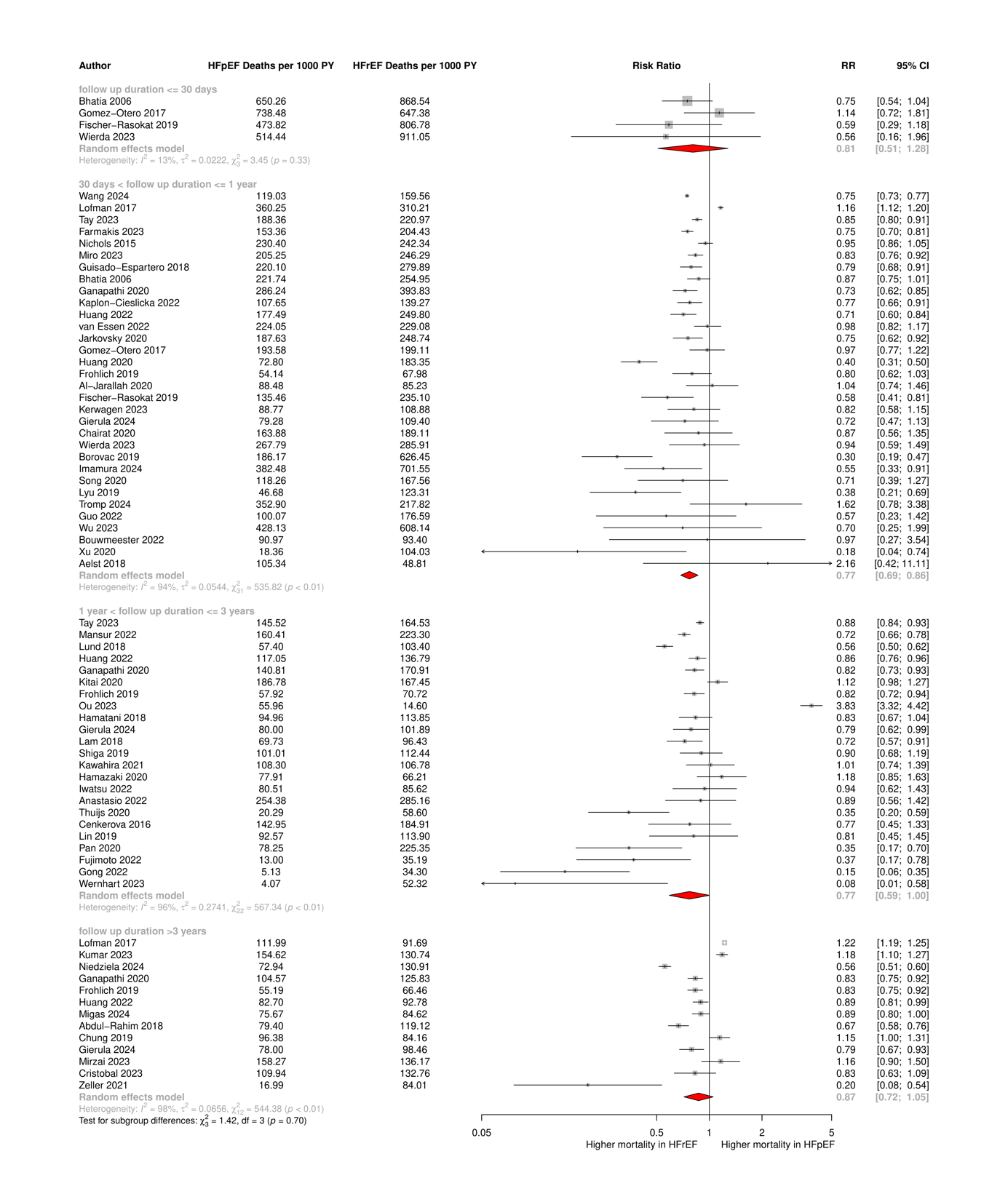


**Figure S5.** Subgroup analysis of all-cause mortality by clinical setting (inpatient and outpatient)


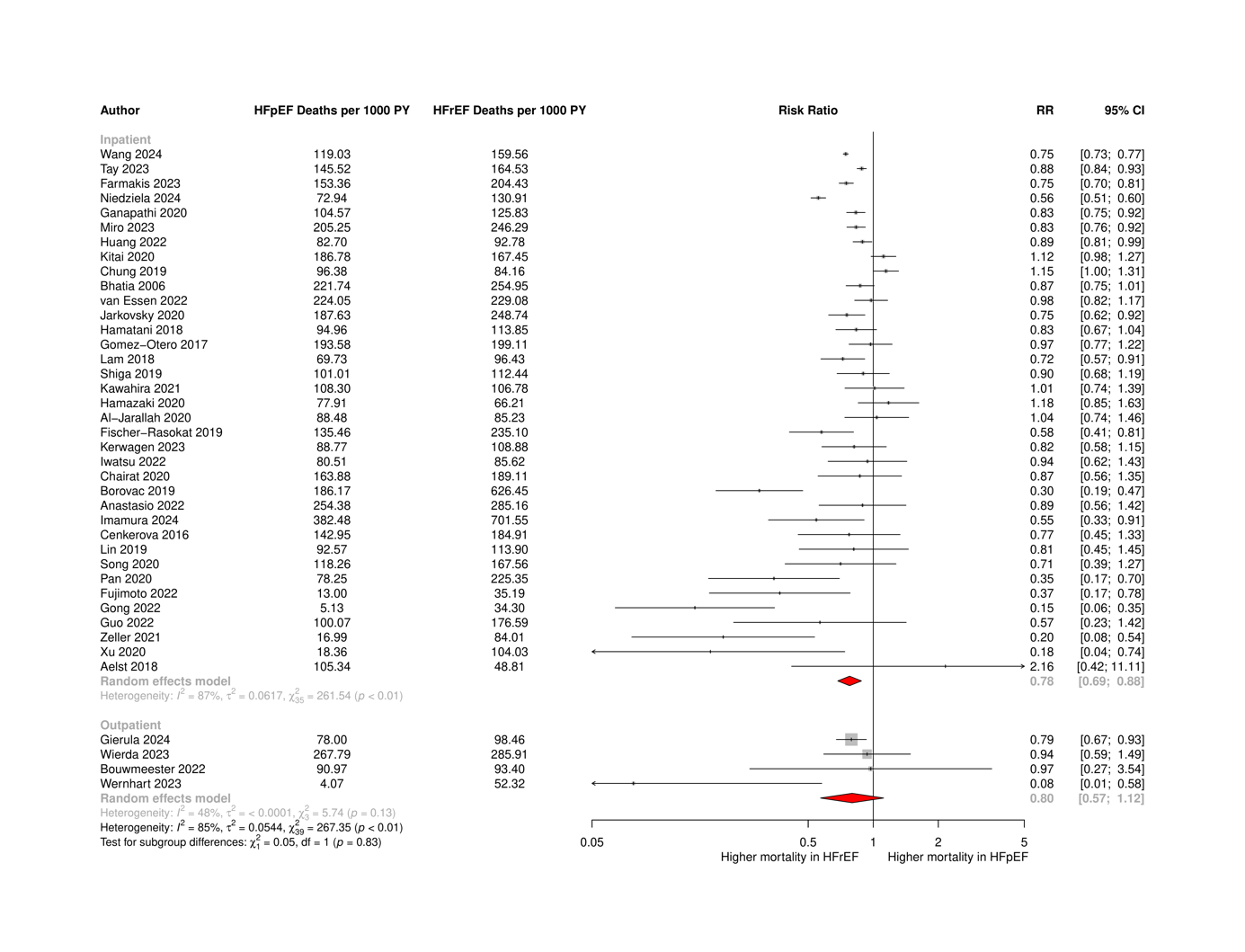


**Figure S6.** All-cause results restricted to general HF cohorts (excluding device-, procedure- or disease-specific studies)
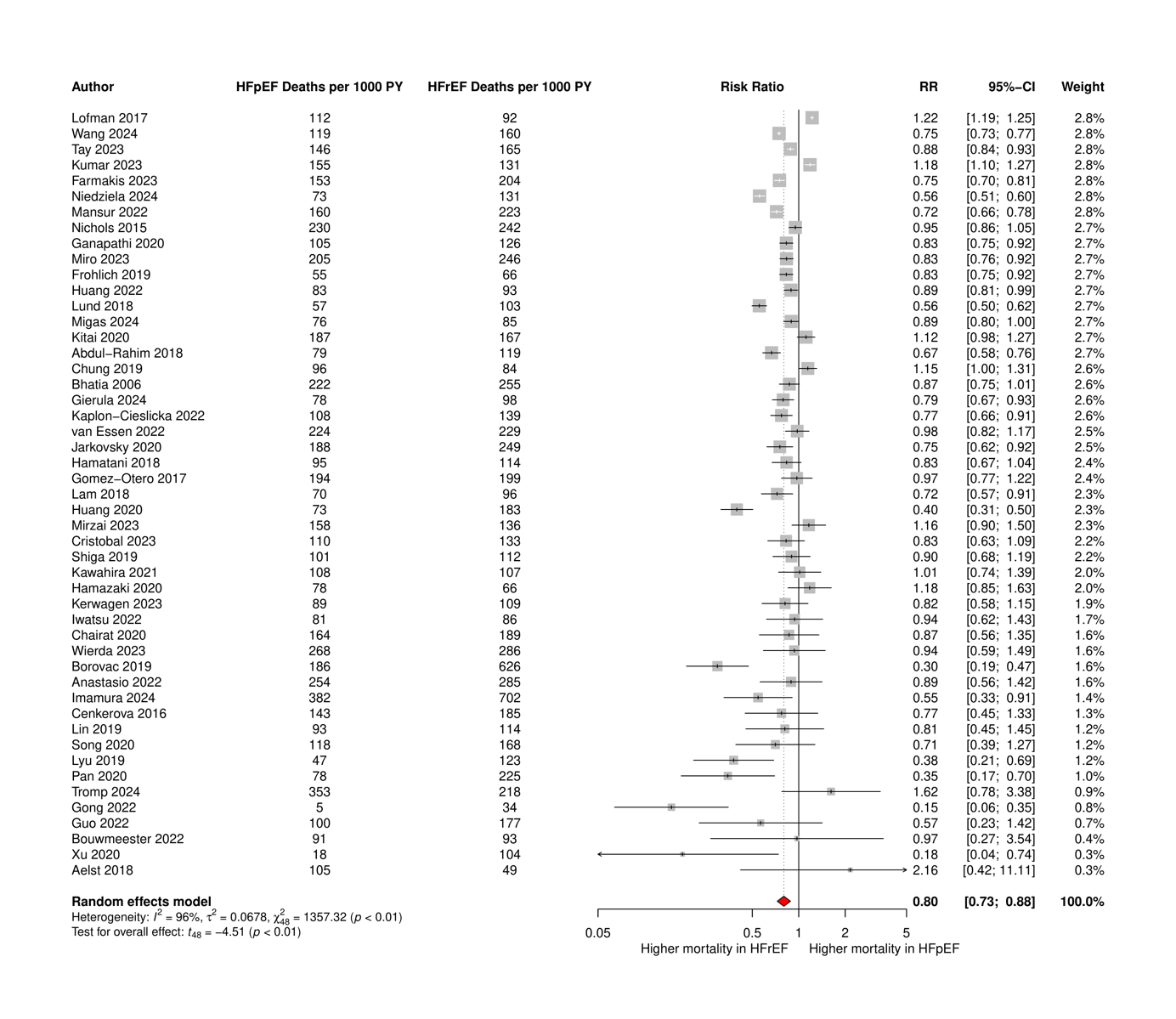


**Figure S7.** Subgroup analysis of all-cause mortality by HF presentation at enrollment (acute decompensated vs. chronic HF)
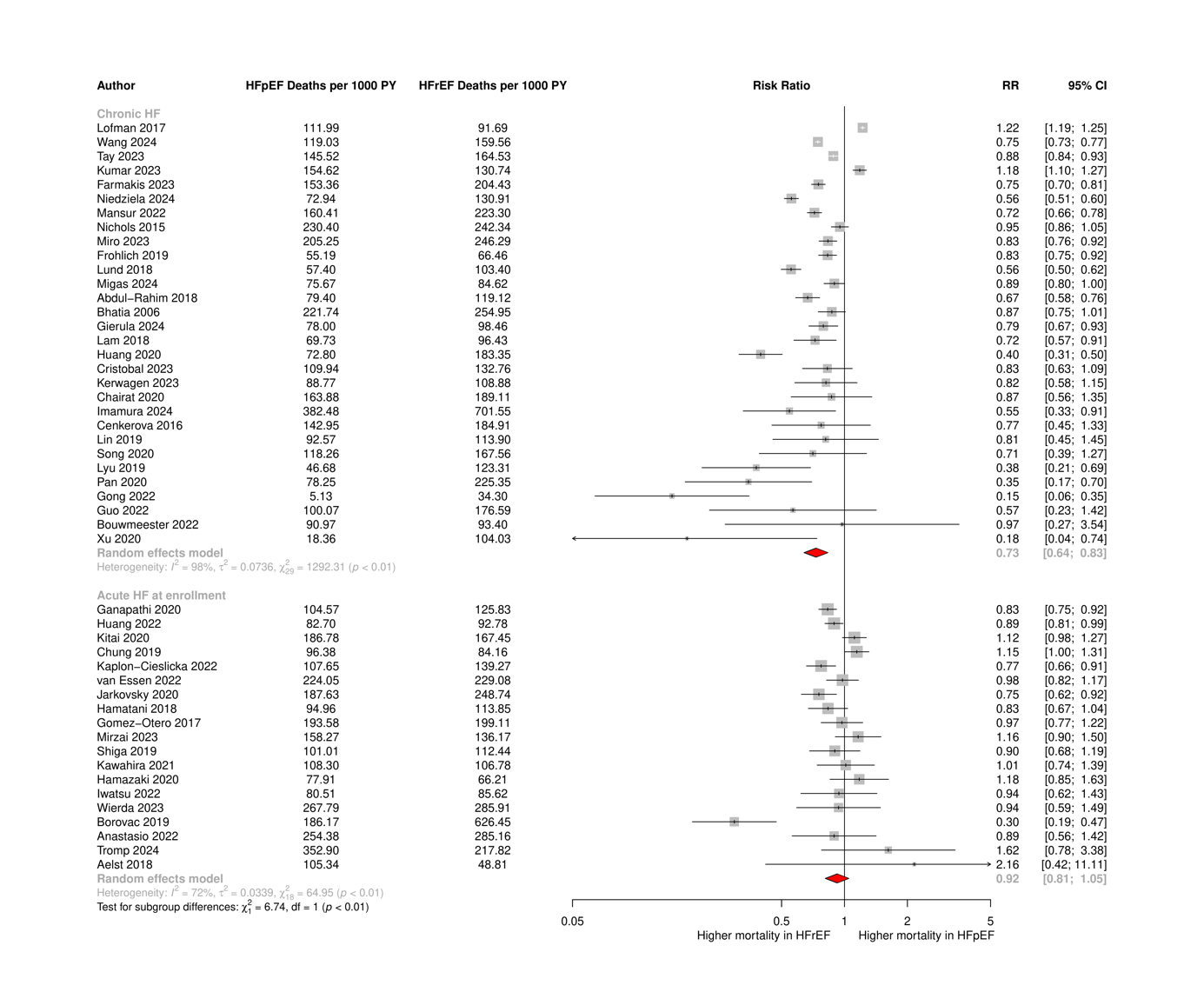


**Figure S8.** Subgroup analysis of all-cause mortality across therapeutic eras
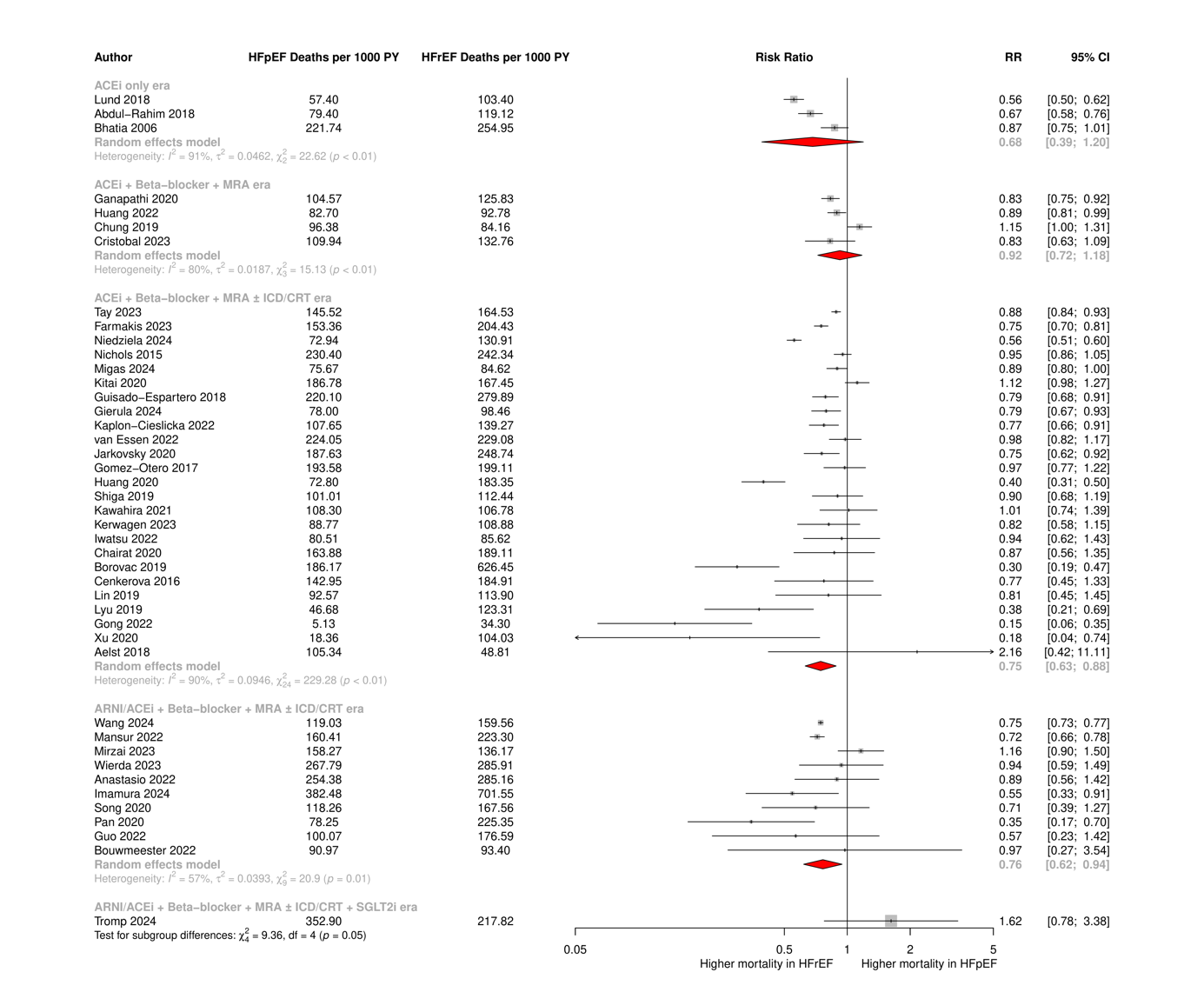


**Figure S9.** Funnel plot for cardiovascular mortality
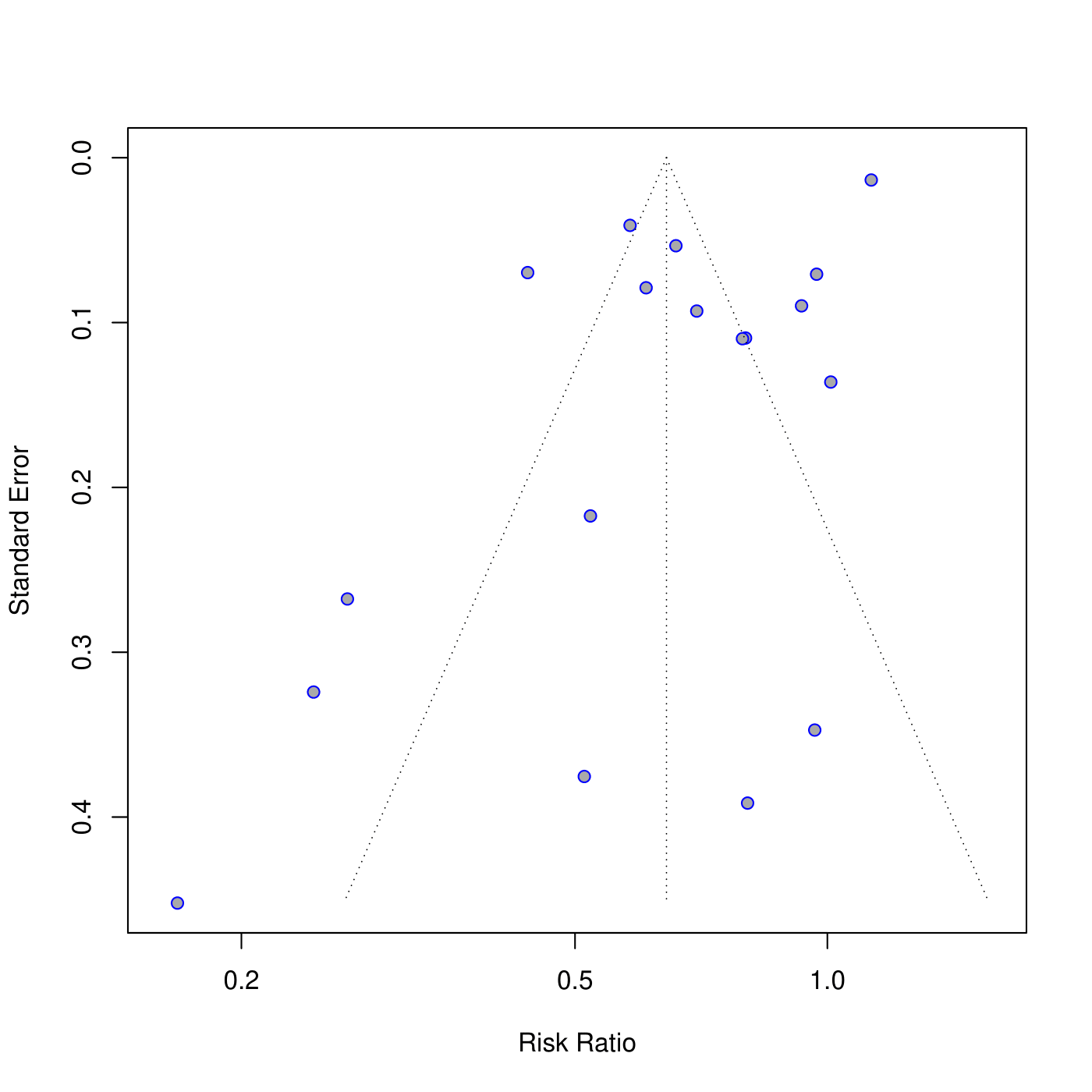


**Figure S10.** Subgroup analysis of cardiovascular mortality by follow-up duration
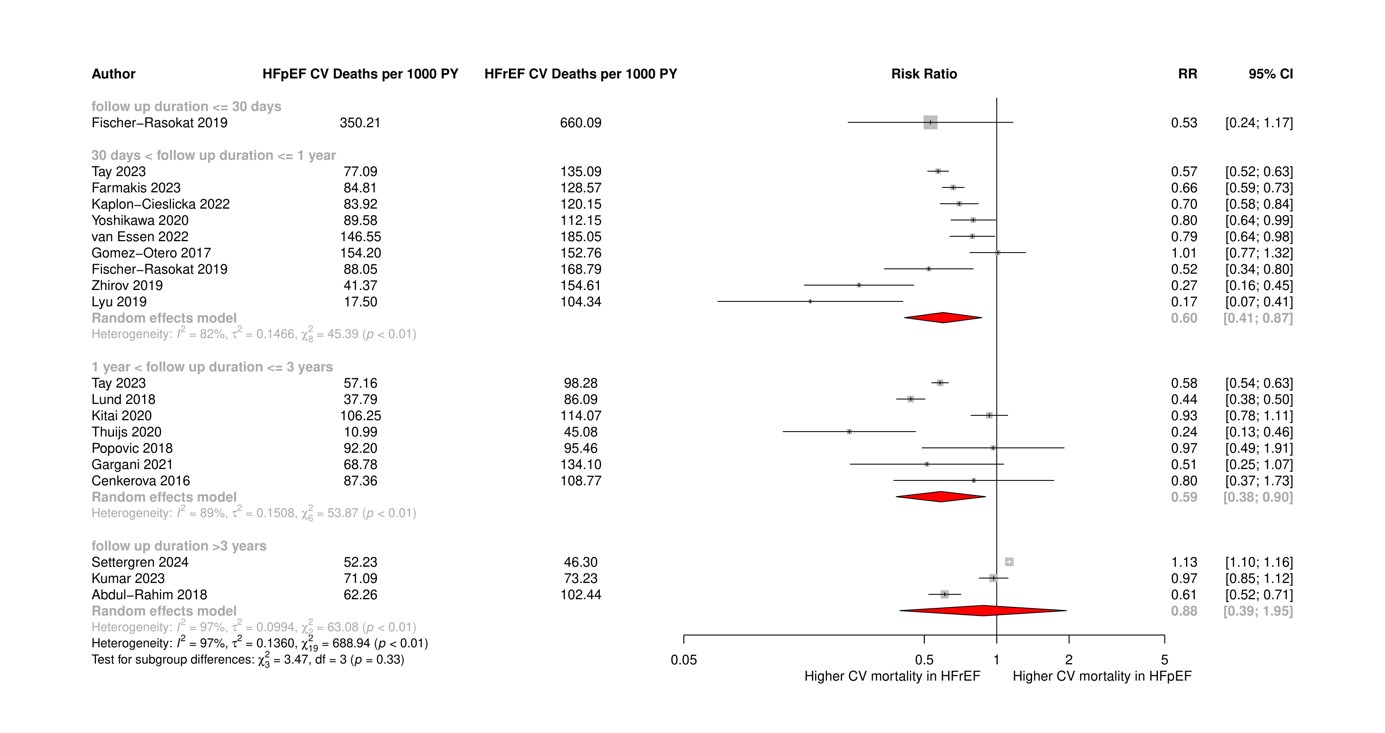


**Figure S11.** Cardiovascular mortality results after excluding studies with high risk of bias **
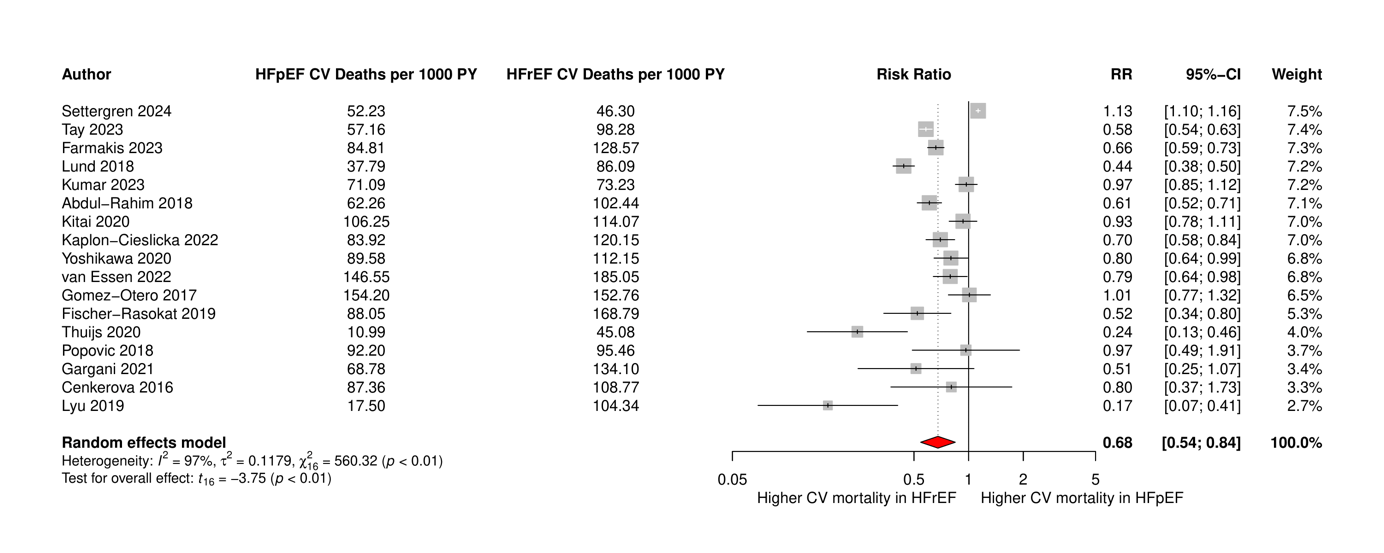
**

**Figure S12.** Cardiovascular mortality results restricted to general HF cohorts (excluding device-, procedure- or disease-specific studies)
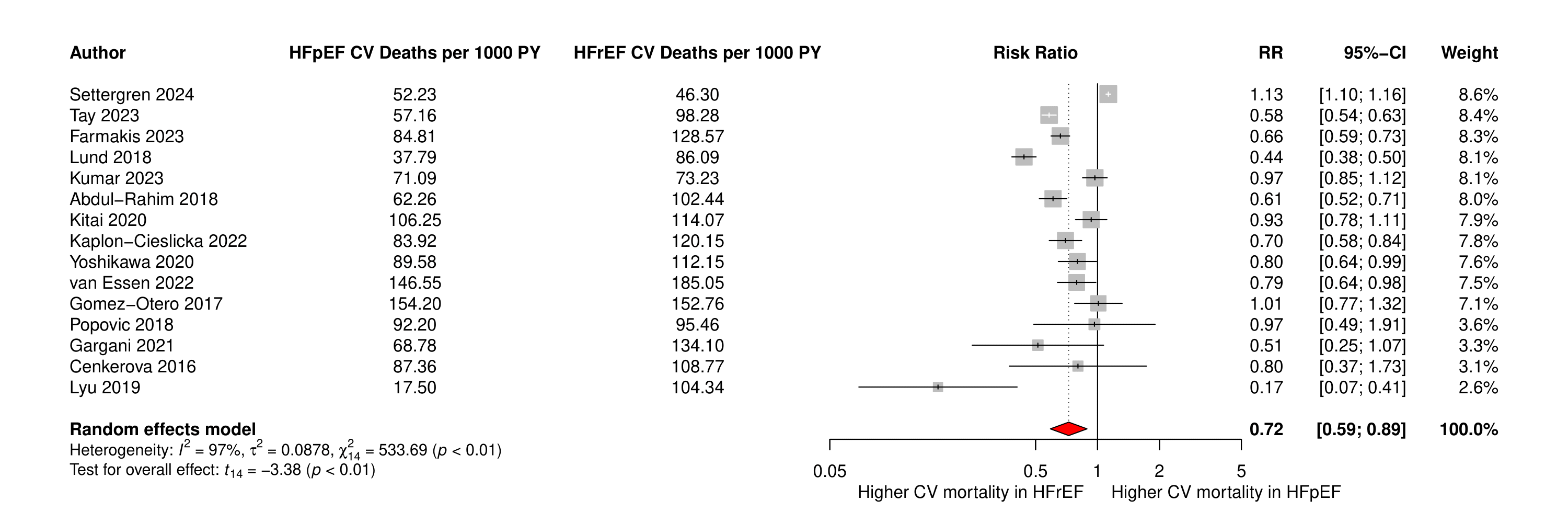


**Figure S13.** Subgroup analysis of cardiovascular mortality by HF presentation at enrollment (acute decompensated vs. chronic HF)
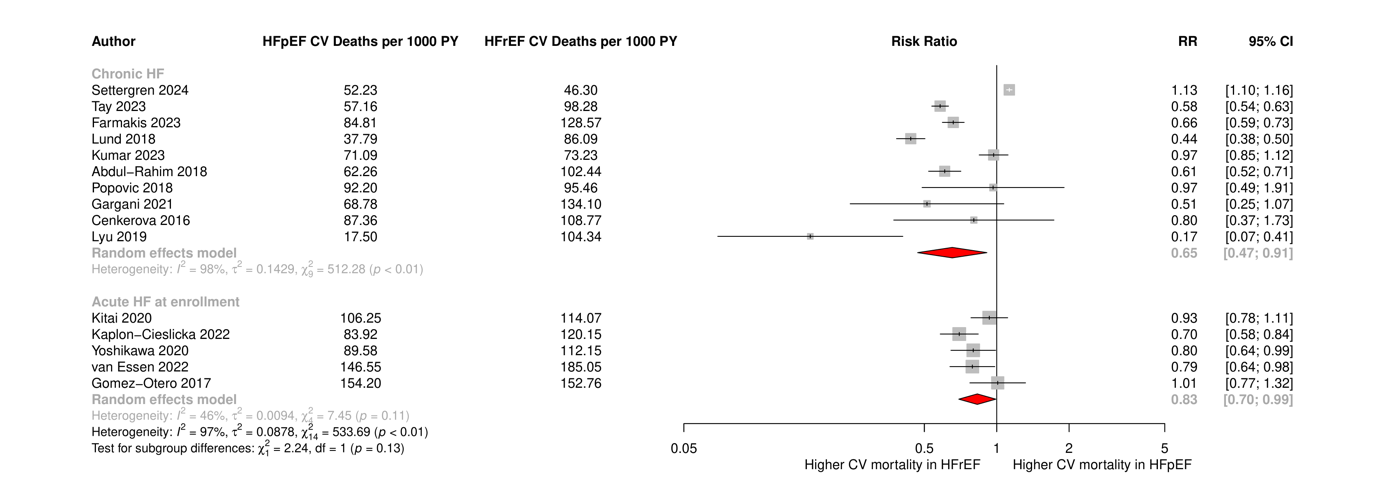


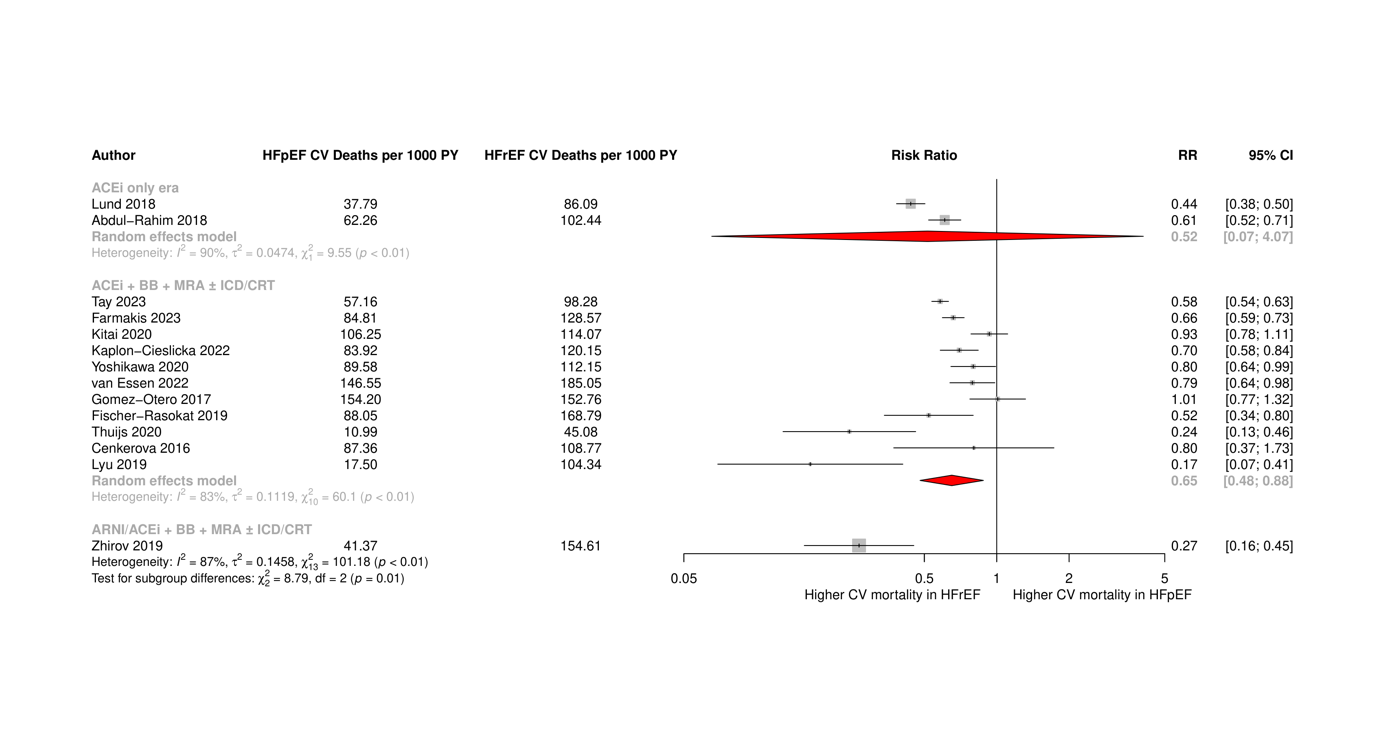
**Figure S14.** Subgroup analysis of cardiovascular mortality across therapeutic eras

**Figure S15.** Funnel plot for HF hospitalizations
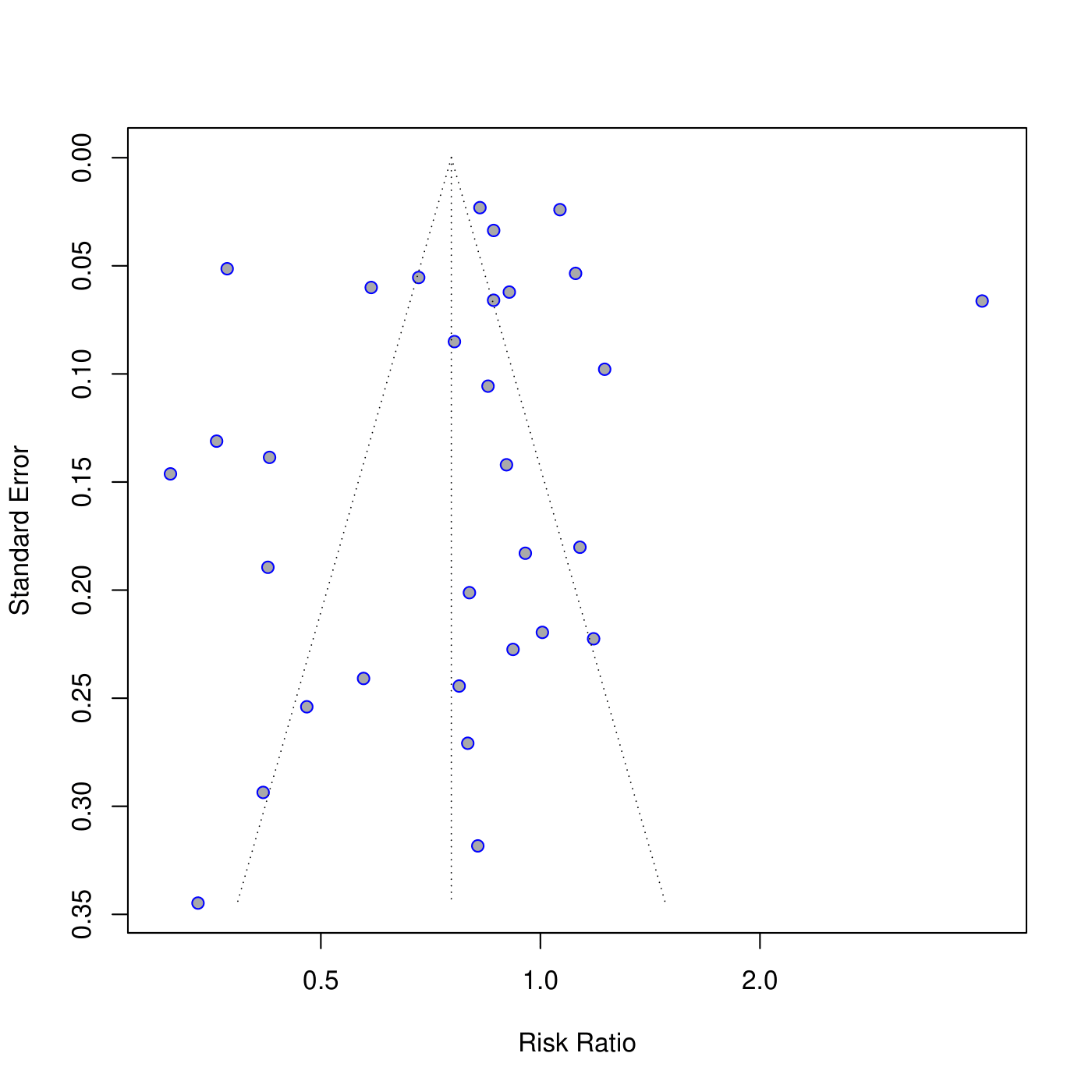


**Figure S16.** Subgroup analysis of HF hospitalizations by follow-up duration
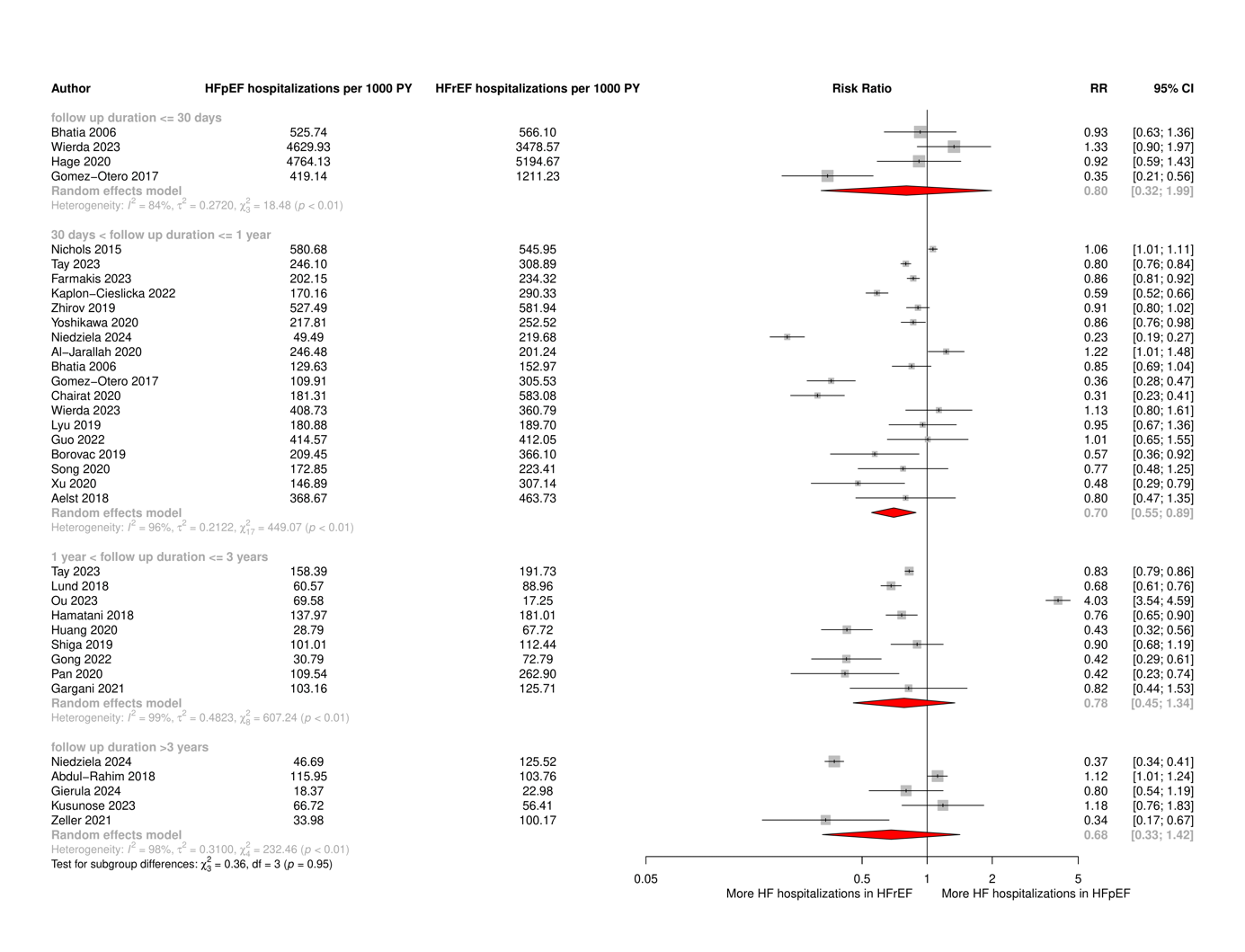


**Figure S17.** Subgroup analysis of HF hospitalizations by clinical setting (inpatient and outpatient)**
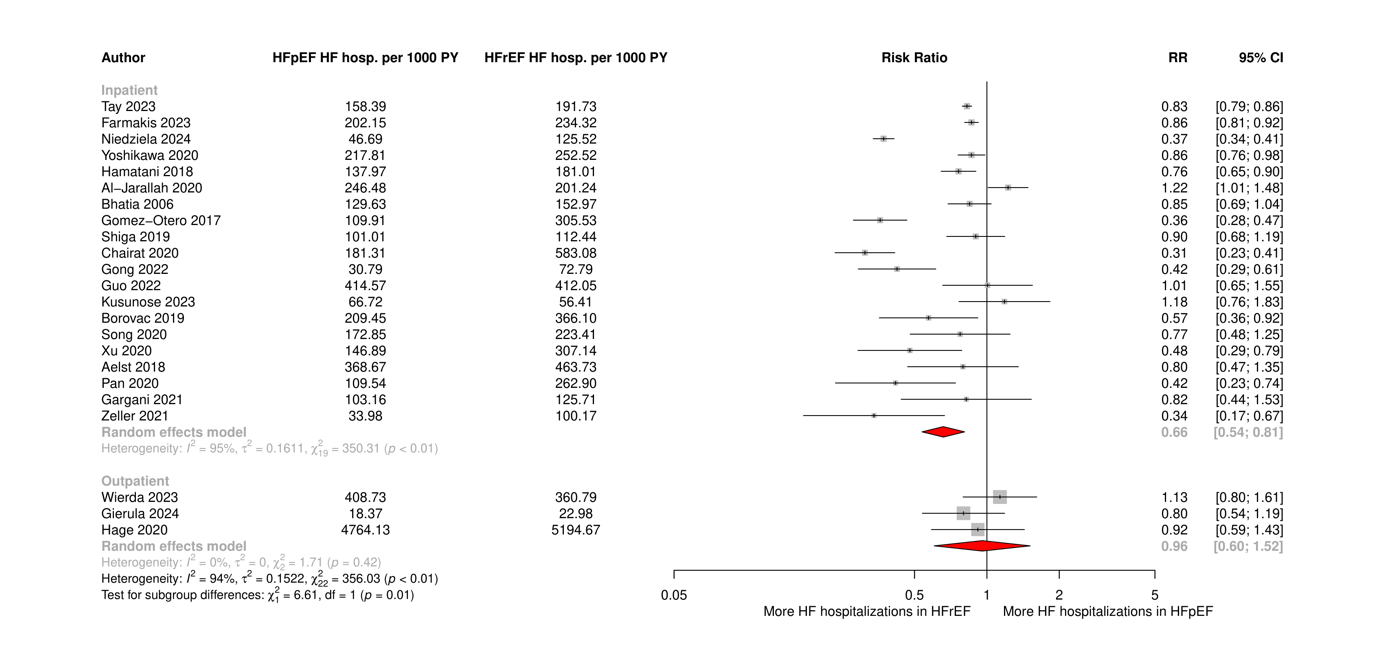
**


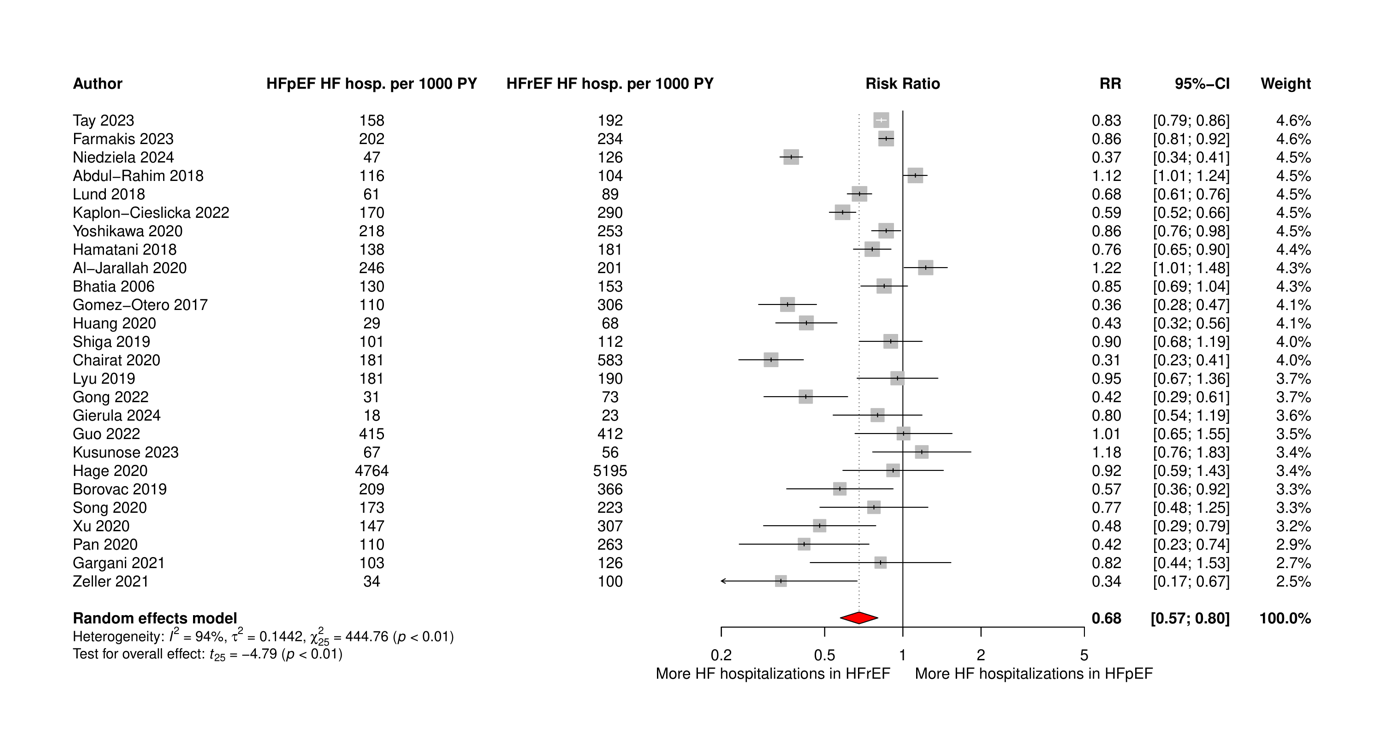
**Figure S18.** HF hospitalization results after excluding studies with high risk of bias

**Figure S19.** HF hospitalization results restricted to general HF cohorts (excluding device-, procedure- or disease-specific studies)
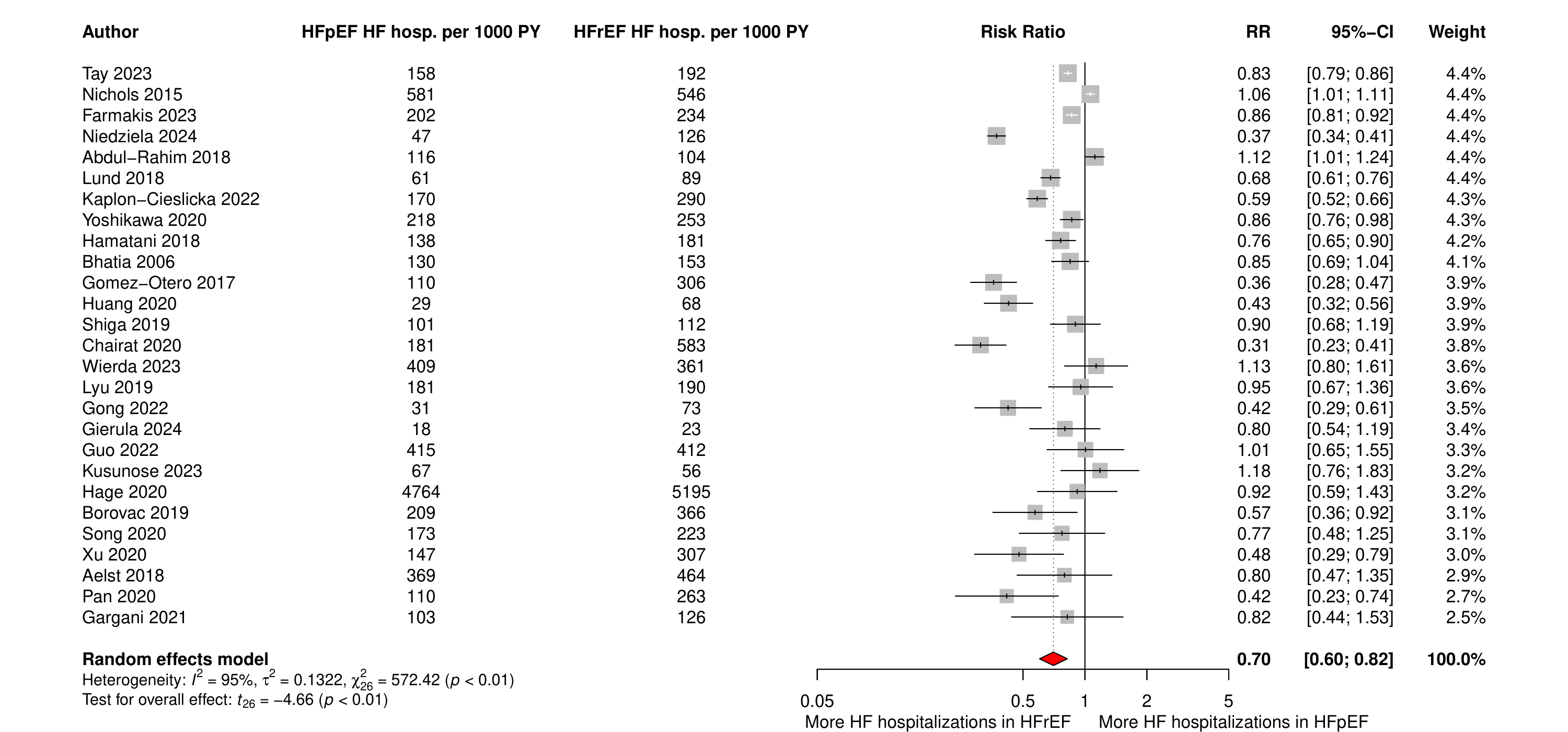


**Figure S20.** Subgroup analysis of HF hospitalizations by HF presentation at enrollment (acute decompensated vs. chronic HF)
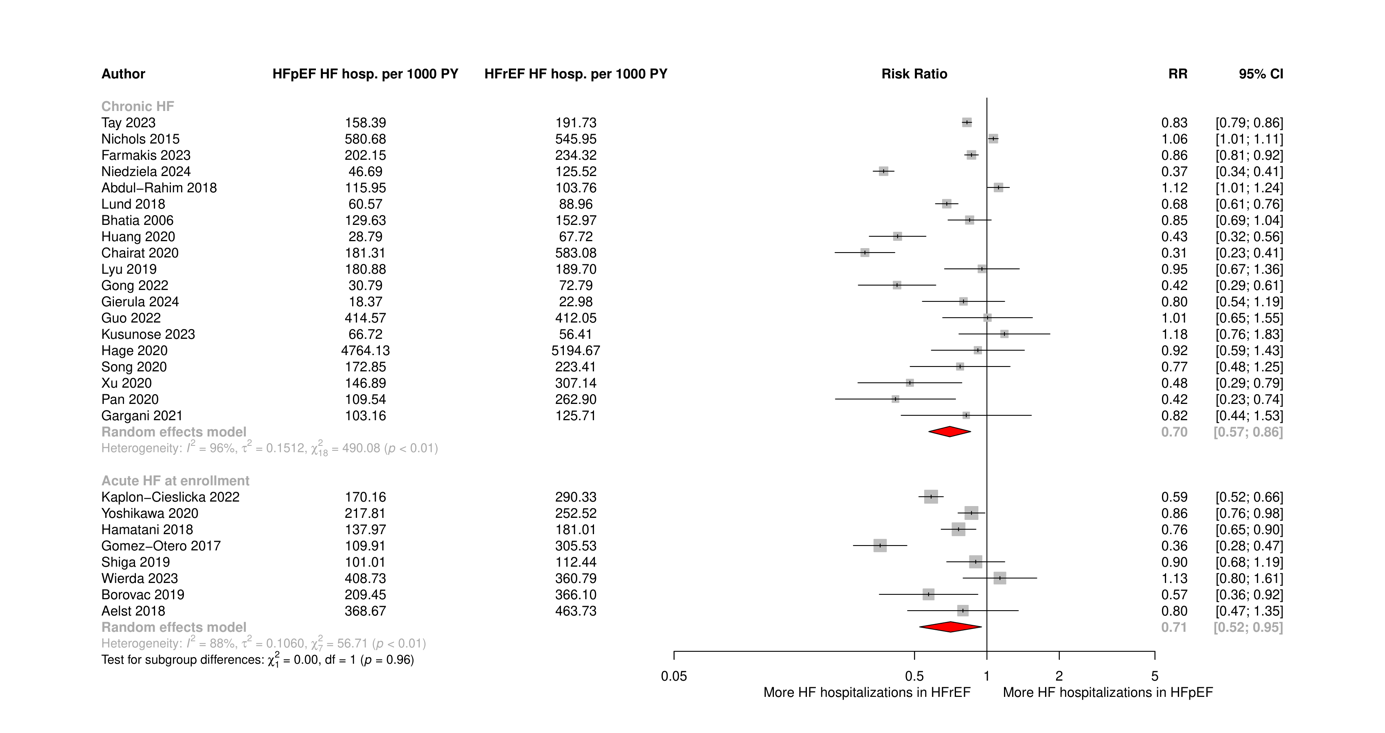


**
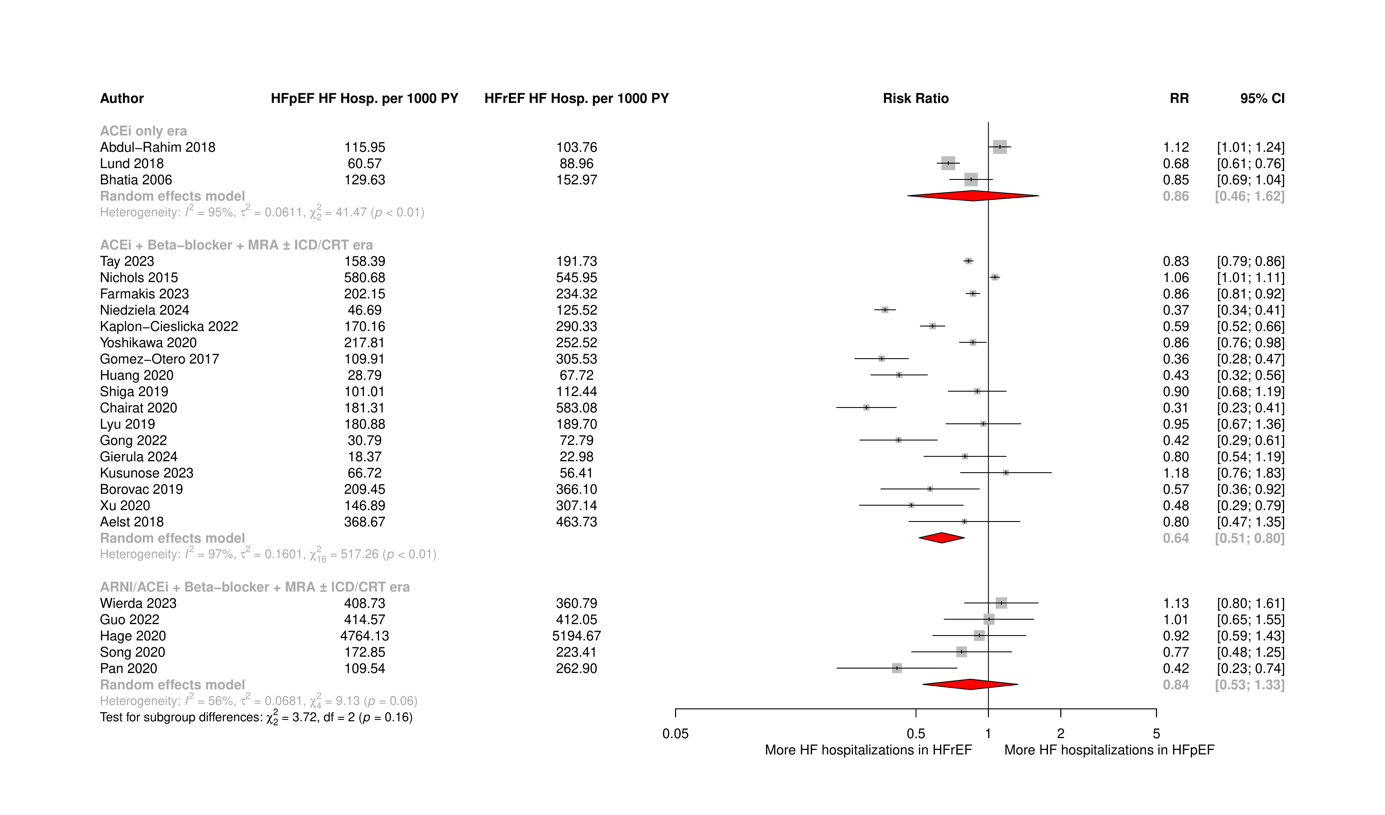
Figure S21.** Subgroup analysis of HF hospitalizations across therapeutic eras (no studies with SGLT2i yet)


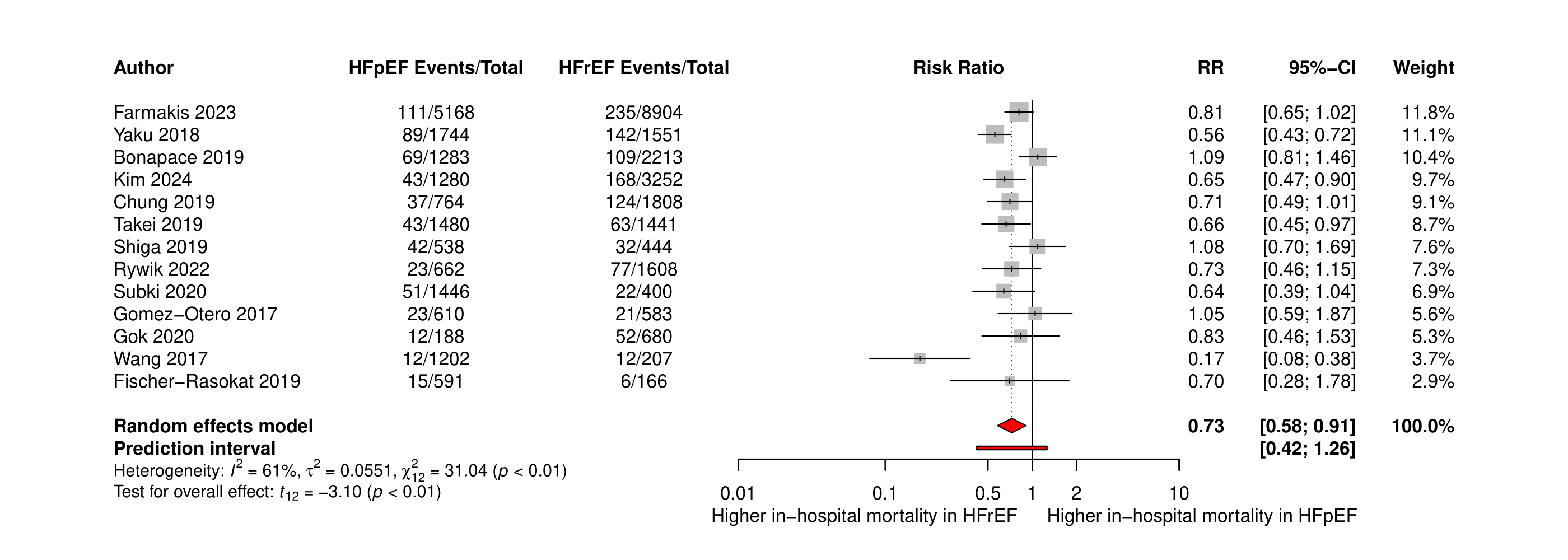
**Figure S22.** Pooled relative risk of in-hospital mortality in HFpEF compared to HFrEF

**Figure S23.** Funnel plot for in-hospital mortality
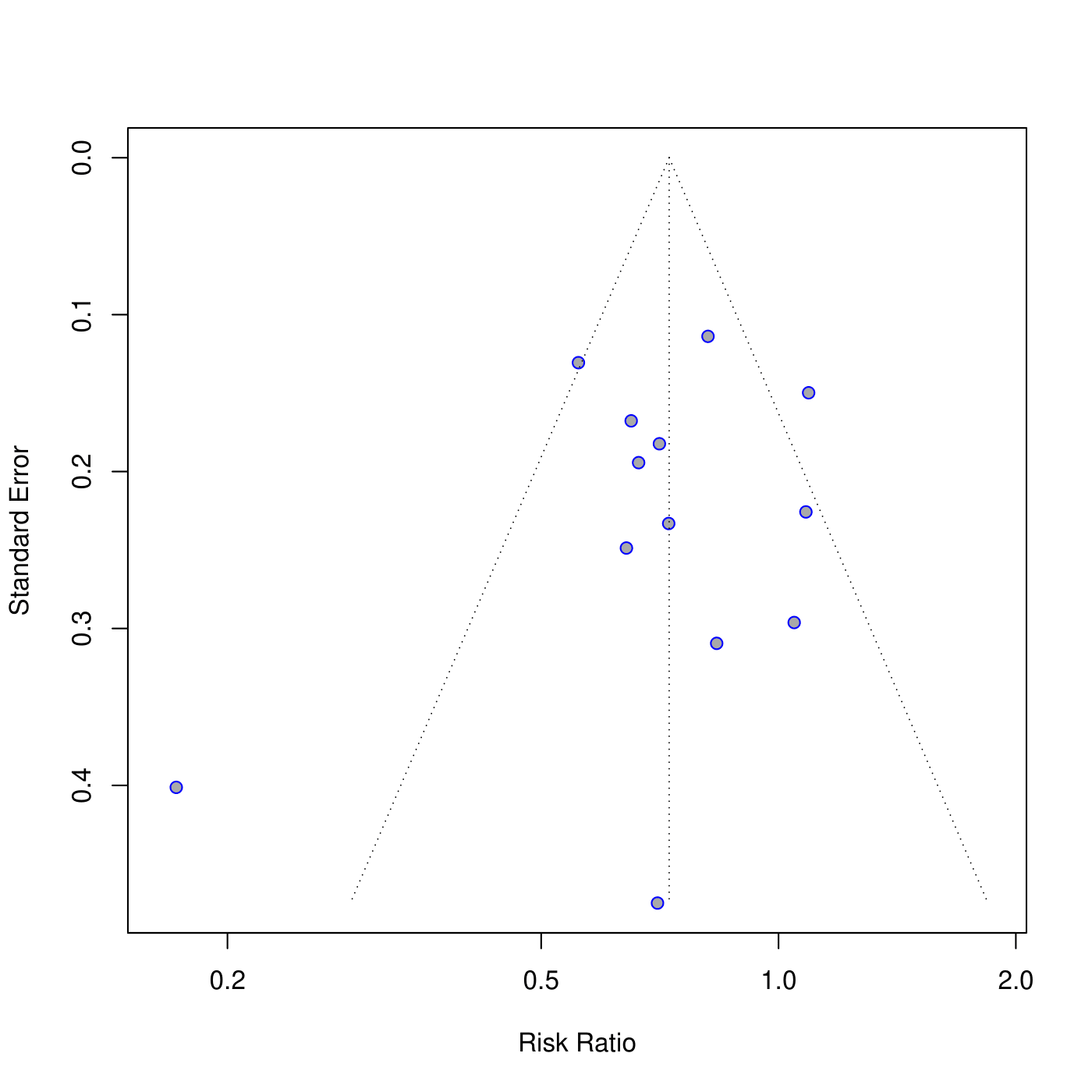


**Figure S24.** In-hospital mortality results after excluding studies with high risk of bias
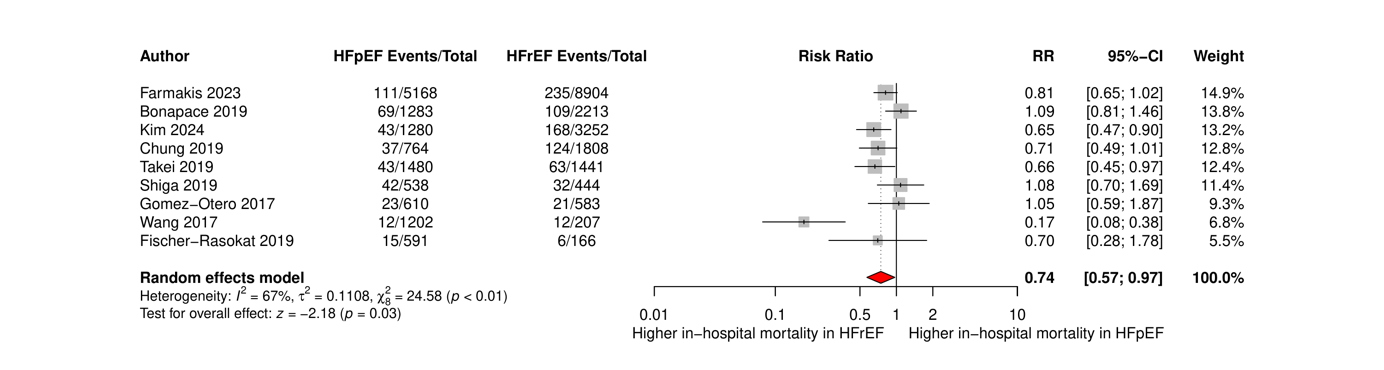


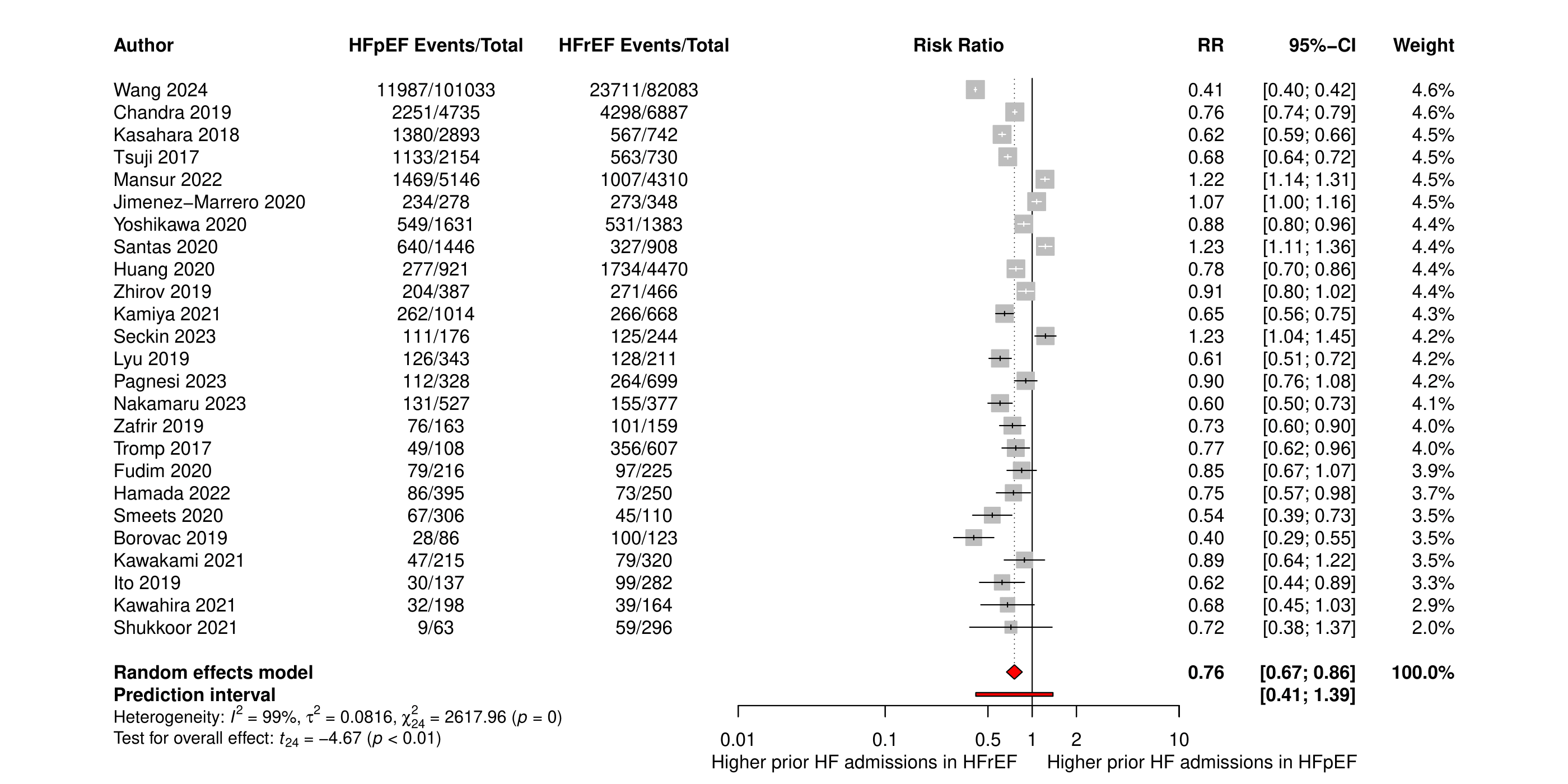
**Figure S25.** Pooled relative risk of prior HF hospital admissions in HFpEF compared to HFrEF

**Figure S26.** Funnel plot for prior HF hospital admissions
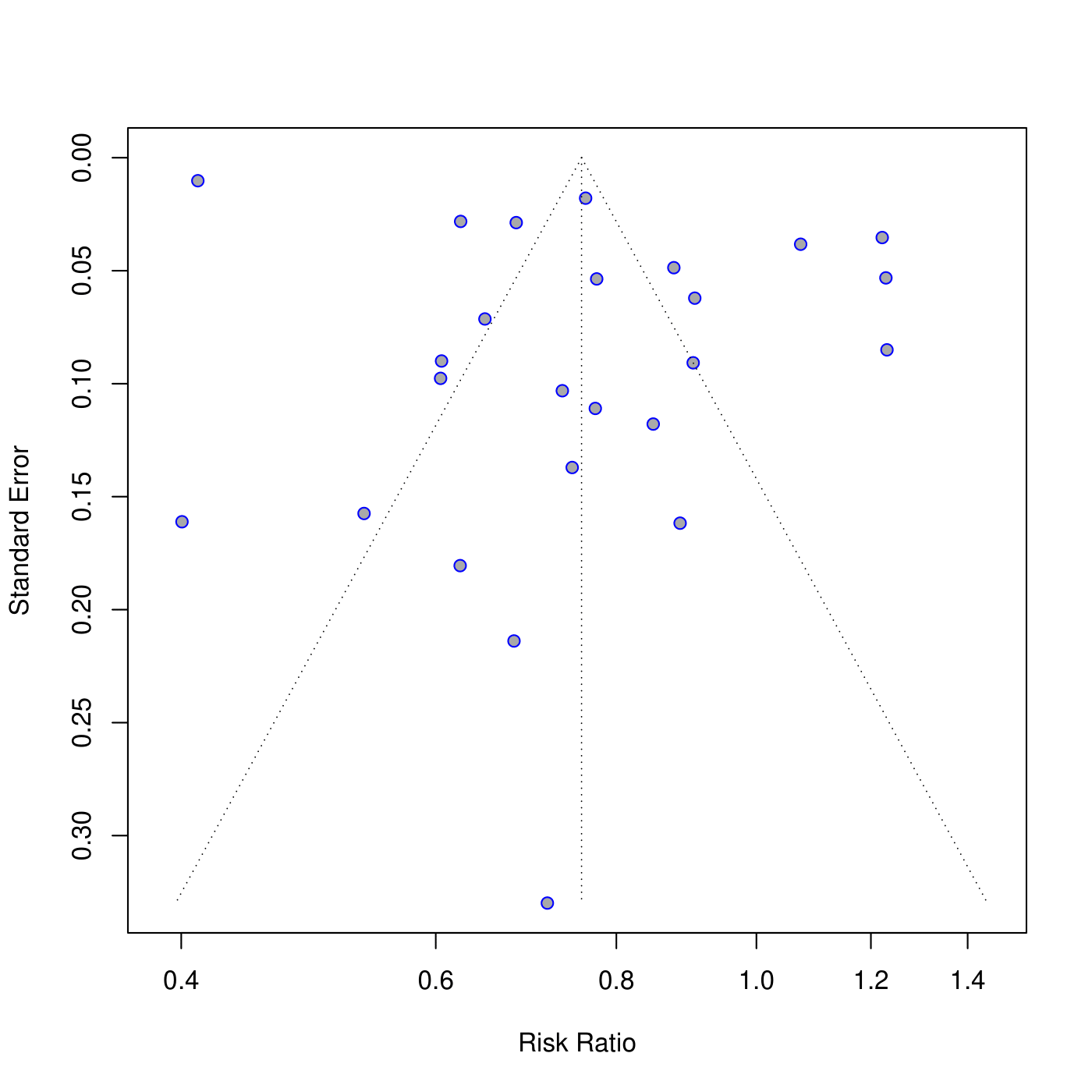


**Figure S27.** Prior HF hospital admission results after excluding studies with high risk of bias
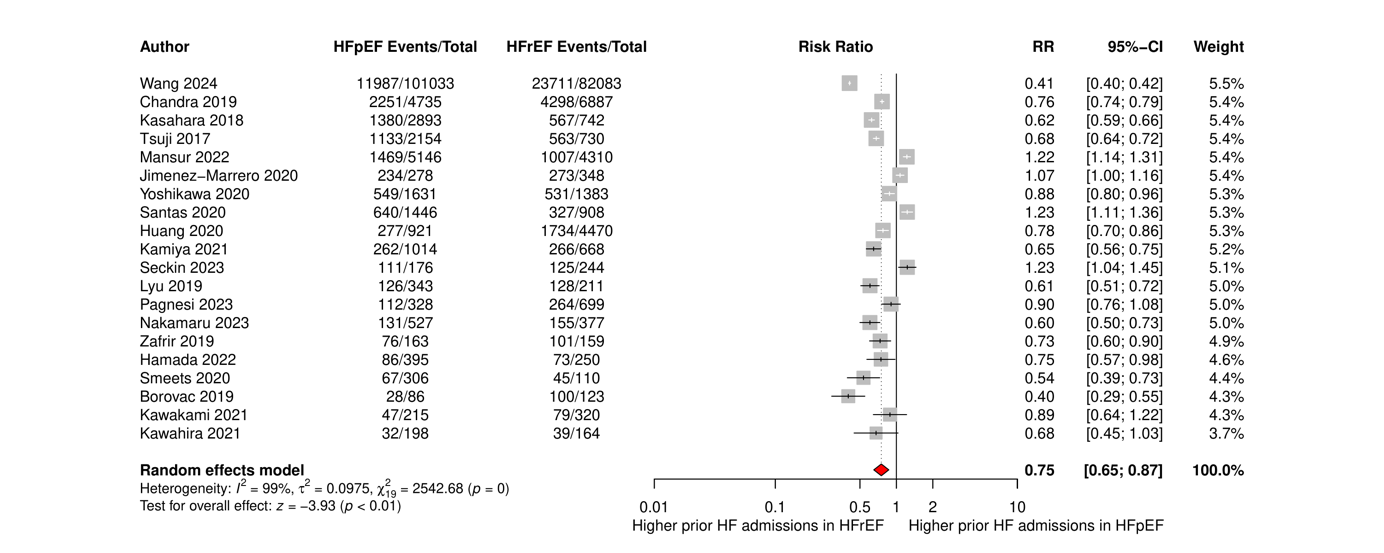


**Figure S28.** Pooled mean difference in length of hospital stay between HFpEF and HFrEF patients
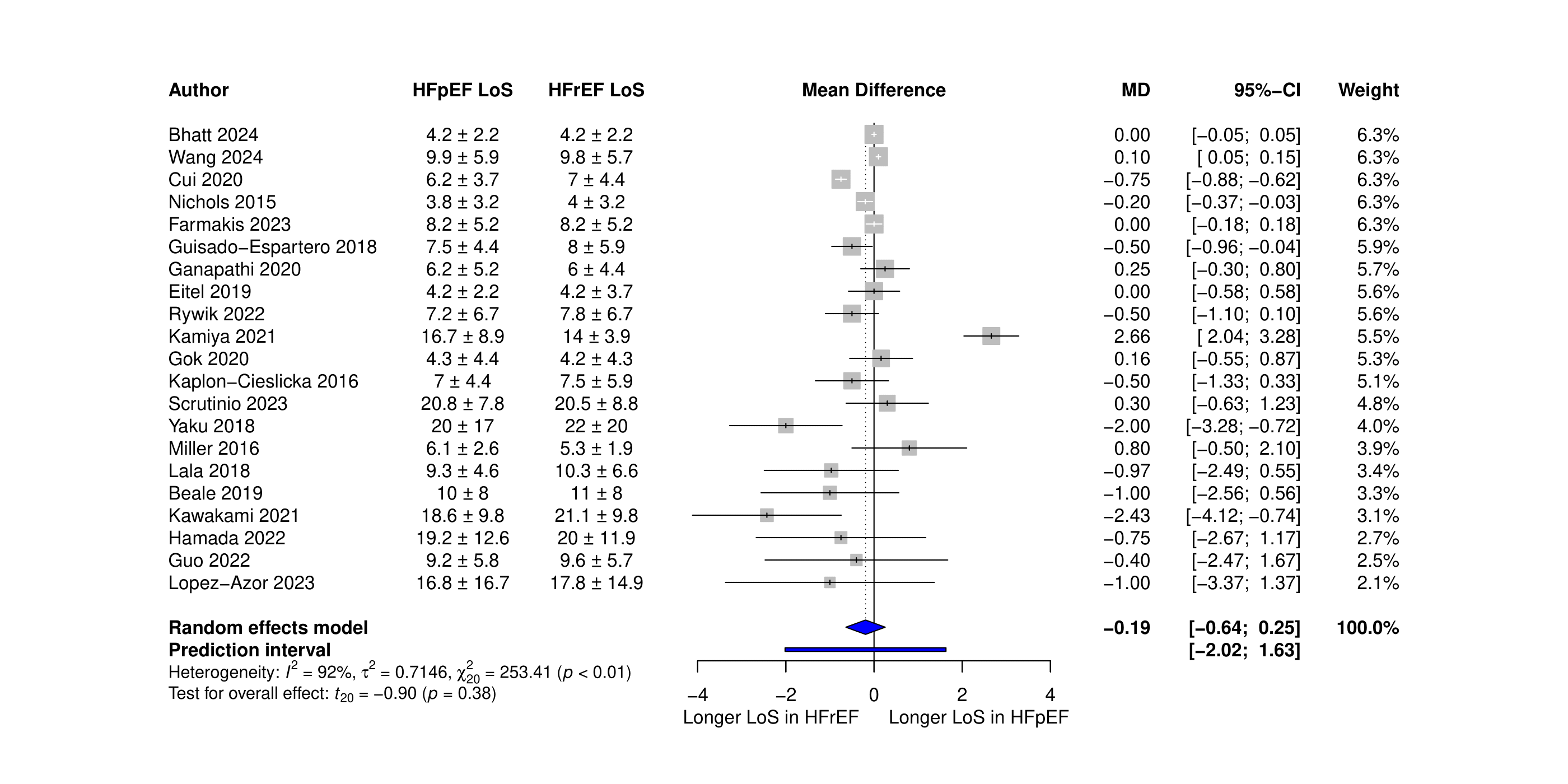


**Figure S29.** Funnel plot for length of hospital stay
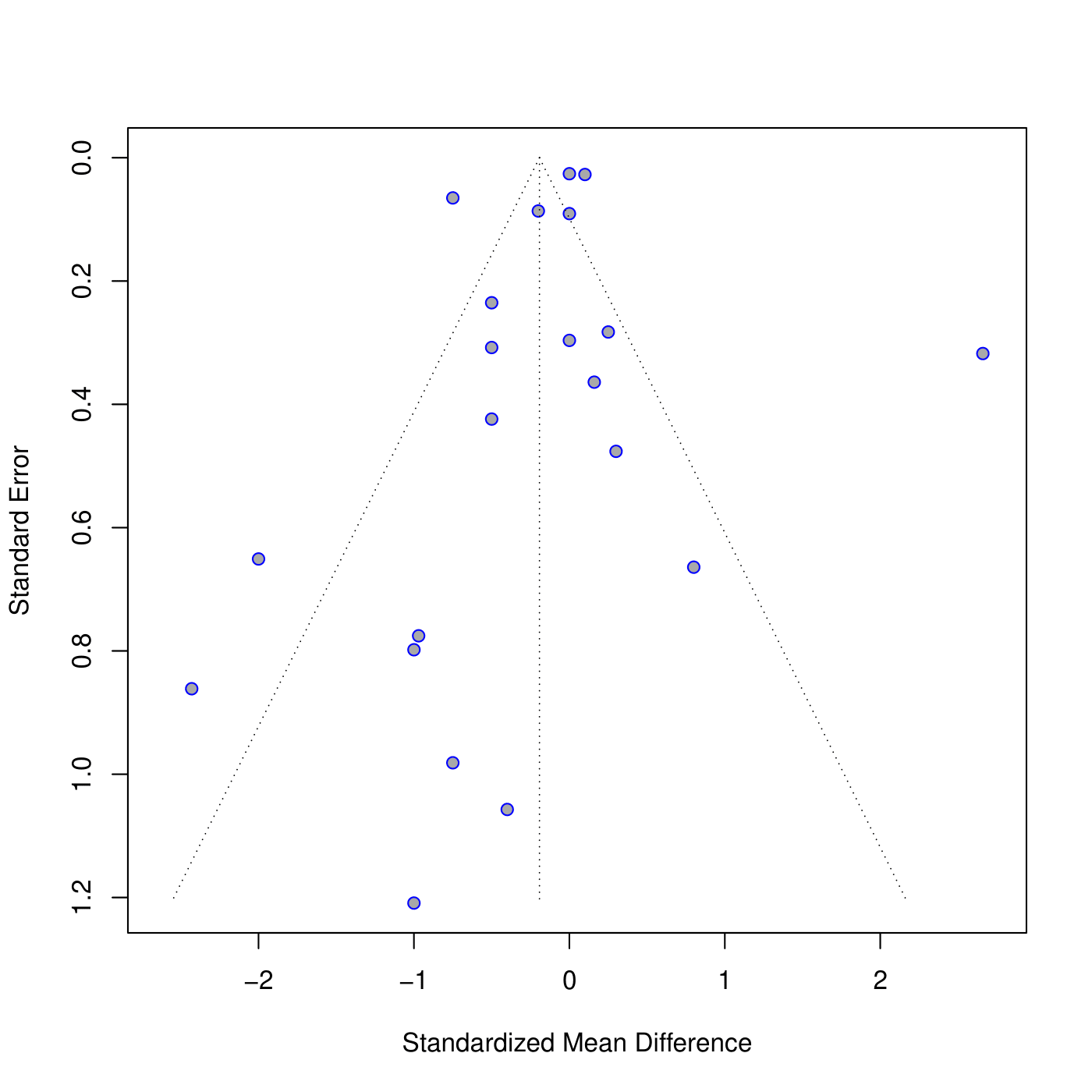


**Figure S30.** Length of hospital stay results after excluding studies with high risk of bias
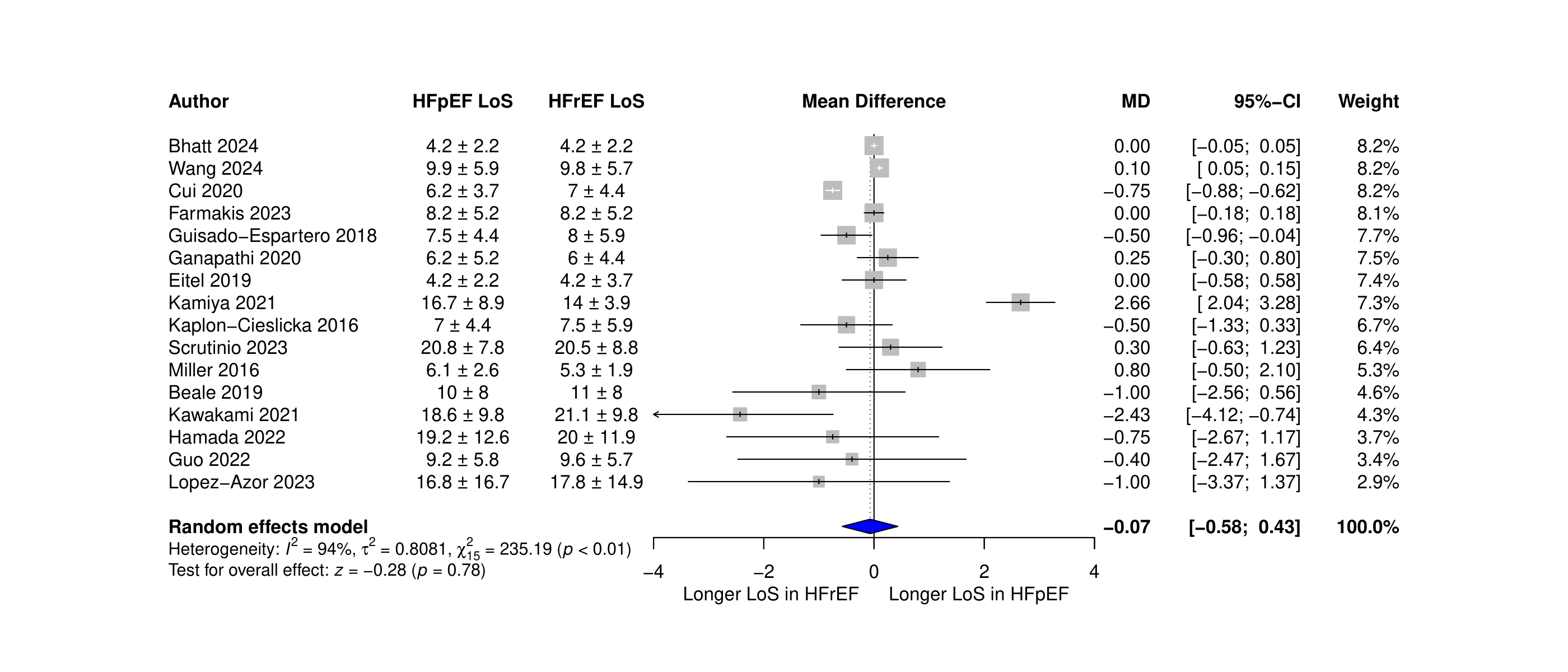


**Figure S31.** Meta-regression analysis of mortality moderated by age
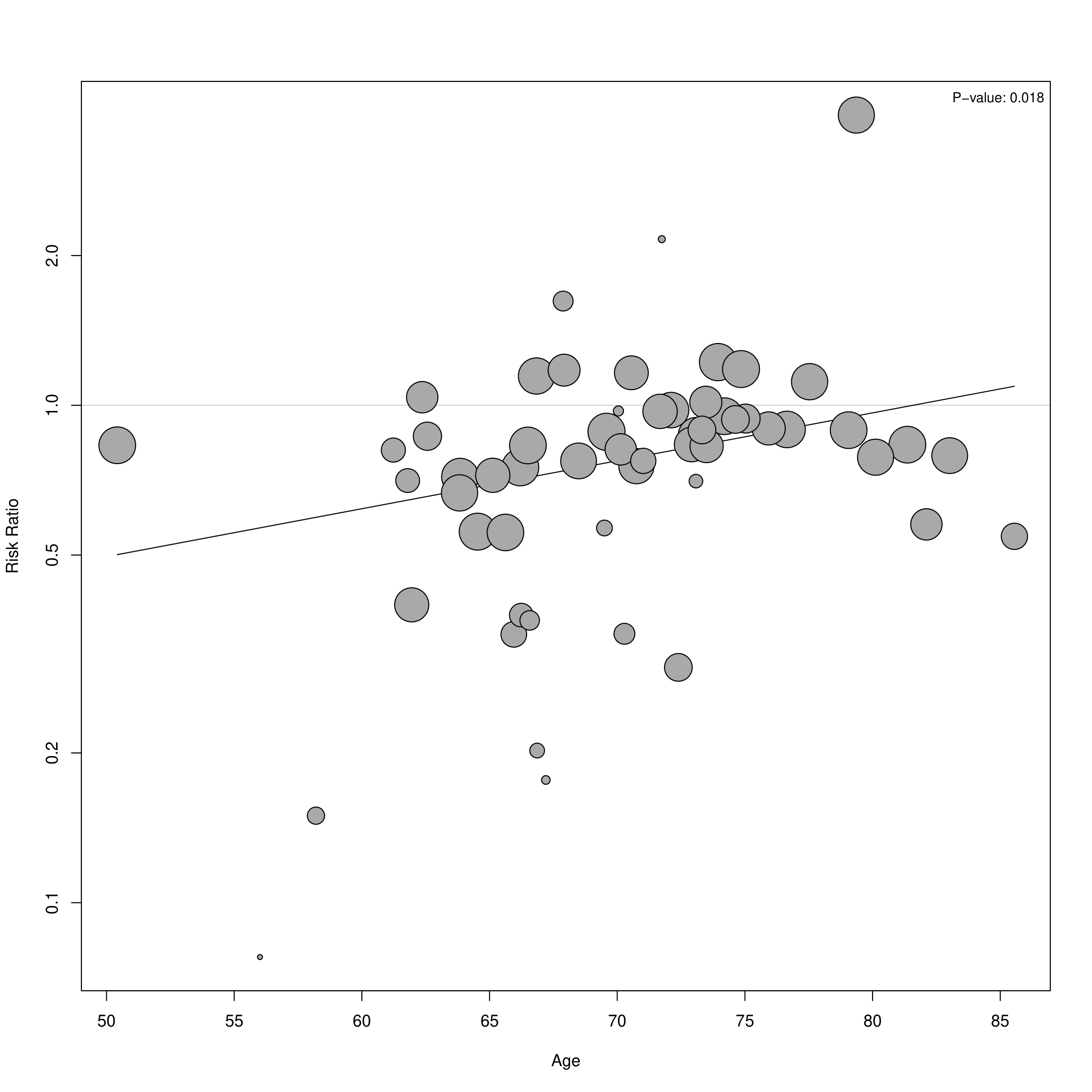


**Figure S32.** Meta-regression analysis of mortality moderated by gender
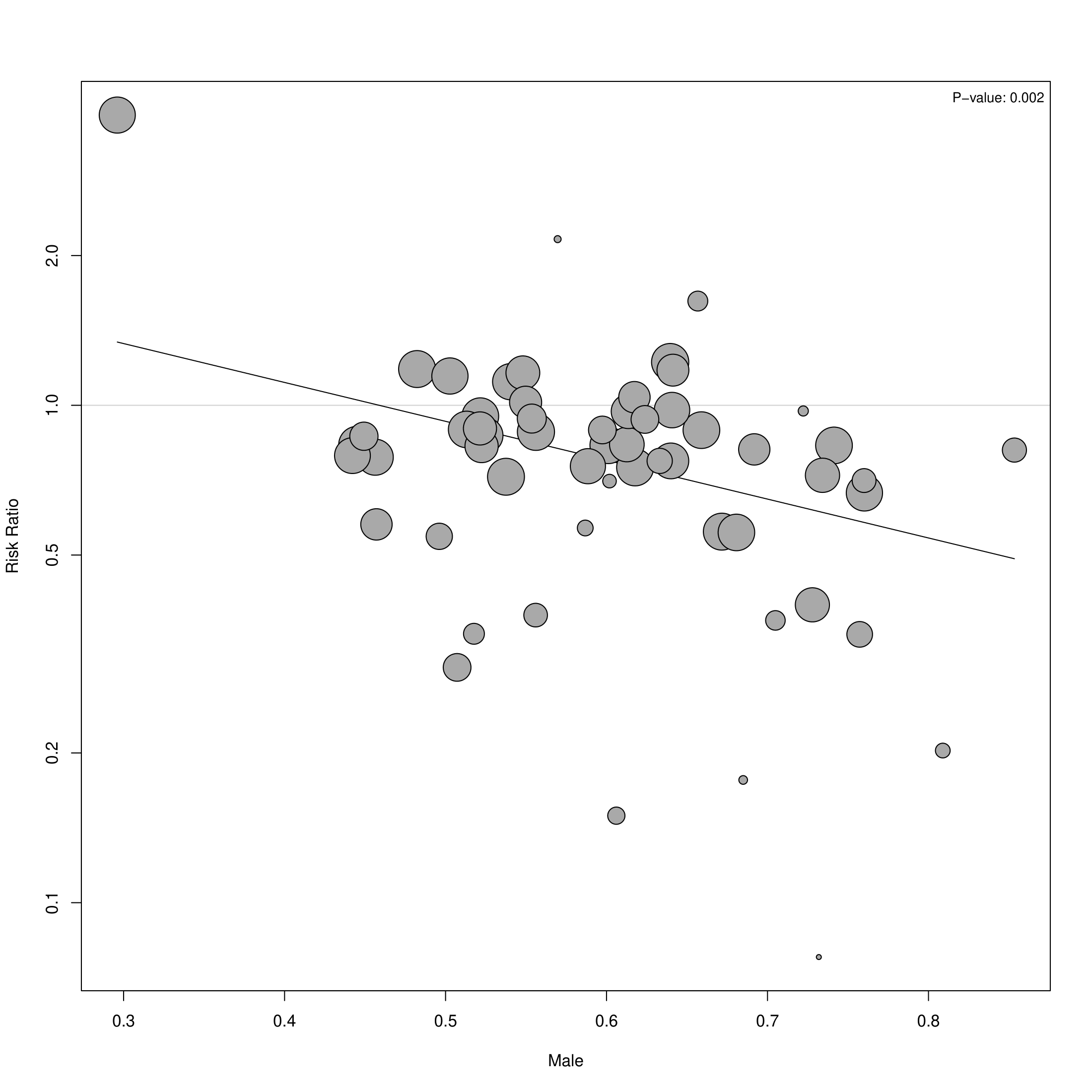


**Figure S33.** Meta-regression analysis of cardiovascular mortality moderated by NT-proBNP levels
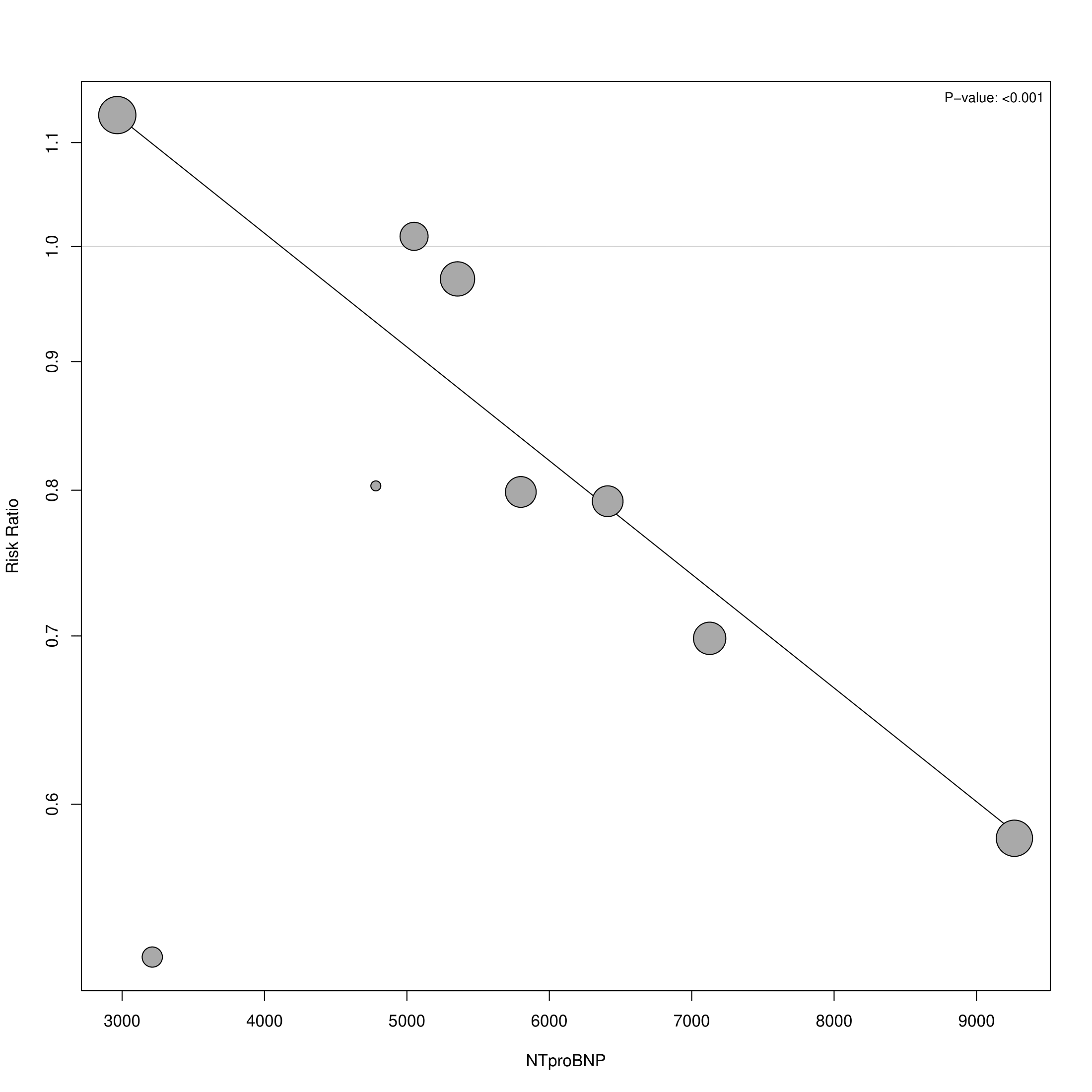


**Figure S34.** Meta-regression analysis of cardiovascular mortality moderated by diabetes
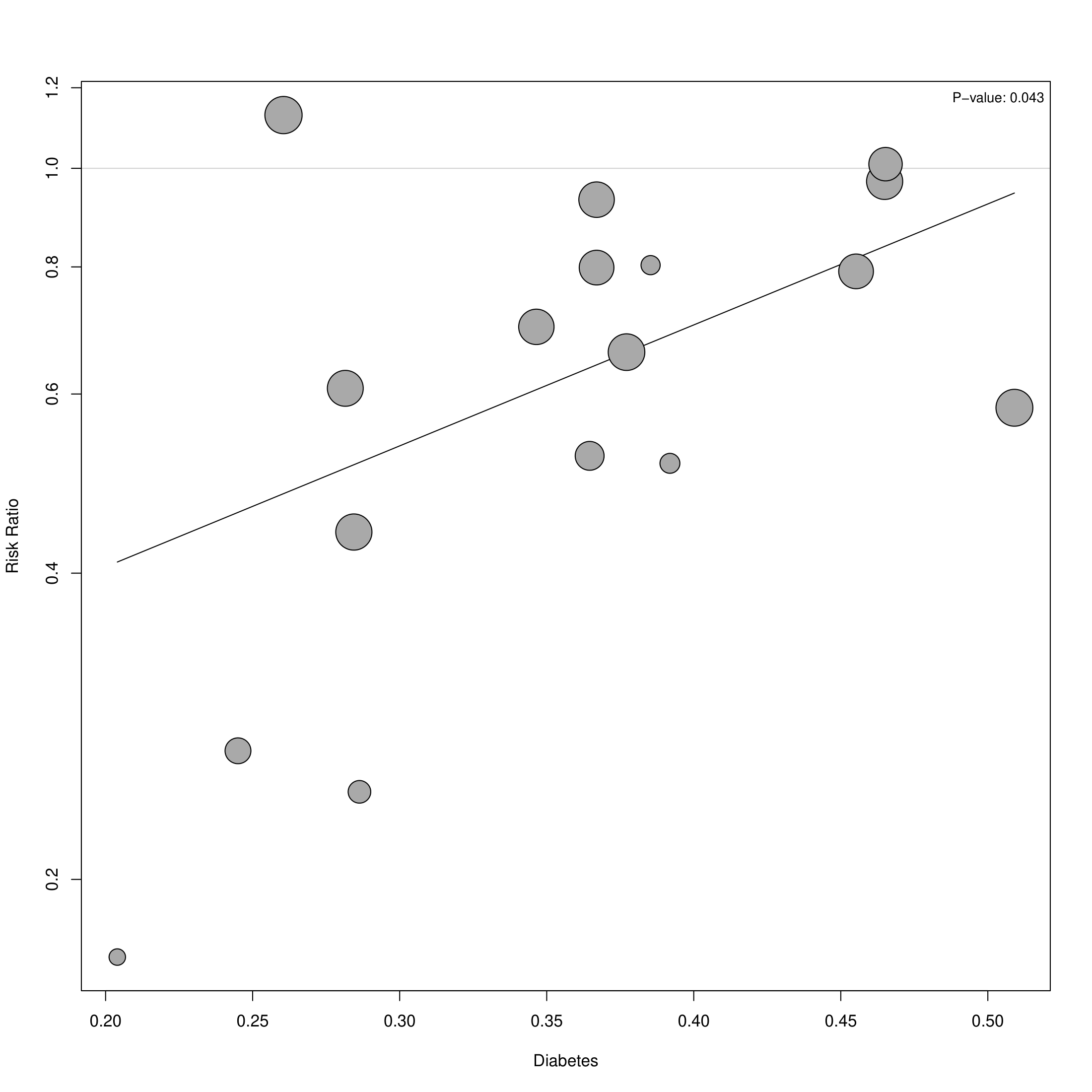


**Figure S35.** Meta-regression analysis of cardiovascular mortality moderated by LVEF differences
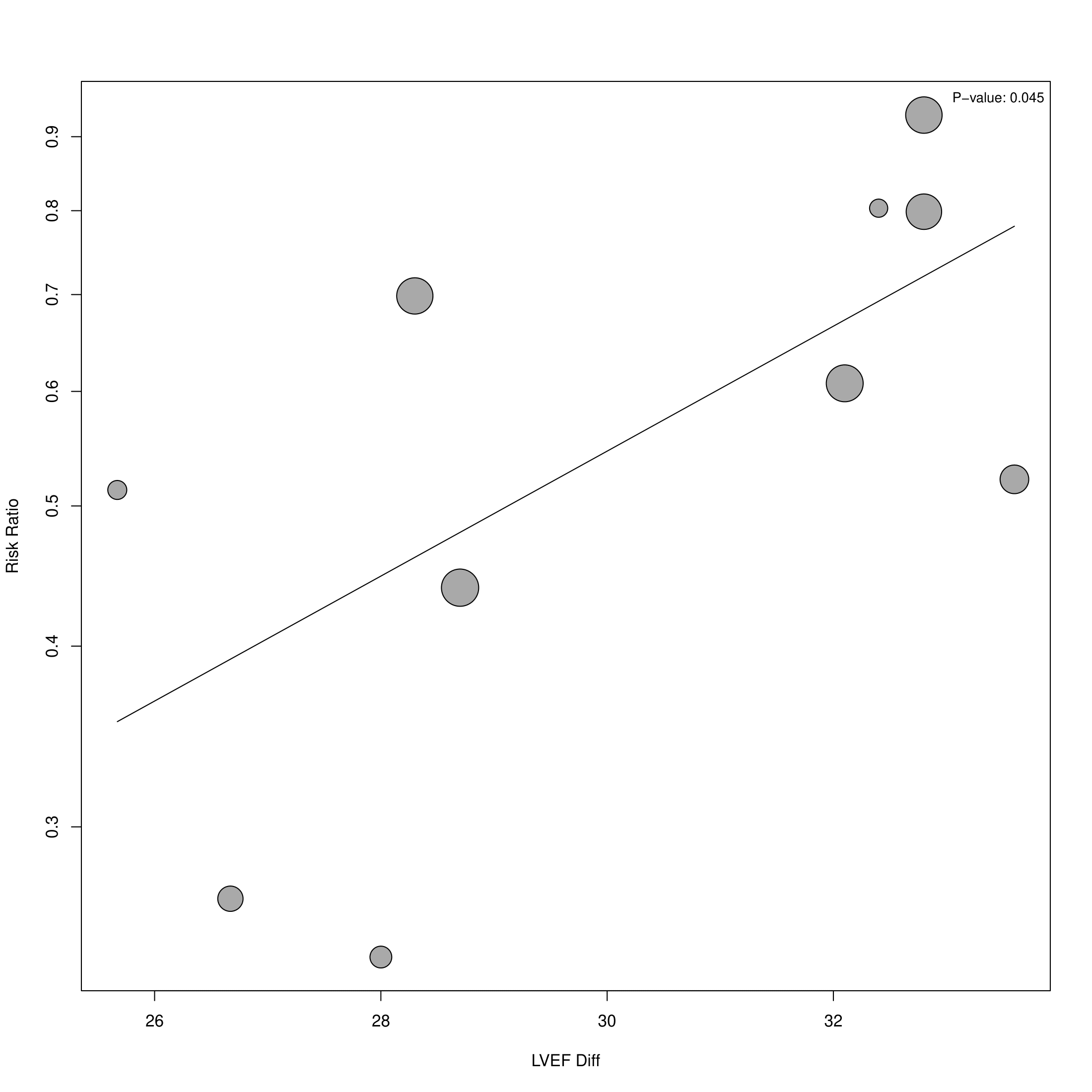


**Figure S36.** Meta-regression analysis of HF hospitalizations moderated by age
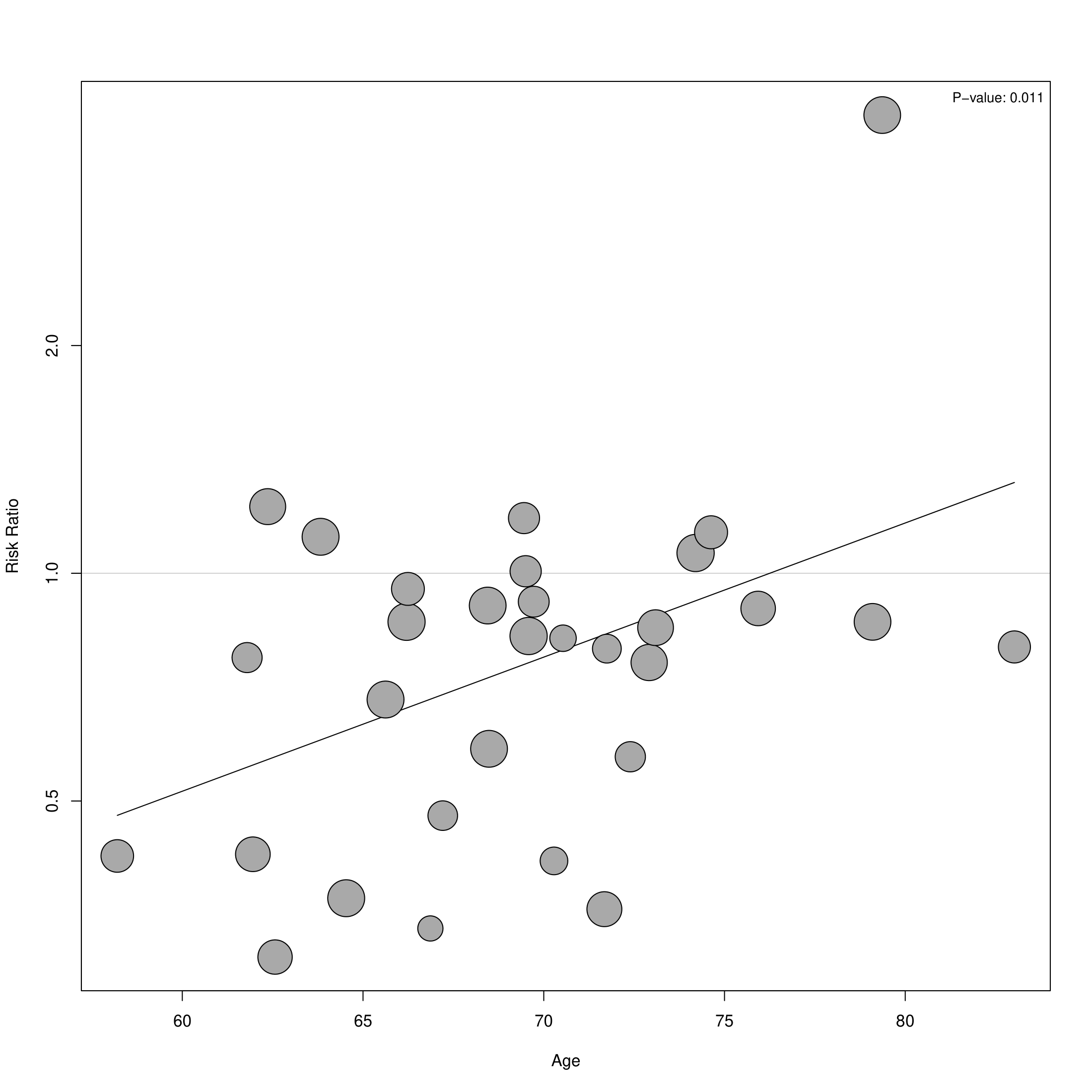


**Figure S37.** Meta-regression analysis of HF hospitalizations moderated by gender
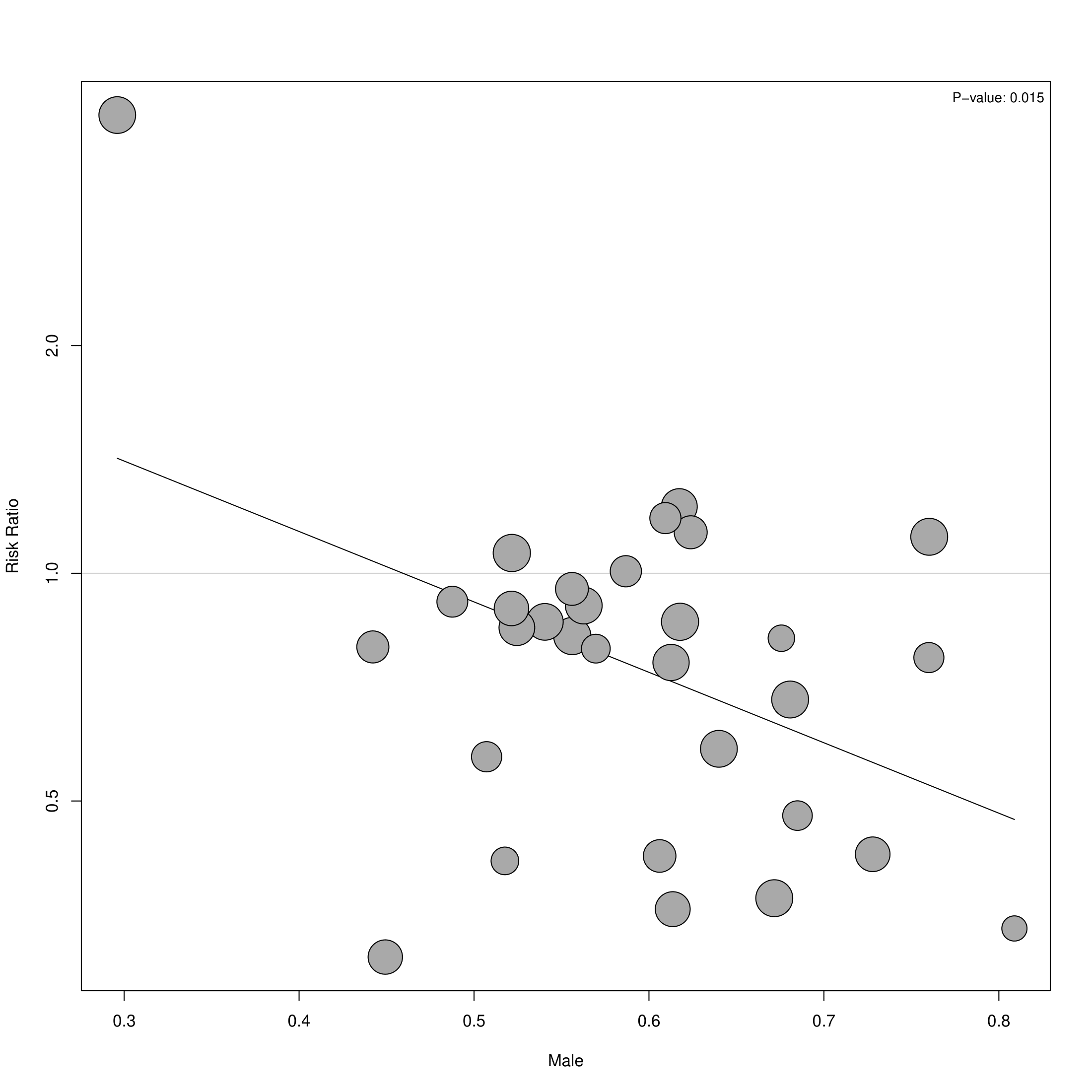


**Figure S38.** Meta-regression analysis of HF hospitalizations moderated by diuretic use
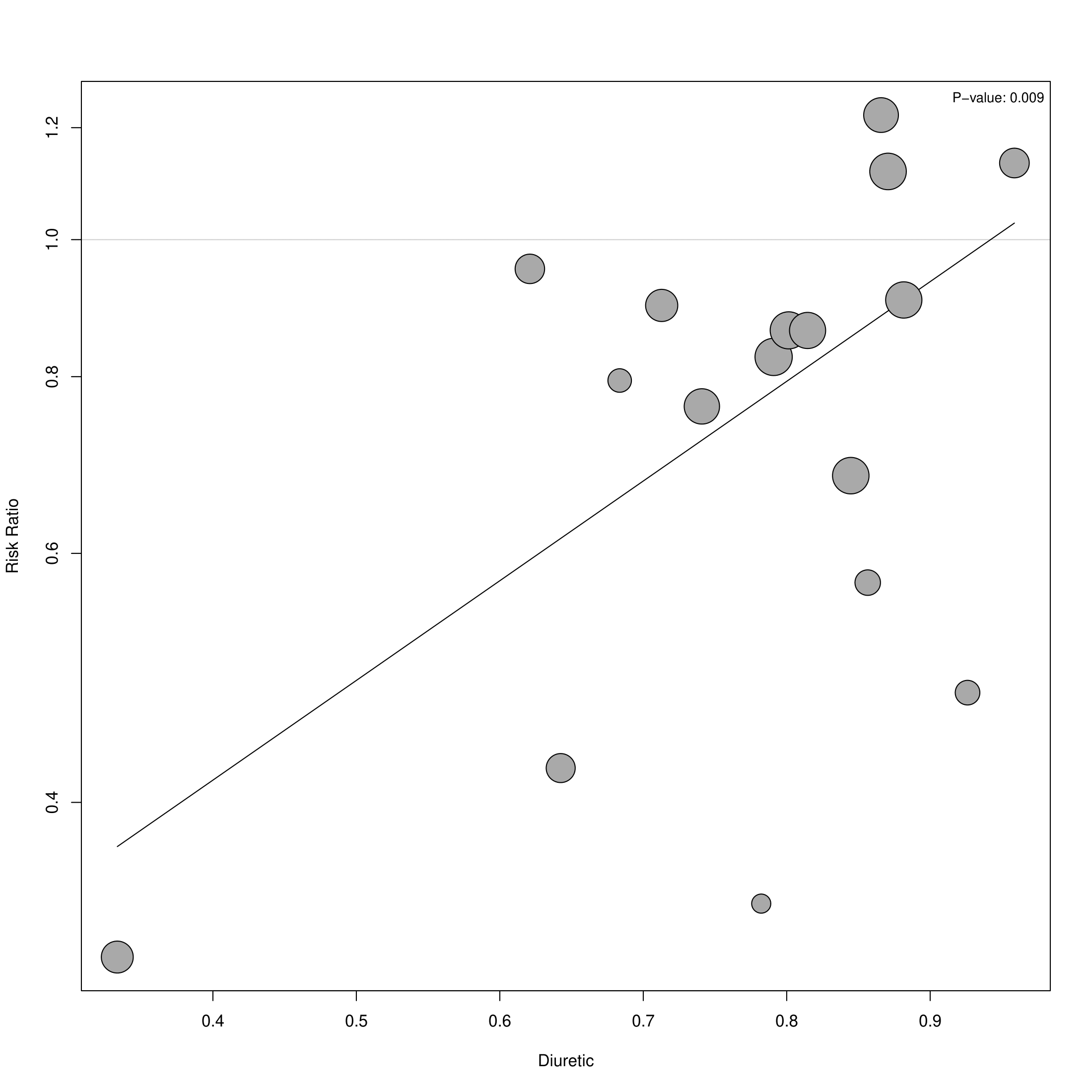


**Figure S39.** Meta-regression analysis of prior HF hospital admissions moderated by BMI
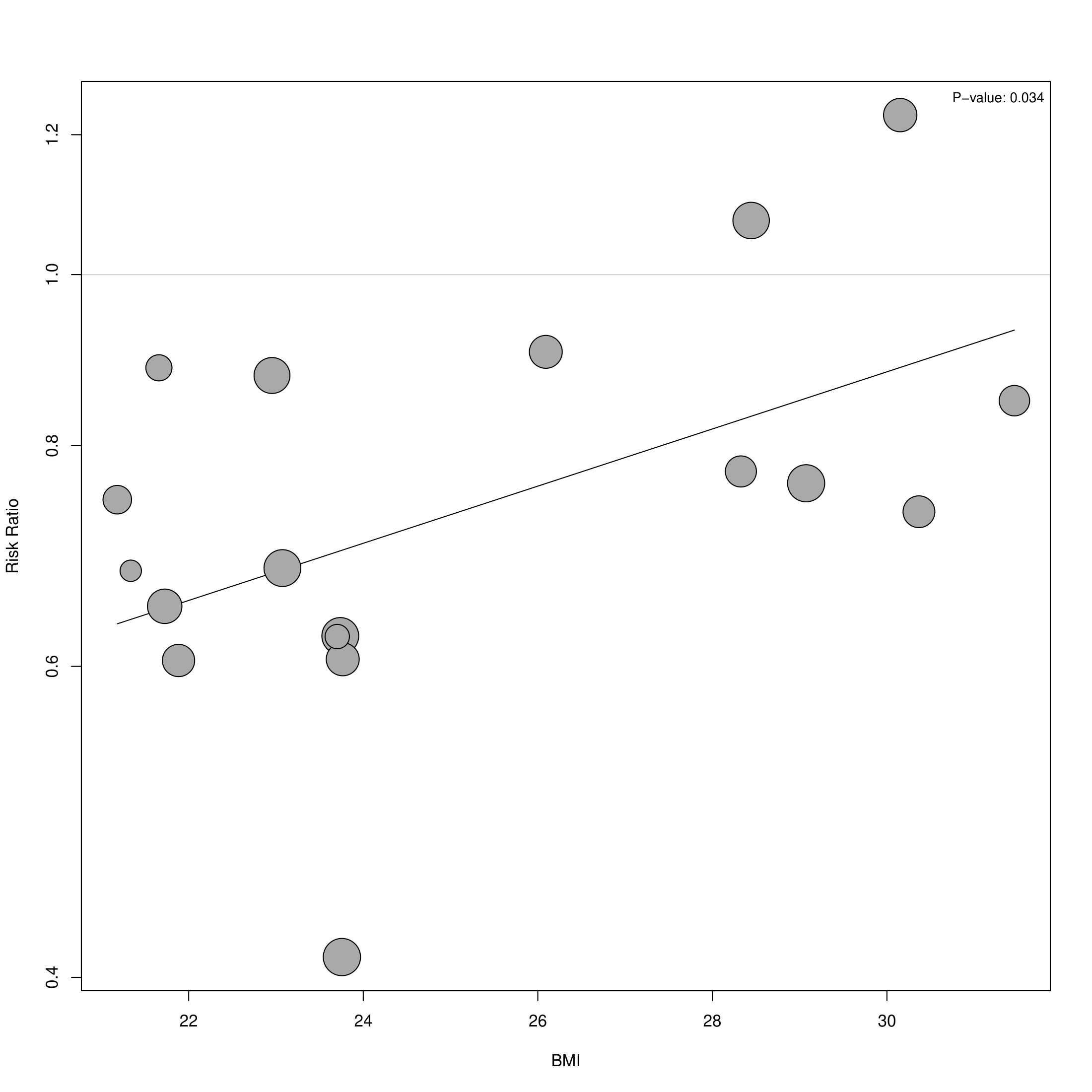

Supplement: xvag026_Supplementary_Data [file xvag026_supplementary_data.zip › Supplementary material.docx]
