# Supplementary material for: Mortality and heart failure hospitalizations in heart failure with preserved ejection fraction compared to heart failure with reduced ejection fraction: a systematic review and meta-analysis
Source: ESC Heart Fail. 2026 Jan 16;13(1):xvag026. doi: 10.1093/eschf/xvag026 (PMC13108283; doi:10.1093/eschf/xvag026)
Supplement: xvag026_Supplementary_Data [file xvag026_supplementary_data.zip › Table S1.docx]

**Table S1.**Search terms employed in the screening based on title, abstract, and keywords in the literature search.

| **Database** | **Search terms** |
| --- | --- |
|  |  |
| PubMed | (“HFpEF” AND “HFrEF”) OR (“Heart failure with preserved ejection fraction” AND “Heart failure with reduced ejection fraction”)  AND ("Mortality" OR "All-cause mortality" OR "Cardiovascular mortality" OR "CV mortality" OR "Hospitalization" OR  "Heart failure hospitalizations" OR “Heart failure admissions” OR "Rehospitalization*" OR “Readmission*” OR "Hospital stay" OR  “Length of stay”) |
| Cochrane Library | (“HFpEF” AND “HFrEF”) OR (“Heart failure with preserved ejection fraction” AND “Heart failure with reduced ejection fraction”)  AND ("Mortality" OR "All-cause mortality" OR "Cardiovascular mortality" OR "CV mortality" OR "Hospitalization" OR  "Heart failure hospitalizations" OR “Heart failure admissions” OR "Rehospitalization*" OR “Readmission*” OR "Hospital stay" OR  “Length of stay”) |
| Web of Science | (“HFpEF” AND “HFrEF”) OR (“Heart failure with preserved ejection fraction” AND “Heart failure with reduced ejection fraction”)  AND ("Mortality" OR "All-cause mortality" OR "Cardiovascular mortality" OR "CV mortality" OR "Hospitalization" OR  "Heart failure hospitalizations" OR “Heart failure admissions” OR "Rehospitalization*" OR “Readmission*” OR "Hospital stay" OR  “Length of stay”) |
| Scopus | (“HFpEF” AND “HFrEF”) OR (“Heart failure with preserved ejection fraction” AND “Heart failure with reduced ejection fraction”)  AND ("Mortality" OR "All-cause mortality" OR "Cardiovascular mortality" OR "CV mortality" OR "Hospitalization" OR  "Heart failure hospitalizations" OR “Heart failure admissions” OR "Rehospitalization*" OR “Readmission*” OR "Hospital stay" OR  “Length of stay”) |
