# Supplementary material for: Mortality and heart failure hospitalizations in heart failure with preserved ejection fraction compared to heart failure with reduced ejection fraction: a systematic review and meta-analysis
Source: ESC Heart Fail. 2026 Jan 16;13(1):xvag026. doi: 10.1093/eschf/xvag026 (PMC13108283; doi:10.1093/eschf/xvag026)
Supplement: xvag026_Supplementary_Data [file xvag026_supplementary_data.zip › Table S2.docx]

Table S2. Data dictionary.

| Variable Name | Description | Possible Values |
| --- | --- | --- |
| **Study Information** |  |  |
| Author | Name of the first author and year of publication of the study. | Open text |
| Study_design | Design of the study. | “Observational”, “Post-hoc analysis of RCT”, “Non-randomized interventional” |
| Recruitment_center | Center or institution where patients were recruited. | Open text |
| Follow_up_time | Duration of the study follow-up period. | Numerical value (years) |
| Start_year | Year of enrollment start. | N |
| End_year | Year of study enrollment ending. | N |
| **Sample Sizes** |  |  |
| Total_number | Total number of patients included in the study. | N |
| HFrEF_number | Number of patients with Heart Failure with reduced Ejection Fraction (HFrEF). | N |
| HFpEF_number | Number of patients with Heart Failure with preserved Ejection Fraction (HFpEF). | N |
| **Baseline Characteristics** |  |  |
| Male_HFrEF | Number of male patients in the HFrEF group. | N |
| Male_HFpEF | Number of male patients in the HFpEF group. | N |
| Age_HFrEF | Age of patients in the HFrEF group. | mean ± SD (years) |
| Age_HFpEF | Age of patients in the HFpEF group. | mean ± SD (years) |
| BMI_HFrEF | Body Mass Index of patients in the HFrEF group. | mean ± SD (kg/m^2^) |
| BMI_HFpEF | Body Mass Index of patients in the HFpEF group. | mean ± SD (kg/m^2^) |
| LVEF_HFrEF | Left Ventricular Ejection Fraction in the HFrEF group. | mean ± SD (% units) |
| LVEF_HFpEF | Left Ventricular Ejection Fraction in the HFpEF group. | mean ± SD (% units) |
| NT_proBNP_HFrEF | N-terminal pro b-type Natriuretic Peptide level in the HFrEF group. | mean ± SD (pg/mL) |
| NT_proBNP_HFpEF | N-terminal pro b-type Natriuretic Peptide level in the HFpEF group. | mean ± SD (pg/mL) |
| **Comorbidities and Clinical Features** |  |  |
| Hypertension_HFrEF | Number of HFrEF patients with hypertension. | N |
| Hypertension_HFpEF | Number of HFpEF patients with hypertension. | N |
| Afib_HFrEF | Number of HFrEF patients with atrial fibrillation. | N |
| Afib_HFpEF | Number of HFpEF patients with atrial fibrillation. | N |
| Diabetes_HFrEF | Number of HFrEF patients with diabetes mellitus. | N |
| Diabetes_HFpEF | Number of HFpEF patients with diabetes mellitus. | N |
| Previous_myocardial_infarction_HFrEF | Number of HFrEF patients with a history of myocardial infarction. | N |
| Previous_myocardial_infarction_HFpEF | Number of HFpEF patients with a history of myocardial infarction. | N |
| Ischemic_HF_etiology_HFrEF | Number of HFrEF patients with ischemic etiology of heart failure. | N |
| Ischemic_HF_etiology_HFpEF | Number of HFpEF patients with ischemic etiology of heart failure. | N |
| NYHA_III_IV_HFrEF | Number of HFrEF patients in New York Heart Association class III or IV. | N |
| NYHA_III_IV_HFpEF | Number of HFpEF patients in New York Heart Association class III or IV. | N |
| Inpatient_HFrEF | Number of HFrEF patients who were hospitalized at baseline. | N |
| Inpatient_HFpEF | Number of HFpEF patients who were hospitalized at baseline. | N |
| **Medications at Baseline** |  |  |
| ACE_ARB_ARNI_HFrEF | Number of HFrEF patients receiving ACE inhibitors, ARBs, or ARNIs. | N |
| ACE_ARB_ARNI_HFpEF | Number of HFpEF patients receiving ACE inhibitors, ARBs, or ARNIs. | N |
| Beta_blockers_HFrEF | Number of HFrEF patients receiving beta blockers. | N |
| Beta_blockers_HFpEF | Number of HFpEF patients receiving beta blockers. | N |
| MRAs_HFrEF | Number of HFrEF patients receiving mineralocorticoid receptor antagonists. | N |
| MRAs_HFpEF | Number of HFpEF patients receiving mineralocorticoid receptor antagonists. | N |
| SGLT2i_HFrEF | Number of HFrEF patients receiving SGLT2 inhibitors (e.g., Dapagliflozin, Empagliflozin). | N |
| SGLT2i_HFpEF | Number of HFpEF patients receiving SGLT2 inhibitors. | N |
| Diuretic_HFrEF | Number of HFrEF patients receiving diuretics. | N |
| Diuretic_HFpEF | Number of HFpEF patients receiving diuretics. | N |
| **Outcomes** |  |  |
| Mortality_HFrEF | Number of deaths in the HFrEF group during the follow-up period. | N |
| Mortality_HFpEF | Number of deaths in the HFpEF group during the follow-up period. | N |
| Adjusted_estimate_Mortality | Adjusted Hazard Ratio for mortality comparison between HFpEF and HFrEF, with 95% CI. | Format: HR value (95% CI) |
| Admissions_HFrEF | Number of HFrEF patients admitted due to heart failure during follow-up. | N |
| Admissions_HFpEF | Number of HFpEF patients admitted due to heart failure during follow-up. | N |
| Adjusted_estimate_Admissions | Adjusted Hazard Ratio for admissions comparison between HFpEF and HFrEF, with 95% CI. | Format: HR value (95% CI) |
| Readmissions_HFrEF | Number of HFrEF patients readmitted due to heart failure during follow-up. | N |
| Readmissions_HFpEF | Number of HFpEF patients readmitted due to heart failure during follow-up. | N |
| Adjusted_estimate_Readmissions | Adjusted Hazard Ratio for readmissions comparison between HFpEF and HFrEF, with 95% CI. | Format: HR value (95% CI) |
| In_hospital_mortality_HFrEF | Number of in-hospital deaths in the HFrEF group. | N |
| In_hospital_mortality_HFpEF | Number of in-hospital deaths in the HFpEF group. | N |
| Adjusted_estimate_In_hospital_mortality | Adjusted Hazard Ratio for in-hospital mortality comparison between HFpEF and HFrEF, with 95% CI. | Format: HR value (95% CI) |
| CVD_mortality_HFrEF | Number of cardiovascular deaths in the HFrEF group during follow-up. | N |
| CVD_mortality_HFpEF | Number of cardiovascular deaths in the HFpEF group during follow-up. | N |
| Adjusted_estimate_CVD_mortality | Adjusted Hazard Ratio for cardiovascular mortality comparison between HFpEF and HFrEF, with 95% CI. | Format: HR value (95% CI) |
| LoS_HFrEF | Length of hospital stay for HFrEF patients. | mean ± SD (days) |
| LoS_HFpEF | Length of hospital stay for HFpEF patients. | mean ± SD (days) |
| Covariates_adjusted_for | List of covariates included in the adjusted model. | Open text |
