# Supplementary material for: Mortality and heart failure hospitalizations in heart failure with preserved ejection fraction compared to heart failure with reduced ejection fraction: a systematic review and meta-analysis
Source: ESC Heart Fail. 2026 Jan 16;13(1):xvag026. doi: 10.1093/eschf/xvag026 (PMC13108283; doi:10.1093/eschf/xvag026)
Supplement: xvag026_Supplementary_Data [file xvag026_supplementary_data.zip › TableS3.docx]

**Table S3.** Studies included in the meta-analysis by outcome.

| **Outcome** | **Number of Studies** | **References** |
| --- | --- | --- |
| All-cause mortality | 62 | [1–62] |
| CV mortality | 20 | [4–7,15,17,18,22,23,26,34,42,43,46,61,63–67] |
| HF hospitalization | 36 | [1,5–8,10,15,18,20–22,25–27,30,33,37–39,45–47,50–52,55,56,61,62,64–66,68–71] |
| In-hospital mortality | 13 | [7,19,26,30,34,60,72–78] |
| Prior HF hospital admissions | 25 | [4,9,27,31,39,46,61,62,65,66,69,79–92] |
| Length of hospital stay | 21 | [4,7,10,11,51,69,73,74,77,81,92–101] |

**References**

1 Bhatia RS, Tu JV, Lee DS, *et al.* Outcome of Heart Failure with Preserved Ejection Fraction in a Population-Based Study. *New England Journal of Medicine*. 2006;355:260–9. doi: 10.1056/NEJMoa051530

2 Lam CSP, Gamble GD, Ling LH, *et al.* Mortality associated with heart failure with preserved vs. reduced ejection fraction in a prospective international multi-ethnic cohort study. *European Heart Journal*. 2018;39:1770–80. doi: 10.1093/eurheartj/ehy005

3 Löfman I, Szummer K, Dahlström U, *et al.* Associations with and prognostic impact of chronic kidney disease in heart failure with preserved, mid‐range, and reduced ejection fraction. *European J of Heart Fail*. 2017;19:1606–14. doi: 10.1002/ejhf.821

4 Wang H, Li Y, Chai K, *et al.* Mortality in patients admitted to hospital with heart failure in China: a nationwide Cardiovascular Association Database-Heart Failure Centre Registry cohort study. *The Lancet Global Health*. 2024;12:e611–22. doi: 10.1016/S2214-109X(23)00605-8

5 Tay JCK, Chia SY, Koh SHM, *et al.* Clinical characteristics and outcomes in Asian patients with heart failure with mildly reduced ejection fraction. *Singapore Medical Journal*. 2024;65:389–96. doi: 10.4103/singaporemedj.SMJ-2021-096

6 Kumar V, Redfield MM, Glasgow A, *et al.* Incident Heart Failure With Mildly Reduced Ejection Fraction: Frequency, Characteristics, and Outcomes. *Journal of Cardiac Failure*. 2023;29:124–34. doi: 10.1016/j.cardfail.2022.10.424

7 Farmakis D, Tromp J, Marinaki S, *et al.* Impact of left ventricular ejection fraction phenotypes on healthcare resource utilization in hospitalized heart failure: a secondary analysis of REPORT‐HF. *European J of Heart Fail*. 2023;25:818–28. doi: 10.1002/ejhf.2833

8 Niedziela JT, Rozentryt P, Nowak J, *et al.* Characteristics and outcomes for patients with heart failure diagnosed according to the universal definition and classification of heart failure. Data from a single-center registry. *Pol Heart J*. 2024;82:391–7. doi: 10.33963/v.phj.99549

9 Mansur ADP, Del Carlo CH, Gonçalinho GHF, *et al.* Sex Differences in Heart Failure Mortality with Preserved, Mildly Reduced and Reduced Ejection Fraction: A Retrospective, Single-Center, Large-Cohort Study. *IJERPH*. 2022;19:16171. doi: 10.3390/ijerph192316171

10 Nichols GA, Reynolds K, Kimes TM, *et al.* Comparison of Risk of Re-hospitalization, All-Cause Mortality, and Medical Care Resource Utilization in Patients With Heart Failure and Preserved Versus Reduced Ejection Fraction. *The American Journal of Cardiology*. 2015;116:1088–92. doi: 10.1016/j.amjcard.2015.07.018

11 Ganapathi S, Jeemon P, Krishnasankar R, *et al.* Early and long‐term outcomes of decompensated heart failure patients in a tertiary‐care centre in India. *ESC Heart Failure*. 2020;7:467–73. doi: 10.1002/ehf2.12600

12 Miró Ò, Conde-Martel A, Llorens P, *et al.* The influence of comorbidities on the prognosis after an acute heart failure decompensation and differences according to ejection fraction: Results from the EAHFE and RICA registries. *European Journal of Internal Medicine*. 2023;111:97–104. doi: 10.1016/j.ejim.2023.02.026

13 Fröhlich H, Rosenfeld N, Täger T, *et al.* Epidemiology and long-term outcome in outpatients with chronic heart failure in Northwestern Europe. *Heart*. 2019;105:1252–9. doi: 10.1136/heartjnl-2018-314256

14 Huang W, Chang H, Lee C, *et al.* Impaired renal function and mortalities in acute heart failure with different phenotypes. *ESC Heart Failure*. 2022;9:2928–36. doi: 10.1002/ehf2.14002

15 Lund LH, Claggett B, Liu J, *et al.* Heart failure with mid‐range ejection fraction in CHARM: characteristics, outcomes and effect of candesartan across the entire ejection fraction spectrum. *European J of Heart Fail*. 2018;20:1230–9. doi: 10.1002/ejhf.1149

16 Migas S, Ellis ML, Wrona B, *et al.* Missed opportunities in heart failure diagnosis and management: study of an urban UK population. *ESC Heart Failure*. 2024;11:2200–13. doi: 10.1002/ehf2.14766

17 Kitai T, Miyakoshi C, Morimoto T, *et al.* Mode of Death Among Japanese Adults With Heart Failure With Preserved, Midrange, and Reduced Ejection Fraction. *JAMA Netw Open*. 2020;3:e204296. doi: 10.1001/jamanetworkopen.2020.4296

18 Abdul‐Rahim AH, Shen L, Rush CJ, *et al.* Effect of digoxin in patients with heart failure and mid‐range (borderline) left ventricular ejection fraction. *European J of Heart Fail*. 2018;20:1139–45. doi: 10.1002/ejhf.1160

19 Chung J, Kim H-L, Kim M-A, *et al.* Sex Differences in Long-Term Clinical Outcomes in Patients Hospitalized for Acute Heart Failure: A Report from the Korean Heart Failure Registry. *Journal of Women’s Health*. 2019;28:1606–13. doi: 10.1089/jwh.2018.7404

20 Ou S-M, Chao C-J, Tsai M-T, *et al.* Echocardiographic features of left ventricular dysfunction and outcomes in chronic kidney disease. *Heart*. 2023;109:134–42. doi: 10.1136/heartjnl-2022-321404

21 Gierula J, Straw S, Cole CA, *et al.* Diabetes mellitus does not alter mortality or hospitalisation risk in patients with newly diagnosed heart failure with preserved ejection fraction: Time to rethink pathophysiological models of disease progression. *Diabetes and Vascular Disease Research*. 2024;21:14791641231224241. doi: 10.1177/14791641231224241

22 Kapłon‐Cieślicka A, Benson L, Chioncel O, *et al.* A comprehensive characterization of acute heart failure with preserved versus mildly reduced versus reduced ejection fraction – insights from the ESC‐HFA EORP Heart Failure Long‐Term Registry. *European J of Heart Fail*. 2022;24:335–50. doi: 10.1002/ejhf.2408

23 Van Essen BJ, Tromp J, Ter Maaten JM, *et al.* Characteristics and clinical outcomes of patients with acute heart failure with a supranormal left ventricular ejection fraction. *European J of Heart Fail*. 2023;25:35–42. doi: 10.1002/ejhf.2695

24 Jarkovsky J, Spinar J, Tyl B, *et al.* Heart rate as an independent predictor of long term mortality of acute heart failure patients in sinus rhythm according to their ejection fraction: data from the AHEAD registry. *European Journal of Internal Medicine*. 2020;78:88–94. doi: 10.1016/j.ejim.2020.04.022

25 Hamatani Y, Nagai T, Shiraishi Y, *et al.* Long-Term Prognostic Significance of Plasma B-Type Natriuretic Peptide Level in Patients With Acute Heart Failure With Reduced, Mid-Range, and Preserved Ejection Fractions. *The American Journal of Cardiology*. 2018;121:731–8. doi: 10.1016/j.amjcard.2017.12.012

26 Gómez-Otero I, Ferrero-Gregori A, Varela Román A, *et al.* Mid-range Ejection Fraction Does Not Permit Risk Stratification Among Patients Hospitalized for Heart Failure. *Revista Española de Cardiología (English Edition)*. 2017;70:338–46. doi: 10.1016/j.rec.2016.11.016

27 Huang W, Teng TK, Tay WT, *et al.* Patient‐reported outcomes in heart failure with preserved vs. reduced ejection fraction: focus on physical independence. *ESC Heart Failure*. 2020;7:2051–62. doi: 10.1002/ehf2.12950

28 Mirzai S, Persits I, Martens P, *et al.* Significance of Adipose Tissue Quantity and Distribution on Obesity Paradox in Heart Failure. *The American Journal of Cardiology*. 2023;207:339–48. doi: 10.1016/j.amjcard.2023.08.136

29 Cristóbal H, Enjuanes C, Batlle M, *et al.* Prognostic Value of Soluble AXL in Serum from Heart Failure Patients with Preserved and Reduced Left Ventricular Ejection Fraction. *JPM*. 2023;13:446. doi: 10.3390/jpm13030446

30 Shiga T, Suzuki A, Haruta S, *et al.* Clinical characteristics of hospitalized heart failure patients with preserved, mid‐range, and reduced ejection fractions in Japan. *ESC Heart Failure*. 2019;6:475–86. doi: 10.1002/ehf2.12418

31 Kawahira M, Tamaki S, Yamada T, *et al.* Prognostic value of impaired hepato‐renal function and liver fibrosis in patients admitted for acute heart failure. *ESC Heart Failure*. 2021;8:1274–83. doi: 10.1002/ehf2.13195

32 Hamazaki N, Kamiya K, Matsuzawa R, *et al.* Prevalence and prognosis of respiratory muscle weakness in heart failure patients with preserved ejection fraction. *Respiratory Medicine*. 2020;161:105834. doi: 10.1016/j.rmed.2019.105834

33 Al-Jarallah M, Rajan R, Al-Zakwani I, *et al.* Mortality and Morbidity in HFrEF, HFmrEF, and HFpEF Patients with Diabetes in the Middle East. *Oman Med J*. 2020;35:e99–e99. doi: 10.5001/omj.2020.17

34 Fischer-Rasokat U, Renker M, Liebetrau C, *et al.* Outcome of patients with heart failure after transcatheter aortic valve implantation. *PLoS ONE*. 2019;14:e0225473. doi: 10.1371/journal.pone.0225473

35 Kerwagen F, Koehler K, Vettorazzi E, *et al.* Remote patient management of heart failure across the ejection fraction spectrum: A pre‐specified analysis of the TIM‐HF2 trial. *European J of Heart Fail*. 2023;25:1671–81. doi: 10.1002/ejhf.2948

36 Iwatsu K, Ikeda T, Matsumura K, *et al.* Gap in the prognostic impact of short physical performance battery among phenotypes of heart failure. *International Journal of Cardiology*. 2022;361:85–90. doi: 10.1016/j.ijcard.2022.05.005

37 Chairat K, Rattanavipanon W, Tanyasaensook K, *et al.* Relationship of anemia and clinical outcome in heart failure patients with preserved versus reduced ejection fraction in a rural area of Thailand. *IJC Heart & Vasculature*. 2020;30:100597. doi: 10.1016/j.ijcha.2020.100597

38 Wierda E, Van Maarschalkerwaart W (W. A), Van Seumeren E, *et al.* Outpatient treatment of worsening heart failure with intravenous diuretics: first results from a multicentre 2‐year experience. *ESC Heart Failure*. 2023;10:594–600. doi: 10.1002/ehf2.14168

39 Borovac JA, Novak K, Bozic J, *et al.* The midrange left ventricular ejection fraction (LVEF) is associated with higher all-cause mortality during the 1-year follow-up compared to preserved LVEF among real-world patients with acute heart failure: a single-center propensity score-matched analysis. *Heart Vessels*. 2019;34:268–78. doi: 10.1007/s00380-018-1249-7

40 Anastasio F, Testa M, Ferreri C, *et al.* The Analysis of Arterial Stiffness in Heart Failure Patients: The Prognostic Role of Pulse Wave Velocity, Augmentation Index and Stiffness Index. *JCM*. 2022;11:3507. doi: 10.3390/jcm11123507

41 Imamura Y, Suzuki A, Kamishima K, *et al.* Prognostic factors in patients with heart failure and sarcopenia: an observational retrospective study. *Egypt Heart J*. 2024;76:52. doi: 10.1186/s43044-024-00484-4

42 Thuijs DJFM, Milojevic M, Stone GW, *et al.* Impact of left ventricular ejection fraction on clinical outcomes after left main coronary artery revascularization: results from the randomized EXCEL trial. *European J of Heart Fail*. 2020;22:871–9. doi: 10.1002/ejhf.1681

43 Cenkerova K, Dubrava J, Pokorna V, *et al.* Prognostic value of echocardiography and ECG in heart failure with preserved ejection fraction. *BLL*. 2016;117:407–12. doi: 10.4149/BLL_2016_080

44 Lin T, Hsu B, Li Y, *et al.* Prognostic Value of Anxiety Between Heart Failure With Reduced Ejection Fraction and Heart Failure With Preserved Ejection Fraction. *JAHA*. 2019;8:e010739. doi: 10.1161/JAHA.118.010739

45 Song Y, Li F, Xu Y, *et al.* Prognostic value of sST2 in patients with heart failure with reduced, mid-range and preserved ejection fraction. *International Journal of Cardiology*. 2020;304:95–100. doi: 10.1016/j.ijcard.2020.01.039

46 Lyu S, Yu L, Tan H, *et al.* Clinical characteristics and prognosis of heart failure with mid-range ejection fraction: insights from a multi-centre registry study in China. *BMC Cardiovasc Disord*. 2019;19:209. doi: 10.1186/s12872-019-1177-1

47 Pan W, Yang D, Yu P, *et al.* Comparison of predictive value of NT-proBNP, sST2 and MMPs in heart failure patients with different ejection fractions. *BMC Cardiovasc Disord*. 2020;20:208. doi: 10.1186/s12872-020-01493-2

48 Tromp J, Kosiborod MN, Angermann CE, *et al.* Treatment effects of empagliflozin in hospitalized heart failure patients across the range of left ventricular ejection fraction – Results from the EMPULSE trial. *European J of Heart Fail*. 2024;26:963–70. doi: 10.1002/ejhf.3218

49 Fujimoto H, Doi N, Okayama S, *et al.* Long-term prognosis of patients undergoing radiofrequency catheter ablation for atrial fibrillation: comparison between heart failure subtypes based on left ventricular ejection fraction. *EP Europace*. 2022;24:576–86. doi: 10.1093/europace/euab201

50 Gong J, Castro RRT, Caron JP, *et al.* Usefulness of ventilatory inefficiency in predicting prognosis across the heart failure spectrum. *ESC Heart Failure*. 2022;9:293–302. doi: 10.1002/ehf2.13761

51 Guo S, Gong M, Tse G, *et al.* The Value of IGF-1 and IGFBP-1 in Patients With Heart Failure With Reduced, Mid-range, and Preserved Ejection Fraction. *Front Cardiovasc Med*. 2022;8:772105. doi: 10.3389/fcvm.2021.772105

52 Zeller J, Hubauer U, Schober A, *et al.* Heart failure with recovered ejection fraction (HFrecEF): A new entity with improved cardiac outcome. *Pacing Clinical Electrophis*. 2021;44:2015–23. doi: 10.1111/pace.14391

53 Wu J, Chen M, Wang H, *et al.* Comparison of Characteristics and Outcomes Between Acute Ischemic Stroke Patients with Different Types of Heart Failure. *Int Heart J*. 2024;65:94–9. doi: 10.1536/ihj.22-717

54 Bouwmeester S, Van Der Stam JA, Van Loon SLM, *et al.* Left atrial reservoir strain as a predictor of cardiac outcome in patients with heart failure: the HaFaC cohort study. *BMC Cardiovasc Disord*. 2022;22:104. doi: 10.1186/s12872-022-02545-5

55 Xu X-H, Sun B, Zhong S, *et al.* Diabetic retinopathy predicts cardiovascular mortality in diabetes: a meta-analysis. *BMC Cardiovasc Disord*. 2020;20:478. doi: 10.1186/s12872-020-01763-z

56 Van Aelst LNL, Arrigo M, Placido R, *et al.* Acutely decompensated heart failure with preserved and reduced ejection fraction present with comparable haemodynamic congestion. *European J of Heart Fail*. 2018;20:738–47. doi: 10.1002/ejhf.1050

57 Wernhart S, Papathanasiou M, Rassaf T, *et al.* Heart failure classification based on resting ejection fraction does not display a unique exercise response pattern. *International Journal of Cardiology*. 2023;376:157–64. doi: 10.1016/j.ijcard.2023.01.072

58 Dunlay SM, Roger VL, Killian JM, *et al.* Advanced Heart Failure Epidemiology and Outcomes: A Population-Based Study. *JACC Heart Fail*. 2021;9:722–32. doi: 10.1016/j.jchf.2021.05.009

59 Xu S-D, Su G-H, Lu Y-X, *et al.* Elevated Soluble ST2 and Depression Increased the Risk of All-Cause Mortality and Hospitalization in Patients With Heart Failure. *Int Heart J*. 2014;55:445–50. doi: 10.1536/ihj.13-371

60 Wang K, Li H, Bei W, *et al.* Association of left ventricular ejection fraction with contrast-induced nephropathy and mortality following coronary angiography or intervention in patients with heart failure. *TCRM*. 2017;Volume 13:887–95. doi: 10.2147/TCRM.S137654

61 Pagnesi M, Lombardi CM, Tedino C, *et al.* Role of ejection fraction in patients at risk for advanced heart failure: insights from the HELP‐HF registry. *ESC Heart Failure*. 2024;11:136–46. doi: 10.1002/ehf2.14539

62 Santas E, De La Espriella R, Palau P, *et al.* Rehospitalization burden and morbidity risk in patients with heart failure with mid‐range ejection fraction. *ESC Heart Failure*. 2020;7:1007–14. doi: 10.1002/ehf2.12683

63 Popovic D, Arena R, Guazzi M. A flattening oxygen consumption trajectory phenotypes disease severity and poor prognosis in patients with heart failure with reduced, mid‐range, and preserved ejection fraction. *European J of Heart Fail*. 2018;20:1115–24. doi: 10.1002/ejhf.1140

64 Gargani L, Pugliese NR, Frassi F, *et al.* Prognostic value of lung ultrasound in patients hospitalized for heart disease irrespective of symptoms and ejection fraction. *ESC Heart Failure*. 2021;8:2660–9. doi: 10.1002/ehf2.13206

65 Yoshikawa Y, Tamaki Y, Morimoto T, *et al.* Impact of left ventricular ejection fraction on the effect of renin-angiotensin system blockers after an episode of acute heart failure: From the KCHF Registry. *PLoS ONE*. 2020;15:e0239100. doi: 10.1371/journal.pone.0239100

66 Zhirov I, Safronova N, Osmolovskaya Y, *et al.* Predictors of Unfavorable Outcomes in Patients with Atrial Fibrillation and Concomitant Heart Failure with Different Ejection Fractions: RIF-CHF Register One-Year Follow-Up. *Cardiology Research and Practice*. 2019;2019:1–14. doi: 10.1155/2019/1692104

67 Settergren C, Benson L, Shahim A, *et al.* Cause‐specific death in heart failure across the ejection fraction spectrum: A comprehensive assessment of over 100 000 patients in the Swedish Heart Failure Registry. *European J of Heart Fail*. 2024;26:1150–9. doi: 10.1002/ejhf.3230

68 Hage C, Löfgren L, Michopoulos F, *et al.* Metabolomic Profile in HFpEF vs HFrEF Patients. *Journal of Cardiac Failure*. 2020;26:1050–9. doi: 10.1016/j.cardfail.2020.07.010

69 Kamiya K, Adachi T, Iwatsu K, *et al.* Association between a pre-admission limitation in walking ability and post-discharge adverse outcomes among hospitalized patients with heart failure: Report from a multicenter prospective cohort study. *International Journal of Cardiology*. 2021;337:105–12. doi: 10.1016/j.ijcard.2021.05.020

70 Kusunose K, Hirata Y, Yamaguchi N, *et al.* Deep learning approach for analyzing chest x-rays to predict cardiac events in heart failure. *Front Cardiovasc Med*. 2023;10:1081628. doi: 10.3389/fcvm.2023.1081628

71 Tomasoni D, Vitale C, Guidetti F, *et al.* The role of multimorbidity in patients with heart failure across the left ventricular ejection fraction spectrum: Data from the Swedish Heart Failure Registry. *European J of Heart Fail*. 2024;26:854–68. doi: 10.1002/ejhf.3112

72 Bonapace S, Rossi A, Laroche C, *et al.* Brachial pulse pressure in acute heart failure. Results of the Heart Failure Registry. *ESC Heart Failure*. 2019;6:1167–77. doi: 10.1002/ehf2.12537

73 Gök G, Kılıç S, Sinan ÜY, *et al.* Epidemiology and clinical characteristics of hospitalized elderly patients for heart failure with reduced, mid-range and preserved ejection fraction. *Heart & Lung*. 2020;49:495–500. doi: 10.1016/j.hrtlng.2020.03.023

74 Rywik TM, Doryńska A, Wiśniewska A, *et al.* Epidemiology and clinical characteristics of hospitalized heart failure patients with a reduced, mildly reduced and preserved ejection fraction. *Polish Archives of Internal Medicine*. Published Online First: 7 March 2022. doi: 10.20452/pamw.16227

75 Subki AH, Almalki MA, Butt NS, *et al.* Echocardiographic and Clinical Correlates of Ejection Fraction Among 2000 Patients with Heart Failure in Western Saudi Arabia. *IJGM*. 2020;Volume 13:281–8. doi: 10.2147/IJGM.S251924

76 Takei M, Kohsaka S, Shiraishi Y, *et al.* Heart Failure With Midrange Ejection Fraction in Patients Admitted for Acute Decompensation: A Report from the Japanese Multicenter Registry. *Journal of Cardiac Failure*. 2019;25:666–73. doi: 10.1016/j.cardfail.2019.05.010

77 Yaku H, Ozasa N, Morimoto T, *et al.* Demographics, Management, and In-Hospital Outcome of Hospitalized Acute Heart Failure Syndrome Patients in Contemporary Real Clinical Practice in Japan ― Observations From the Prospective, Multicenter Kyoto Congestive Heart Failure (KCHF) Registry ―. *Circ J*. 2018;82:2811–9. doi: 10.1253/circj.CJ-17-1386

78 Kim HM, Kim H-L, Kim M-A, *et al.* Sex differences in clinical characteristics and long-term outcome in patients with heart failure: data from the KorAHF registry. *Korean J Intern Med*. 2024;39:95–109. doi: 10.3904/kjim.2023.288

79 Chandra A, Vaduganathan M, Lewis EF, *et al.* Health-Related Quality of Life in Heart Failure With Preserved Ejection Fraction. *JACC: Heart Failure*. 2019;7:862–74. doi: 10.1016/j.jchf.2019.05.015

80 Fudim M, Kelly JP, Jones AD, *et al.* Are existing and emerging biomarkers associated with cardiorespiratory fitness in patients with chronic heart failure? *American Heart Journal*. 2020;220:97–107. doi: 10.1016/j.ahj.2019.11.006

81 Hamada T, Kubo T, Kawai K, *et al.* Clinical characteristics and frailty status in heart failure with preserved vs. reduced ejection fraction. *ESC Heart Failure*. 2022;9:1853–63. doi: 10.1002/ehf2.13885

82 Ito M, Wada H, Sakakura K, *et al.* Clinical Characteristics and Long-Term Outcomes of Patients with Acute Decompensated Heart Failure with Mid-Range Ejection Fraction. *Int Heart J*. 2019;60:862–9. doi: 10.1536/ihj.18-631

83 Jimenez-Marrero S, Moliner P, Rodríguez-Costoya I, *et al.* Sympathetic activation and outcomes in chronic heart failure: Does the neurohormonal hypothesis apply to mid-range and preserved ejection fraction patients? *European Journal of Internal Medicine*. 2020;81:60–6. doi: 10.1016/j.ejim.2020.07.008

84 Nakamaru R, Shiraishi Y, Kohno T, *et al.* Treatment patterns and trajectories in patients after acute heart failure hospitalization. *ESC Heart Failure*. 2024;11:692–701. doi: 10.1002/ehf2.14635

85 Seckin M, Johnston B, Petrie MC, *et al.* Characteristics of symptoms and symptom change across different heart failure subtypes: a sex-stratified analysis. *European Journal of Cardiovascular Nursing*. 2023;22:690–700. doi: 10.1093/eurjcn/zvac099

86 Shukkoor AA, George NE, Radhakrishnan S, *et al.* Clinical characteristics and outcomes of patients admitted with acute heart failure: insights from a single-center heart failure registry in South India. *Egypt Heart J*. 2021;73:38. doi: 10.1186/s43044-021-00161-w

87 Tromp J, Khan MAF, Mentz RJ, *et al.* Biomarker Profiles of Acute Heart Failure Patients With a Mid-Range Ejection Fraction. *JACC Heart Fail*. 2017;5:507–17. doi: 10.1016/j.jchf.2017.04.007

88 Tsuji K, Sakata Y, Nochioka K, *et al.* Characterization of heart failure patients with mid‐range left ventricular ejection fraction—a report from the CHART ‐2 Study. *European J of Heart Fail*. 2017;19:1258–69. doi: 10.1002/ejhf.807

89 Zafrir B, Carasso S, Goland S, *et al.* The impact of left ventricular ejection fraction on heart failure patients with pulmonary hypertension. *Heart & Lung*. 2019;48:502–6. doi: 10.1016/j.hrtlng.2019.05.006

90 Smeets M, Vaes B, Aertgeerts B, *et al.* Impact of an extended audit on identifying heart failure patients in general practice: baseline results of the OSCAR‐HF pilot study. *ESC Heart Failure*. 2020;7:3950–61. doi: 10.1002/ehf2.12990

91 Kasahara S, Sakata Y, Nochioka K, *et al.* Comparable prognostic impact of BNP levels among HFpEF, Borderline HFpEF and HFrEF: a report from the CHART-2 Study. *Heart Vessels*. 2018;33:997–1007. doi: 10.1007/s00380-018-1150-4

92 Kawakami R, Nakada Y, Hashimoto Y, *et al.* Prevalence and Prognostic Significance of Pulmonary Function Test Abnormalities in Hospitalized Patients With Acute Decompensated Heart Failure With Preserved and Reduced Ejection Fraction. *Circ J*. 2021;85:1426–34. doi: 10.1253/circj.CJ-20-1069

93 Beale A, Carballo D, Stirnemann J, *et al.* Iron Deficiency in Acute Decompensated Heart Failure. *JCM*. 2019;8:1569. doi: 10.3390/jcm8101569

94 Bhatt AS, Fonarow GC, Greene SJ, *et al.* Medical Therapy Before, During and After Hospitalization in Medicare Beneficiaries With Heart Failure and Diabetes: Get With The Guidelines – Heart Failure Registry. *Journal of Cardiac Failure*. 2024;30:319–28. doi: 10.1016/j.cardfail.2023.09.005

95 Cui X, Thunström E, Dahlström U, *et al.* Trends in cause‐specific readmissions in heart failure with preserved vs. reduced and mid‐range ejection fraction. *ESC Heart Failure*. 2020;7:2894–903. doi: 10.1002/ehf2.12899

96 Eitel C, Ince H, Brachmann J, *et al.* Atrial fibrillation ablation strategies and outcome in patients with heart failure: insights from the German ablation registry. *Clin Res Cardiol*. 2019;108:815–23. doi: 10.1007/s00392-019-01411-3

97 Kapłon-Cieślicka A, Tymińska A, Peller M, *et al.* Diagnosis, Clinical Course, and 1-Year Outcome in Patients Hospitalized for Heart Failure With Preserved Ejection Fraction (from the Polish Cohort of the European Society of Cardiology Heart Failure Long-Term Registry). *Am J Cardiol*. 2016;118:535–42. doi: 10.1016/j.amjcard.2016.05.046

98 Lala RI, Lungeanu D, Puschita M, *et al.* Acute kidney injury: a clinical issue in hospitalized patients with heart failure with mid­‑range ejection fraction. *POLISH ARCHIVES OF INTERNAL MEDICINE*.

99 López-Azor JC, Delgado JF, Vélez J, *et al.* Differences in clinical outcomes, health care resource utilization and costs in heart failure patients according to left ventricular ejection fraction. *Revista Española de Cardiología (English Edition)*. 2023;76:862–71. doi: 10.1016/j.rec.2023.06.003

100 Miller WL, Mullan BP. Volume Overload Profiles in Patients With Preserved and Reduced Ejection Fraction Chronic Heart Failure. *JACC: Heart Failure*. 2016;4:453–9. doi: 10.1016/j.jchf.2016.01.005

101 Scrutinio D, Guida P, La Rovere MT, *et al.* Functional outcome after cardiac rehabilitation and its association with survival in heart failure across the spectrum of ejection fraction. *European Journal of Internal Medicine*. 2023;110:86–92. doi: 10.1016/j.ejim.2023.02.002
