# Supplementary material for: Mortality and heart failure hospitalizations in heart failure with preserved ejection fraction compared to heart failure with reduced ejection fraction: a systematic review and meta-analysis
Source: ESC Heart Fail. 2026 Jan 16;13(1):xvag026. doi: 10.1093/eschf/xvag026 (PMC13108283; doi:10.1093/eschf/xvag026)
Supplement: xvag026_Supplementary_Data [file xvag026_supplementary_data.zip › TableS4.docx]

**Table S4.** Overview of studies with adjusted models for investigated outcomes.

| Author | Covariates adjusted for | Outcome |
| --- | --- | --- |
| Al-Jarallah 2020 | Age, sex, BMI, smoking, khat, PVD, HTN, DM, prior stroke/TIA, SBP, DBP, Cr, PCI/CABG, admission diagnosis, NYHA, in-hospital course, discharged medications. | All-cause mortality, HF hospitalization |
| Bhatia 2006 | Age, sex, coexisting conditions. | All-cause mortality |
| Borovac 2019 | Age, sex, NYHA, LVEDd, eGFR, urea, UA, Na, K, Hgb, SBP, DBP, prior hospitalizations, medications. | All-cause mortality, HF hospitalization |
| Dunlay 2021 | Age, sex, Charlson comorbidity index. | All-cause mortality |
| Farmakis 2023 | Age, sex, HTN, AF, COPD, CKD, CAD, ACEi/ARB, BB, MRA, diuretics, race, HF diagnosis, in-hospital services, transport mode. | All-cause mortality, CVD mortality |
| Gomez-Otero 2017 | Age, sex, HF history, DM, HTN, COPD, OSA, stroke, PVD, HR, SBP, ischemic etiology, Hgb, eGFR, NT-proBNP > 1500 ng/L, Charlson index, Barthel index, Pfeiffer test, medications. | All-cause mortality |
| Imamura 2024 | Age, NYHA, BNP. | All-cause mortality |
| Kamiya 2021 | Age. | HF hospitalization |
| Kaplon-Cieslicka 2022 | Age, female, BMI, ischemic HF, prior HF hospitalization, stroke/TIA, AF, PVD, DM, COPD, hepatic dysfunction, cancer, depression, SBP, NYHA III-IV, labs, inotropes, medications. | All-cause mortality, HF hospitalization |
| Kitai 2020 | Age, sex, HTN, DM, AF, anemia, CKD, albumin, BUN, BB, ACEi/ARB, MRA. | All-cause mortality, CVD mortality |
| Kumar 2023 | Age, sex, Charlson index. | All-cause mortality, HF hospitalization, CVD mortality |
| Lam 2018 | Age, sex, HTN, DM, ischemic etiology, NYHA, AF, SBP, HR, LBBB, Cr. | All-cause mortality |
| Lund 2018 | Sex, ethnicity, NYHA, SBP, HF cause, prior HF hospitalization, AF, stroke, DM, smoking, cancer, stratified by candesartan, age, BMI. | All-cause mortality, HF hospitalization, CVD mortality |
| Lyu 2019 | Sex, non-solitary, MoCA, smoking, ACEi/ARB. | All-cause mortality, HF hospitalization, CVD mortality |
| Migas 2024 | Comorbidities (unspecified). | All-cause mortality |
| Miro 2023 | Age, sex, baseline status, comorbidities. | All-cause mortality |
| Nichols 2015 | Age, sex, race, smoking, prior HF, CAD, DM, HTN, CKD, depression. | All-cause mortality, HF hospitalization |
| Ou 2023 | Age, sex, HbA1c, eGFR, UACR, UPCR, HTN, DM, CAD, malignancy, medications. | All-cause mortality, HF hospitalization |
| Pagnesi 2023 | Age, sex, inpatient/outpatient, advanced HF definition, PAD, stroke/TIA, AF. | All-cause mortality, HF hospitalization, CVD mortality |
| Santas 2020 | Prior MI, COPD, NYHA III-IV, SBP, HR, eGFR. | All-cause mortality, HF hospitalization |
| Settergren 2024 | Age, sex, year. | CVD mortality |
| Shiga 2019 | Age, CVD, HTN, DM, dyslipidemia, hyperuricemia, COPD, CKD, anemia, SBP. | All-cause mortality |
| Tay 2023 | Age, sex, race, prior MI, AF, DM, HTN, stroke, PVD, COPD, SBP, DBP, HR, QRS duration, Cr, Na, K, Hgb, medications. | All-cause mortality, HF hospitalization, CVD mortality |
| Tomasoni 2024 | Sex, age, smoking, care location, education, income, prior HF hospitalization, NYHA, MAP, HR, EF, K, NT-proBNP, medications, HF devices. | HF hospitalization |
| van Essen 2022 | Cr, Hgb, Na, BUN, CVA, COPD, PVD, RR, SBP, BMI, edema, IV loop diuretics, DM, prior HF hospitalization, NT-proBNP, sex, age. | All-cause mortality, CVD mortality |
| Wang 2017 | Not reported. | All-cause mortality |
| Wang 2024 | Variables based on subject matter knowledge, biological plausibility, and multicollinearity tests. | All-cause mortality, CVD mortality |
| Xu 2014 | Age, HR, BMI, Cr, NYHA, HTN, depression, sST2, NT-proBNP. | All-cause mortality |
| Zeller 2021 | Age, HTN, DM, CKD, DCM, ICD indication. | All-cause mortality |

**Abbreviations:** ACEi/ARB, Angiotensin-Converting Enzyme Inhibitor / Angiotensin Receptor Blocker; AF, Atrial Fibrillation; BB, Beta-Blocker; BMI, Body Mass Index; BNP, B-type Natriuretic Peptide; BUN, Blood Urea Nitrogen; CAD, Coronary Artery Disease; CKD, Chronic Kidney Disease; COPD, Chronic Obstructive Pulmonary Disease; Cr, Creatinine; CVA, Cerebrovascular Accident; CVD, Cardiovascular Disease; DCM, Dilated Cardiomyopathy; DBP, Diastolic Blood Pressure; DM, Diabetes Mellitus; eGFR, Estimated Glomerular Filtration Rate; EF, Ejection Fraction; HbA1c, Hemoglobin A1c; Hgb, Hemoglobin; HF, Heart Failure; HR, Heart Rate; HTN, Hypertension; ICD, Implantable Cardioverter-Defibrillator; IV, Intravenous; K, Potassium; LBBB, Left Bundle Branch Block; LVEDd, Left Ventricular End-Diastolic Dimention; MAP, Mean Arterial Pressure; MI, Myocardial Infarction; MoCA, Montreal Cognitive Assessment; MRA, Mineralocorticoid Receptor Antagonist; Na, Sodium; NT-proBNP, N-terminal pro-B-type Natriuretic Peptide; NYHA, New York Heart Association; OSA, Obstructive Sleep Apnea; PAD, Peripheral Artery Disease; PVD, Peripheral Vascular Disease; RR, Respiration Rate; SBP, Systolic Blood Pressure; sST2, Soluble Suppression of Tumorigenicity 2; TIA, Transient Ischemic Attack; UA, Uric Acid; UACR, Urine Albumin-to-Creatinine Ratio; UPCR, Urine Protein-to-Creatinine Ratio.
