# Supplementary material for: Mortality and heart failure hospitalizations in heart failure with preserved ejection fraction compared to heart failure with reduced ejection fraction: a systematic review and meta-analysis
Source: ESC Heart Fail. 2026 Jan 16;13(1):xvag026. doi: 10.1093/eschf/xvag026 (PMC13108283; doi:10.1093/eschf/xvag026)
Supplement: xvag026_Supplementary_Data [file xvag026_supplementary_data.zip › TableS5.docx]

Table S5. Meta-regression results.

| **All-cause mortality** | | | | | | | |
| --- | --- | --- | --- | --- | --- | --- | --- |
| Moderator | Coefficient (95% CI) | Standard error | Z value | P value | Q | R^2^ | Tau^2^ |
| Age | 0.022 (0.004; 0.040) | 0.009 | 2.438 | 0.018 | 1072 | 9.4 | 0.146 |
| BMI | -0.003 (-0.053; 0.047) | 0.025 | -0.131 | 0.897 | 373 | 0.0 | 0.090 |
| NT-proBNP | 2.281(-5.543; 10.105) x 10^-5^ | 3.751 x 10^-5^ | 0.608 | 0.550 | 685 | 0.0 | 0.172 |
| Male sex | -1.800 (-2.924; -0.675) | 0.561 | -3.208 | 0.002 | 1399 | 24.4 | 0.122 |
| Hypertension | 0.021 (-0.810; 0.851) | 0.413 | 0.050 | 0.960 | 1359 | 0.0 | 0.183 |
| AF | 0.150 (-0.696; 0.995) | 0.416 | 0.359 | 0.722 | 532 | 0.0 | 0.064 |
| Diabetes | 0.886 (-0.533; 2.305) | 0.707 | 1.254 | 0.216 | 1292 | 0.0 | 0.170 |
| Previous MI | -0.105 (-1.601; 1.392) | 0.702 | -0.149 | 0.883 | 735 | 0.0 | 0.122 |
| Ischemic Etiology | -0.305 (-1.461; 0.852) | 0.559 | -0.545 | 0.591 | 301 | 0.0 | 0.047 |
| NYHA III/IV | -0.376 (-1.180; 0.428) | 0.394 | -0.955 | 0.347 | 1171 | 3.0 | 0.219 |
| ACE/ARB/ARNI | -0.271 (-1.246; 0.704) | 0.484 | -0.559 | 0.579 | 1298 | 0.0 | 0.210 |
| Beta Blockers | -0.340 (-1.237; 0.557) | 0.445 | -0.764 | 0.449 | 1280 | 0.0 | 0.216 |
| MRAs | -0.194 (-1.200; 0.811) | 0.490 | -0.397 | 0.694 | 887 | 0.0 | 0.125 |
| Diuretic | 0.015 (-0.932; 0.961) | 0.463 | 0.032 | 0.975 | 161 | 0.0 | 0.132 |
| LVEF Difference | 0.001 (-0.032; 0.034) | 0.016 | 0.059 | 0.953 | 783 | 0.0 | 0.212 |
| Follow Up Time | 0.043 (-0.045; 0.131) | 0.044 | 0.969 | 0.337 | 1059 | 1.3 | 0.159 |
| **CV mortality** | | | | | | | |
| Moderator | Coefficient (95% CI) | Standard error | Z value | P value | Q | R^2^ | Tau^2^ |
| Age | 0.042 (-0.001; 0.085) | 0.020 | 2.106 | 0.052 | 194 | 26.7 | 0.119 |
| BMI | 0.007 (-0.134; 0.149) | 0.064 | 0.115 | 0.911 | 480 | 0.0 | 0.208 |
| NT-proBNP | -0.104 (-0.126; -8195) x 10^-3^ | 9.378 x 10^-6^ | -11.103 | <0.001 | 15 | 100.0 | 1.568 x 10^-7^ |
| Male sex | -1.061 (-4.139; 2.018) | 1.444 | -0.734 | 0.474 | 563 | 0.0 | 0.167 |
| Hypertension | 1.129 (-0.936; 3.193) | 0.969 | 1.165 | 0.262 | 579 | 0.0 | 0.162 |
| AF | -0.581 (-2.012; 0.850) | 0.642 | -0.905 | 0.387 | 140 | 0.0 | 0.141 |
| Diabetes | 2.738 (0.092; 5.383) | 1.241 | 2.206 | 0.043 | 338 | 11.0 | 0.144 |
| Previous MI | 0.350 (-1.746; 2.447) | 0.909 | 0.385 | 0.710 | 53 | 0.0 | 0.185 |
| Ischemic Etiology | -1.104 (-7.304; 5.096) | 2.412 | -0.458 | 0.666 | 59 | 0.0 | 0.195 |
| NYHA III/IV | 0.256 (-1.156; 1.669) | 0.625 | 0.411 | 0.691 | 229 | 0.0 | 0.186 |
| ACE/ARB/ARNI | 1.209 (-0.473; 2.891) | 0.772 | 1.566 | 0.143 | 274 | 14.2 | 0.178 |
| Beta Blockers | -0.383 (-4.012; 3.245) | 1.649 | -0.232 | 0.820 | 187 | 0.0 | 0.270 |
| MRAs | -0.830 (-2.548; 0.887) | 0.771 | -1.077 | 0.307 | 417 | 0.0 | 0.186 |
| Diuretic | 0.194 (-1.544; 1.932) | 0.754 | 0.257 | 0.803 | 66 | 0.0 | 0.247 |
| LVEF Difference | 0.099 (0.003; 0.196) | 0.042 | 2.376 | 0.045 | 40 | 45.3 | 0.075 |
| Follow Up Time | 0.074 (-0.103; 0.251) | 0.083 | 0.887 | 0.388 | 202 | 0.0 | 0.160 |
| **HF hospitalization** | | | | | | | |
| Moderator | Coefficient (95% CI) | Standard error | Z value | P value | Q | R^2^ | Tau^2^ |
| Age | 0.041 (0.010; 0.072) | 0.015 | 2.716 | 0.011 | 799 | 18.9 | 0.200 |
| BMI | 0.025 (-0.073; 0.122) | 0.045 | 0.544 | 0.596 | 317 | 0.0 | 0.190 |
| NT-proBNP | -4.56 (-19.78; 10.65) x 10^-5^ | 6.914 x 10^-3^ | -0.660 | 0.523 | 907 | 0.0 | 0.380 |
| Male sex | -2.144 (-3.838; -0.449) | 0.829 | -2.587 | 0.015 | 715 | 17.8 | 0.203 |
| Hypertension | -0.512 (-2.085; 1.061) | 0.768 | -0.666 | 0.511 | 1115 | 0.0 | 0.249 |
| AF | 0.041 (-0.832; 0.915) | 0.417 | 0.099 | 0.922 | 329 | 0.0 | 0.120 |
| Diabetes | -0.352 (-2.690; 1.985) | 1.139 | -0.309 | 0.760 | 1119 | 0.0 | 0.255 |
| Previous MI | 0.430 (-1.028; 1.887) | 0.662 | 0.649 | 0.530 | 272 | 0.0 | 0.105 |
| Ischemic Etiology | -0.504 (-3.293; 2.285) | 1.209 | -0.417 | 0.688 | 76 | 0.0 | 0.161 |
| NYHA III/IV | -0.841 (-2.047; 0.366) | 0.563 | -1.494 | 0.157 | 610 | 9.1 | 0.269 |
| ACE/ARB/ARNI | -0.348 (-1.700; 1.005) | 0.654 | -0.531 | 0.600 | 915 | 0.0 | 0.302 |
| Beta Blockers | -0.669 (-1.986; 0.649) | 0.635 | -1.052 | 0.304 | 552 | 1.6 | 0.294 |
| MRAs | 0.040 (-1.113; 1.193) | 0.541 | 0.073 | 0.942 | 312 | 0.0 | 0.130 |
| Diuretic | 1.623 (0.472; 2.774) | 0.540 | 3.006 | 0.009 | 79 | 55.5 | 0.055 |
| LVEF Difference | -0.031 (-0.088; 0.026) | 0.028 | -1.122 | 0.274 | 880 | 2.2 | 0.271 |
| Follow Up Time | 0.024 (-0.134; 0.182) | 0.077 | 0.311 | 0.758 | 1141 | 0.0 | 0.254 |
| **In-hospital mortality** | | | | | | | |
| Moderator | Coefficient (95% CI) | Standard error | Z value | P value | Q | R^2^ | Tau^2^ |
| Age | 0.008 (-0.054; 0.071) | 0.027 | 0.308 | 0.766 | 28 | 0.0 | 0.139 |
| BMI | 0.060 (-0.084; 0.204) | 0.052 | 1.155 | 0.312 | 8 | 24.4 | 0.046 |
| LVEF Difference | 0.002 (-0.484; 0.489) | 0.175 | 0.014 | 0.989 | 16 | 0.0 | 0.319 |
| Male sex | -1.500 (-5.500; 2.501) | 1.769 | -0.848 | 0.418 | 30 | 0.0 | 0.109 |
| Hypertension | -0.055 (-3.097; 2.988) | 1.319 | -0.041 | 0.968 | 29 | 0.0 | 0.130 |
| AF | -0.151 (-0.563; 0.260) | 0.160 | -0.944 | 0.388 | 14 | 0.0 | 0.064 |
| Diabetes | 5.416 (-0.546; 11.378) | 2.585 | 2.095 | 0.069 | 21 | 38.7 | 0.062 |
| Previous MI | 7.223 (-30.657; 45.103) | 8.804 | 0.820 | 0.498 | 10 | 0.0 | 0.377 |
| Ischemic Etiology | 1.461 (-0.781; 3.704) | 0.873 | 1.675 | 0.155 | 8 | 45.0 | 0.019 |
| NYHA III/IV | 1.921 (-1.610; 5.452) | 1.110 | 1.731 | 0.182 | 13 | 37.1 | 0.260 |
| ACE/ARB/ARNI | -0.418 (-2.585; 1.750) | 0.940 | -0.444 | 0.669 | 29 | 0.0 | 0.130 |
| Beta Blockers | -0.465 (-2.570; 1.640) | 0.913 | -0.510 | 0.624 | 30 | 0.0 | 0.126 |
| MRAs | 0.014 (-0.611; 0.638) | 0.196 | 0.069 | 0.949 | 8 | 0.0 | 0.043 |
| Diuretic | 1.304 (-0.282; 2.890) | 0.671 | 1.944 | 0.093 | 24 | 18.4 | 0.085 |
| **Prior HF admission** | | | | | | | |
| Moderator | Coefficient (95% CI) | Standard error | Z value | P value | Q | R^2^ | Tau^2^ |
| Age | 0.000 (-0.026; 0.026) | 0.013 | 0.006 | 0.995 | 2606 | 0.0 | 0.085 |
| BMI | 0.037 (0.003; 0.071) | 0.016 | 2.318 | 0.034 | 992 | 21.7 | 0.048 |
| LVEF Difference | 0.026 (-0.018; 0.069) | 0.021 | 1.237 | 0.234 | 443 | 0.5 | 0.070 |
| Male sex | -0.604 (-2.461; 1.253) | 0.895 | -0.675 | 0.507 | 2577 | 0.0 | 0.082 |
| Hypertension | -0.008 (-0.830; 0.814) | 0.395 | -0.020 | 0.984 | 2450 | 0.0 | 0.088 |
| AF | 0.011 (-0.854; 0.875) | 0.413 | 0.026 | 0.980 | 2562 | 0.0 | 0.092 |
| Diabetes | 0.721 (-0.660; 2.101) | 0.666 | 1.082 | 0.291 | 1750 | 1.2 | 0.080 |
| Previous MI | 0.430 (-1.137; 1.998) | 0.703 | 0.611 | 0.555 | 914 | 0.0 | 0.080 |
| Ischemic Etiology | -0.687 (-2.037; 0.662) | 0.613 | -1.121 | 0.286 | 169 | 5.6 | 0.061 |
| NYHA III/IV | -0.128 (-0.680; 0.425) | 0.262 | -0.487 | 0.632 | 1842 | 0.0 | 0.088 |
| ACE/ARB/ARNI | -0.248 (-1.089; 0.593) | 0.400 | -0.619 | 0.544 | 2044 | 0.0 | 0.073 |
| Beta Blockers | 0.809 (-0.102; 1.720) | 0.435 | 1.858 | 0.079 | 2043 | 9.8 | 0.063 |
| MRAs | -0.611 (-1.759; 0.537) | 0.539 | -1.135 | 0.274 | 595 | 3.5 | 0.068 |
| Diuretic | 0.791 (-0.285; 1.868) | 0.498 | 1.589 | 0.136 | 1617 | 12.5 | 0.064 |
| **Length of hospital stay** | | | | | | | |
| Moderator | Coefficient (95% CI) | Standard error | Z value | P value | Q | R^2^ | Tau^2^ |
| Age | -0.002 (-0.010; 0.006) | 0.004 | -0.534 | 0.593 | 191 | 0.0 | 0.014 |
| BMI | 0.008 (-0.030; 0.047) | 0.020 | 0.417 | 0.676 | 73 | 0.0 | 0.040 |
| LVEF Difference | 0.001 (-0.007; 0.009) | 0.004 | 0.262 | 0.793 | 18 | 0.0 | 0.003 |
| NT-proBNP | -2.403 (-5.488; 0.682) x 10^-5^ | 1.574 x 10^-5^ | -1.526 | 0.127 | 84 | 20.9 | 0.004 |
| Male sex | 0.151 (-0.548; 0.850) | 0.357 | 0.423 | 0.672 | 221 | 0.0 | 0.014 |
| Hypertension | -0.151 (-0.409; 0.107) | 0.132 | -1.149 | 0.251 | 161 | 0.0 | 0.004 |
| AF | -0.129 (-0.425; 0.167) | 0.151 | -0.855 | 0.393 | 59 | 9.9 | 0.004 |
| Diabetes | -0.007 (-0.344; 0.330) | 0.172 | -0.041 | 0.968 | 220 | 0.0 | 0.014 |
| Previous MI | -1.262 (-1.833; -0.691) | 0.291 | -4.331 | <0.001 | 3 | 100.0 | 0.000 |
| Ischemic Etiology | 0.033 (-0.789; 0.855) | 0.419 | 0.078 | 0.938 | 82 | 0.0 | 0.021 |
| NYHA III/IV | 0.028 (-0.281; 0.338) | 0.158 | 0.179 | 0.858 | 100 | 0.0 | 0.006 |
| ACE/ARB/ARNI | -0.168 (-0.551; 0.215) | 0.195 | -0.859 | 0.390 | 210 | 3.5 | 0.016 |
| Beta Blockers | -0.120 (-0.596; 0.356) | 0.243 | -0.496 | 0.620 | 193 | 0.0 | 0.017 |
| MRAs | 0.177 (-0.170; 0.523) | 0.177 | 1.000 | 0.317 | 39 | 9.2 | 0.006 |
| Diuretic | -0.385 (-0.873; 0.104) | 0.249 | -1.543 | 0.123 | 61 | 19.5 | 0.004 |

**Abbreviations:** BMI, Body Mass Index; NT-proBNP, N-terminal pro-B-type Natriuretic Peptide; AF, Atrial Fibrillation; MI, Myocardial Infarction; NYHA III/IV, New York Heart Association Class III or IV; ACE/ARB/ARNI, Angiotensin-Converting Enzyme Inhibitors/Angiotensin Receptor Blockers/Angiotensin Receptor-Neprilysin Inhibitors; MRAs, Mineralocorticoid Receptor Antagonists; LVEF, Left Ventricular Ejection Fraction; CV, Cardiovascular; HF, Heart Failure.
