# Supplementary material for: Mortality and heart failure hospitalizations in heart failure with preserved ejection fraction compared to heart failure with reduced ejection fraction: a systematic review and meta-analysis
Source: ESC Heart Fail. 2026 Jan 16;13(1):xvag026. doi: 10.1093/eschf/xvag026 (PMC13108283; doi:10.1093/eschf/xvag026)
Supplement: xvag026_Supplementary_Data [file xvag026_supplementary_data.zip › TableS6.docx]

**Table S6**. Risk of bias of included cohort studies assessed using NOS tool.

| **Author, Year** | Selection | | | | Comparability | Outcome | | | Total score |
| --- | --- | --- | --- | --- | --- | --- | --- | --- | --- |
|  | Representativeness of the intervention cohort | Selection of the non-exposed cohort | Ascertainment of exposure | Demonstration that outcome of interest was not present at start of study | Control for important or additional factors | Assessment of outcome | Was follow up long enough for outcomes to occur | Adequacy of follow up of cohorts |  |
| **Abdul-Rahim 2018** | **⋆** | **⋆** | **⋆** | **⋆** | **⋆** | **⋆** | **⋆** |  | 7 |
| **Aelst 2018** |  | **⋆** | **⋆** | **⋆** |  | **⋆** | **⋆** | **⋆** | 6 |
| **Al-Jarallah 2020** |  | **⋆** | **⋆** | **⋆** | **⋆⋆** | **⋆** | **⋆** |  | 7 |
| **Anastasio 2022** | **⋆** | **⋆** | **⋆** | **⋆** | **⋆⋆** | **⋆** | **⋆** |  | 8 |
| **Bhatia 2006** | **⋆** | **⋆** | **⋆** | **⋆** | **⋆⋆** | **⋆** | **⋆** | **⋆** | 9 |
| **Bhatt 2024** | **⋆** | **⋆** | **⋆** | **⋆** |  | **⋆** | **⋆** | **⋆** | 7 |
| **Bonapace 2019** | **⋆** | **⋆** | **⋆** | **⋆** | **⋆⋆** | **⋆** | **⋆** | **⋆** | 9 |
| **Borovac 2019** | **⋆** | **⋆** | **⋆** | **⋆** | **⋆⋆** | **⋆** | **⋆** | **⋆** | 9 |
| **Bouwmeester 2022** | **⋆** | **⋆** | **⋆** | **⋆** |  | **⋆** | **⋆** |  | 6 |
| **Cenkerova 2016** | **⋆** | **⋆** | **⋆** | **⋆** | **⋆⋆** | **⋆** | **⋆** | **⋆** | 9 |
| **Chairat 2022** | **⋆** | **⋆** | **⋆** | **⋆** | **⋆⋆** |  | **⋆** | **⋆** | 8 |
| **Chung 2019** | **⋆** | **⋆** | **⋆** | **⋆** | **⋆** | **⋆** | **⋆** | **⋆** | 8 |
| **Cristobal 2023** |  | **⋆** | **⋆** | **⋆** | **⋆** | **⋆** | **⋆** |  | 6 |
| **Cui 2022** | **⋆** | **⋆** | **⋆** | **⋆** | **⋆⋆** | **⋆** | **⋆** | **⋆** | 9 |
| **Dunlay 2021** | **⋆** | **⋆** | **⋆** | **⋆** | **⋆⋆** | **⋆** | **⋆** | **⋆** | 9 |
| **Eitel 2019** | **⋆** | **⋆** | **⋆** | **⋆** | **⋆⋆** | **⋆** | **⋆** | **⋆** | 9 |
| **Farmakis 2017** | **⋆** | **⋆** | **⋆** | **⋆** | **⋆⋆** | **⋆** | **⋆** |  | 8 |
| **Farmakis 2023** | **⋆** | **⋆** | **⋆** | **⋆** | **⋆⋆** | **⋆** | **⋆** | **⋆** | 9 |
| **Fischer-Rasokat 2019** |  | **⋆** | **⋆** | **⋆** | **⋆** | **⋆** | **⋆** | **⋆** | 7 |
| **Frohlich 2019** | **⋆** | **⋆** | **⋆** | **⋆** | **⋆⋆** | **⋆** | **⋆** | **⋆** | 9 |
| **Fudim 2022** | **⋆** |  | **⋆** | **⋆** | **⋆⋆** | **⋆** |  |  | 6 |
| **Fujimoto 2022** | **⋆** | **⋆** | **⋆** | **⋆** | **⋆⋆** | **⋆** | **⋆** | **⋆** | 9 |
| **Ganapathi 2022** | **⋆** | **⋆** | **⋆** | **⋆** | **⋆⋆** | **⋆** | **⋆** | **⋆** | 9 |
| **Gargani 2021** | **⋆** | **⋆** | **⋆** | **⋆** | **⋆⋆** | **⋆** | **⋆** | **⋆** | 9 |
| **Gierula 2024** | **⋆** | **⋆** | **⋆** | **⋆** | **⋆⋆** | **⋆** | **⋆** | **⋆** | 9 |
| **Gomez-Otero 2017** | **⋆** | **⋆** | **⋆** | **⋆** | **⋆⋆** | **⋆** | **⋆** | **⋆** | 9 |
| **Gong 2022** | **⋆** | **⋆** | **⋆** | **⋆** | **⋆** | **⋆** | **⋆** | **⋆** | 8 |
| **Guo 2022** | **⋆** | **⋆** | **⋆** | **⋆** | **⋆⋆** | **⋆** | **⋆** | **⋆** | 9 |
| **Hamatani 2018** | **⋆** | **⋆** | **⋆** | **⋆** | **⋆⋆** | **⋆** | **⋆** | **⋆** | 9 |
| **Hamazaki 2022** | **⋆** | **⋆** | **⋆** | **⋆** | **⋆⋆** | **⋆** | **⋆** |  | 8 |
| **Huang 2020** | **⋆** | **⋆** | **⋆** | **⋆** | **⋆⋆** | **⋆** | **⋆** | **⋆** | 9 |
| **Huang 2022** | **⋆** | **⋆** | **⋆** | **⋆** | **⋆⋆** | **⋆** | **⋆** | **⋆** | 9 |
| **Imamura 2024** |  | **⋆** | **⋆** | **⋆** | **⋆⋆** |  |  | **⋆** | 6 |
| **Ito 2019** | **⋆** | **⋆** | **⋆** | **⋆** |  | **⋆** |  |  | 5 |
| **Iwatsu 2022** | **⋆** | **⋆** | **⋆** | **⋆** | **⋆** | **⋆** | **⋆** | **⋆** | 8 |
| **Jarkovsky 2022** | **⋆** |  | **⋆** | **⋆** | **⋆⋆** | **⋆** | **⋆** | **⋆** | 8 |
| **Jimenez-Marrero 2022** | **⋆** | **⋆** | **⋆** | **⋆** | **⋆⋆** | **⋆** | **⋆** |  | 8 |
| **Kamiya 2021** | **⋆** | **⋆** | **⋆** | **⋆** | **⋆⋆** | **⋆** | **⋆** |  | 8 |
| **Kapłon-Cieslicka 2016** | **⋆** | **⋆** | **⋆** | **⋆** | **⋆⋆** | **⋆** | **⋆** |  | 8 |
| **Kapłon-Cieslicka 2022** | **⋆** | **⋆** | **⋆** | **⋆** | **⋆⋆** | **⋆** | **⋆** |  | 8 |
| **Kasahara 2018** | **⋆** | **⋆** | **⋆** | **⋆** | **⋆⋆** | **⋆** | **⋆** |  | 8 |
| **Kawahira 2021** | **⋆** | **⋆** | **⋆** | **⋆** | **⋆⋆** | **⋆** | **⋆** | **⋆** | 9 |
| **Kawakami 2021** | **⋆** | **⋆** | **⋆** | **⋆** | **⋆⋆** | **⋆** | **⋆** | **⋆** | 9 |
| **Kerwagen 2023** | **⋆** | **⋆** | **⋆** | **⋆** | **⋆** | **⋆** | **⋆** |  | 7 |
| **Kim 2024** |  | **⋆** | **⋆** | **⋆** | **⋆⋆** | **⋆** | **⋆** | **⋆** | 8 |
| **Kitai 2022** | **⋆** | **⋆** | **⋆** | **⋆** | **⋆⋆** | **⋆** | **⋆** | **⋆** | 9 |
| **Kumar 2023** | **⋆** | **⋆** | **⋆** | **⋆** | **⋆⋆** | **⋆** | **⋆** | **⋆** | 9 |
| **Kusunose 2023** |  | **⋆** | **⋆** | **⋆** | **⋆⋆** | **⋆** | **⋆** | **⋆** | 8 |
| **Lala 2018** |  | **⋆** | **⋆** | **⋆** | **⋆** | **⋆** |  | **⋆** | 6 |
| **Lam 2018** | **⋆** | **⋆** | **⋆** | **⋆** | **⋆⋆** | **⋆** | **⋆** | **⋆** | 9 |
| **Lin 2019** | **⋆** | **⋆** | **⋆** | **⋆** | **⋆⋆** | **⋆** | **⋆** | **⋆** | 9 |
| **Lofman 2017** | **⋆** | **⋆** | **⋆** | **⋆** | **⋆⋆** | **⋆** | **⋆** | **⋆** | 9 |
| **Lopez-Azor 2023** |  | **⋆** | **⋆** | **⋆** | **⋆⋆** | **⋆** | **⋆** |  | 7 |
| **Lund 2018** | **⋆** |  | **⋆** | **⋆** | **⋆⋆** | **⋆** | **⋆** | **⋆** | 8 |
| **Lyu 2019** | **⋆** | **⋆** | **⋆** | **⋆** | **⋆⋆** | **⋆** | **⋆** | **⋆** | 9 |
| **Mansur 2022** | **⋆** | **⋆** | **⋆** | **⋆** | **⋆⋆** | **⋆** |  |  | 7 |
| **Migas 2024** | **⋆** | **⋆** | **⋆** | **⋆** | **⋆** | **⋆** | **⋆** | **⋆** | 8 |
| **Miller 2016** | **⋆** | **⋆** | **⋆** | **⋆** |  | **⋆** | **⋆** | **⋆** | 7 |
| **Miro 2023** | **⋆** | **⋆** | **⋆** | **⋆** | **⋆⋆** | **⋆** | **⋆** | **⋆** | 9 |
| **Mirzai 2023** | **⋆** | **⋆** | **⋆** | **⋆** | **⋆⋆** | **⋆** | **⋆** |  | 8 |
| **Nakamaru 2023** | **⋆** | **⋆** | **⋆** | **⋆** | **⋆⋆** | **⋆** | **⋆** | **⋆** | 9 |
| **Nichols 2015** | **⋆** | **⋆** | **⋆** | **⋆** |  | **⋆** | **⋆** |  | 6 |
| **Niedziela 2024** | **⋆** | **⋆** | **⋆** | **⋆** |  | **⋆** | **⋆** | **⋆** | 7 |
| **Ou 2023** |  | **⋆** | **⋆** | **⋆** | **⋆⋆** |  | **⋆** |  | 6 |
| **Pagnesi 2023** | **⋆** | **⋆** | **⋆** | **⋆** | **⋆⋆** | **⋆** |  |  | 7 |
| **Pan 2022** | **⋆** | **⋆** | **⋆** | **⋆** | **⋆⋆** | **⋆** | **⋆** | **⋆** | 9 |
| **Popovic 2018** | **⋆** | **⋆** | **⋆** | **⋆** | **⋆⋆** | **⋆** | **⋆** |  | 8 |
| **Santas 2022** | **⋆** | **⋆** | **⋆** | **⋆** | **⋆⋆** |  | **⋆** |  | 7 |
| **Scrutinio 2023** | **⋆** | **⋆** | **⋆** | **⋆** | **⋆⋆** | **⋆** | **⋆** | **⋆** | 9 |
| **Seckin 2023** | **⋆** | **⋆** | **⋆** | **⋆** | **⋆⋆** | **⋆** | **⋆** | **⋆** | 9 |
| **Settergren 2024** | **⋆** | **⋆** | **⋆** | **⋆** | **⋆⋆** | **⋆** | **⋆** | **⋆** | 9 |
| **Shiga 2019** | **⋆** | **⋆** | **⋆** | **⋆** | **⋆⋆** |  | **⋆** |  | 7 |
| **Shukkoor 2021** | **⋆** | **⋆** | **⋆** | **⋆** |  |  | **⋆** |  | 5 |
| **Song 2022** | **⋆** | **⋆** | **⋆** | **⋆** | **⋆⋆** | **⋆** | **⋆** |  | 8 |
| **Takei 2019** | **⋆** | **⋆** | **⋆** | **⋆** | **⋆** | **⋆** | **⋆** | **⋆** | 8 |
| **Tay 2023** | **⋆** | **⋆** | **⋆** | **⋆** | **⋆⋆** |  | **⋆** | **⋆** | 8 |
| **Thuijs 2022** | **⋆** | **⋆** | **⋆** | **⋆** | **⋆⋆** | **⋆** | **⋆** | **⋆** | 9 |
| **Tomasoni 2024** | **⋆** | **⋆** | **⋆** | **⋆** | **⋆⋆** | **⋆** | **⋆** |  | 8 |
| **Tromp 2017** |  | **⋆** | **⋆** | **⋆** | **⋆** |  | **⋆** |  | 5 |
| **Tromp 2024** | **⋆** | **⋆** | **⋆** | **⋆** | **⋆⋆** | **⋆** | **⋆** | **⋆** | 9 |
| **Tsuji 2017** | **⋆** | **⋆** | **⋆** | **⋆** | **⋆⋆** |  | **⋆** | **⋆** | 8 |
| **van Essen 2022** |  | **⋆** | **⋆** | **⋆** | **⋆⋆** | **⋆** | **⋆** |  | 7 |
| **Wang 2017** | **⋆** | **⋆** | **⋆** | **⋆** | **⋆⋆** |  | **⋆** |  | 7 |
| **Wang 2024** | **⋆** | **⋆** | **⋆** | **⋆** | **⋆⋆** | **⋆** | **⋆** | **⋆** | 9 |
| **Wierda 2023** |  | **⋆** |  | **⋆** | **⋆⋆** | **⋆** | **⋆** |  | 6 |
| **Wu 2023** |  | **⋆** | **⋆** | **⋆** | **⋆⋆** | **⋆** |  |  | 6 |
| **Xu 2014** | **⋆** | **⋆** | **⋆** | **⋆** | **⋆** | **⋆** | **⋆** | **⋆** | 8 |
| **Xu 2022** | **⋆** | **⋆** | **⋆** | **⋆** | **⋆⋆** |  | **⋆** | **⋆** | 8 |
| **Yaku 2018** | **⋆** | **⋆** | **⋆** | **⋆** |  | **⋆** | **⋆** |  | 6 |
| **Yoshikawa 2022** | **⋆** | **⋆** | **⋆** | **⋆** | **⋆⋆** | **⋆** | **⋆** | **⋆** | 9 |
| **Zafrir 2019** | **⋆** | **⋆** | **⋆** | **⋆** | **⋆⋆** | **⋆** | **⋆** |  | 8 |
| **Zeller 2021** | **⋆** | **⋆** | **⋆** | **⋆** | **⋆** | **⋆** | **⋆** | **⋆** | 8 |
| **Zhirov 2019** |  | **⋆** | **⋆** | **⋆** |  |  |  |  | 3 |
