# Supplementary material for: Mortality and heart failure hospitalizations in heart failure with preserved ejection fraction compared to heart failure with reduced ejection fraction: a systematic review and meta-analysis
Source: ESC Heart Fail. 2026 Jan 16;13(1):xvag026. doi: 10.1093/eschf/xvag026 (PMC13108283; doi:10.1093/eschf/xvag026)
Supplement: xvag026_Supplementary_Data [file xvag026_supplementary_data.zip › TableS7.docx]

**Table S7**. Risk of bias of included cross-sectional studies assessed using modified NOS tool.

| **Author, Year** | Selection | | | Comparability | Outcome | | Total score |
| --- | --- | --- | --- | --- | --- | --- | --- |
|  | Representativeness of the intervention cohort | Sample size | Ascertainment of exposure | Control for important or additional factors | Assessment of outcome | Statistical test |  |
| **Beale 2019** |  | **⋆** | **⋆** | **⋆⋆** | **⋆** | **⋆** | 6 |
| **Chandra 2019** |  | **⋆** | **⋆** | **⋆⋆** | **⋆** | **⋆** | 6 |
| **Gok 2022** | **⋆** | **⋆** | **⋆** |  | **⋆** |  | 4 |
| **Hage 2022** | **⋆** |  | **⋆** | **⋆⋆** | **⋆** | **⋆** | 6 |
| **Hamada 2022** | **⋆** | **⋆** | **⋆** | **⋆⋆** | **⋆** | **⋆** | 7 |
| **Rywik 2022** |  | **⋆** |  | **⋆⋆** |  | **⋆** | 4 |
| **Smeets 2020** | **⋆** | **⋆** | **⋆** |  | **⋆** | **⋆** | 5 |
| **Subki 2020** | **⋆** | **⋆** | **⋆** |  |  | **⋆** | 4 |
| **Wernhart 2023** | **⋆** | **⋆** | **⋆** | **⋆⋆** | **⋆** | **⋆** | 7 |
